# Activation of invasion by oncogenic reprogramming of cholesterol metabolism via increased NPC1 expression and macropinocytosis

**DOI:** 10.1038/s41388-023-02771-x

**Published:** 2023-07-07

**Authors:** Aikaterini Skorda, Anna Røssberg Lauridsen, Chengnan Wu, Jinrong Huang, Monika Mrackova, Nuggi Ingholt Winther, Vanessa Jank, Zsofia Sztupinszki, Robert Strauss, Mesut Bilgin, Kenji Maeda, Bin Liu, Yonglun Luo, Marja Jäättelä, Tuula Kallunki

**Affiliations:** 1 Cancer Invasion and Resistance, Danish Cancer Institute, Strandboulevarden 49, 2100 Copenhagen, Denmark; 2 Department of Biomedicine, Aarhus University, Aarhus, Denmark; 3 Translational Cancer Genomics, Danish Cancer Institute, Copenhagen, Denmark; 4 Genome Integrity Group, Danish Cancer Institute, Copenhagen, Denmark; 5 Lipidomics Core Facility, Danish Cancer Institute, Copenhagen, Denmark; 6 Cell Death and Metabolism, Center for Autophagy, Recycling and Disease, Danish Cancer Institute, Copenhagen, Denmark; 7 Steno Diabetes Center Aarhus, Aarhus University Hospital, Aarhus, Denmark; 8 Department of Cellular and Molecular Medicine, Faculty of Health and Medical Sciences, University of Copenhagen, Copenhagen, Denmark; 9 Department of Drug Design and Pharmacology, Faculty of Health and Medical Sciences, University of Copenhagen, Copenhagen, Denmark

**Keywords:** Cellular imaging, Oncogenes

## Abstract

Cancer cells are dependent on cholesterol, and they possess strictly controlled cholesterol homeostasis mechanisms. These allow them to smoothly switch between cholesterol synthesis and uptake to fulfill their needs and to adapt environmental changes. Here we describe a mechanism of how cancer cells employ oncogenic growth factor signaling to promote uptake and utilization of extracellular cholesterol via Myeloid Zinc Finger 1 (MZF1)-mediated Niemann Pick C1 (NPC1) expression and upregulated macropinocytosis. Expression of p95ErbB2, highly oncogenic, standard-treatment resistant form of ErbB2 mobilizes lysosomes and activates EGFR, invasion and macropinocytosis. This is connected to a metabolic shift from cholesterol synthesis to uptake due to macropinocytosis-enabled flow of extracellular cholesterol. NPC1 increase facilitates extracellular cholesterol uptake and is necessary for the invasion of ErbB2 expressing breast cancer spheroids and ovarian cancer organoids, indicating a regulatory role for NPC1 in the process. The ability to obtain cholesterol as a byproduct of increased macropinocytosis allows cancer cells to direct the resources needed for the energy-consuming cholesterol synthesis towards other activities such as invasion. These results demonstrate that macropinocytosis is not only an alternative energy source for cancer cells but also an efficient way to provide building material, such as cholesterol, for its macromolecules and membranes.

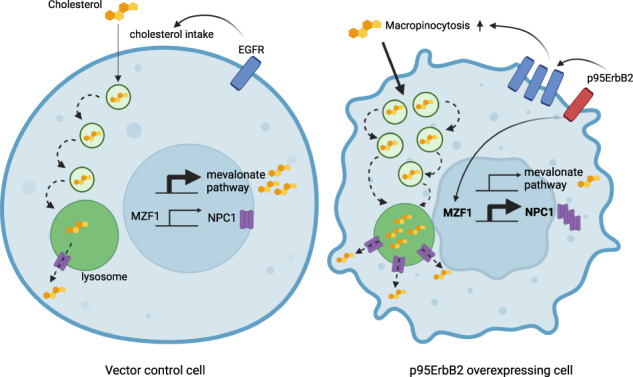

## Introduction

Cholesterol is an essential lipid and necessary for cell viability and growth, thus its homeostasis is tightly regulated [1–5]. Cholesterol is a precursor for steroids, and involved in the biosynthesis of estrogen, androgen, and vitamin D. It provides building blocks for cellular membranes and induces the formation of cholesterol-rich signaling platforms, lipid rafts, through which it can activate the cancer associated Hedgehog, Wnt and mTOR signaling pathways. Results of epidemiological studies concerning increased circulatory cholesterol and cancer progression have been inconsistent [1]. However, recent studies involving over 200000 breast cancer patients shows a statistically significant correlation between increased breast cancer risk and high levels of cholesterol carrier proteins, high- and low-density lipoproteins (HDL and LDL), independently of tumor estrogen receptor status [6–8], suggesting a connection between cholesterol uptake and cancer progression. Latest studies promote for a central role for the intracellular cholesterol in the growth and invasiveness of cancer cells and tumors [1, 9, 10].

Cancer cells maintain their cholesterol homeostasis by regulating its synthesis and uptake [1]. Although the cholesterol synthesis pathway and its regulation are documented in details [11], less information exists on the mechanisms regulating cancer cells uptake of extracellular cholesterol. The best studied cellular cholesterol uptake mechanism is the endocytosis of LDL-cholesterol bound to the LDL-receptor [12]. Cancer cells can also acquire extracellular cholesterol, especially HDL-cholesterol, via scavenger receptor class B type 1 (SR-B1 that is encoded by *SCARB1* gene) [13]. SR-B1 expression is often increased in cancer, and its increased expression in breast cancer is associated with worse clinico-pathological status and shorter survival [13].

Proper lysosome function is fundamental for cholesterol homeostasis, since it is necessary for the cells ability to utilize extracellular cholesterol [14, 15]. Extracellular cholesterol/cholesteryl ester is transported to lysosomes via endocytic vesicles. Cholesteryl esters enter the endocytic pathway as constituents of HDL or LDL lipoprotein particles and are hydrolysed into free cholesterol molecules by the acid lipase (LAL) inside the lysosomal lumen. The major intracellular cholesterol transporter and transmembrane protein Niemann-Pick C1 (NPC1) is located at the lysosomal limiting membrane, and it is responsible for the efflux of free cholesterol from lysosomes to the cytosol [15, 16]. Upon release from lysosome, cholesterol is transported to its destination, making NPC1 as a key gatekeeper for the cellular utilization of extracellular cholesterol. Most of the intracellular cholesterol is transported to the plasma membrane, where it controls membrane fluidity and formation of lipid rafts. Lysosomes have an active role in cancer and exocytosed lysosomal hydrolases can initiate and facilitate extracellular matrix degradation and invasion of cancer cells [17–19]. MZF1 is a transcription factor that regulates ErbB2-induced invasion of breast cancer cells by inducing expression of cathepsin B (*CTSB*) and the outward transport of lysosomes to the plasma membrane, where the exocytosis of cathepsin B and other lysosomal hydrolases contribute to cancer invasion [17].

Prompted by our discovery showing that induced expression and activation of p95ErbB2 regulates genes involved in cholesterol uptake and trafficking, we studied if and how cholesterol uptake is involved in ErbB2-mediated cancer cell invasion. Our results showed that expression of constitutively active and standard treatment resistant p95ErbB2 in breast cancer cells induces macropinocytosis and increases the expression of cholesterol trafficking protein NPC1 and results in increased extracellular cholesterol uptake. For cholesterol uptake experiments, we used U18666A, an inhibitor of NPC1-mediated efflux of cholesterol from lysosomes, which resulted in accumulation of cholesterol in lysosomes and enabled its image-based quantification. We used filipin III fluorescent staining of carrier free cholesterol as well as live imaging of extracellularly administered fluorescent BODIPY-cholesterol to visualize and quantify lysosomal cholesterol levels with automated high-throughput fluorescent microscopy. CRISPR-Cas9, RNA sequencing (RNA-seq), chromatin immunoprecipitation (ChIP) and small interfering RNA (siRNA) were used to identify ErbB2-inducible, oncogenic transcription factor MZF1 as a potential regulator of NPC1 expression. Administration of high molecular weight (70 kDa) dextran and 5-(N-ethyl-N-isopropyl)amiloride (EIPA) indicated that macropinocytosis was a major mechanism responsible for extracellular cholesterol uptake. Upregulation of NPC1 expression was sufficient to increase cholesterol uptake. Invasion assays utilizing cancer cell spheroids and tumor organoids revealed an invasion promoting role of NPC1 and extracellular cholesterol.

## Results

### p95ErbB2 increases expression of *NPC1*, induces invasion and attenuates cholesterol synthesis genes

**Fig. 1:**
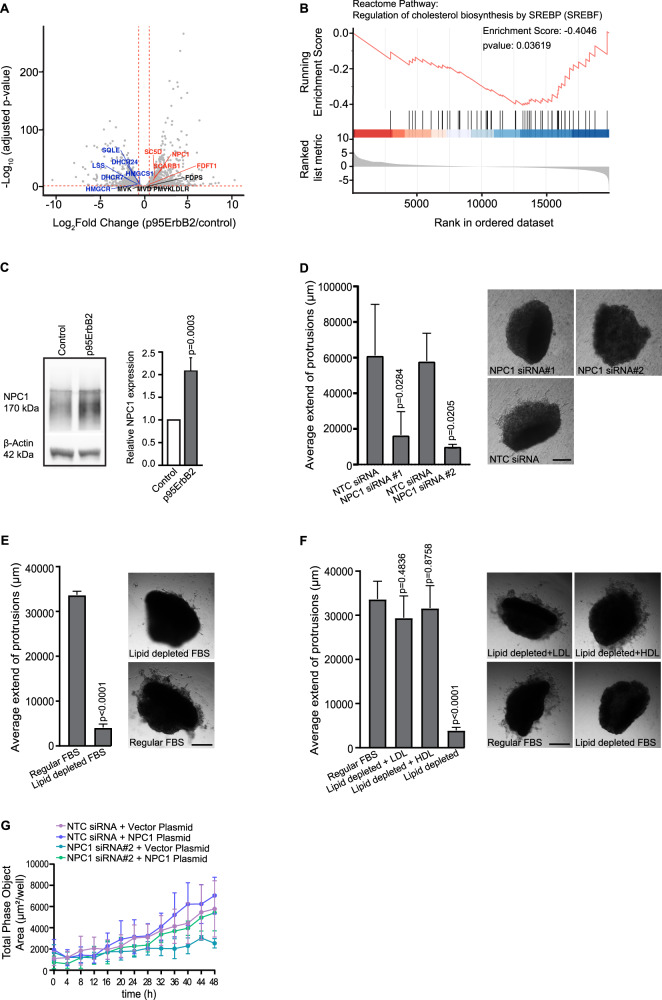
p95ErbB2 induces expression of *NPC1*, which induces invasion and attenuates cholesterol synthesis genes. **A** Volcano blot presenting the RNA sequencing results of p95ErbB2 and corresponding control cells with focus on the expression of selected cholesterol metabolism genes. Expression of *NPC1* and *SCARB1* (red), and the expression of selected cholesterol synthesis regulating genes (blue). **B** GSEA analysis comparing p95ErbB2 expression and the corresponding control cell lines. Enrichment plot represents "Reactome Regulation of cholesterol biosynthesis by SREBP (SREBF)"-pathway. False discovery rate (FDR) is 0.1827. Genes involved are *ACACA, ACACB, CARM1, CHD9, CREBBP, CYP51A1, DHCR7, ELOVL6, FASN, FDFT1, FDPS, GGPS1, GPAM, HELZ2, HMGCR, HMGCS1, IDI1, INSIG1, INSIG2, KPNB1, LSS, MBTPS1, MBTPS2, MED1, MTF1, MVD, MVK, NCOA1, NCOA2, NCOA6, NFYA, NFYB, NFYC, PMVK, PPARA, RAN, RXRA, SAR1B, SC5D, SCAP, SCD, SEC23A, SEC24A, SEC24B, SEC24C, SEC24D, SMARCD3, SP1, SQLE, SREBF1, SREBF2, TBL1X, TBL1XR1, TGS1, TM7SF2*. **C** Representative immunoblot comparing NPC1 expression in p95ErbB2 and corresponding control cells. β-actin is a loading control (left). Quantification of immunoblots based on *n* = 3 biological experiments (right). **D** *NPC1* siRNA-treatment as an inhibitor of invasion of p95ErbB2 spheroids. Quantification of the length of the invasive protrusions of cell spheroids 72 h after transfection with NTC siRNA, *NPC1* siRNAs #1 and #2 (left). Representative images of siRNA transfected spheroids. Spheroids are imaged 48 h after embedding in Matrigel (right). Scale bar is 250 μm. **E** Invasiveness of p95ErbB2 spheroids in lipid depleted medium. Quantification of the length of the invasive protrusions of p95ErbB2 spheroids after 48 h culture in medium with lipid depleted FBS and in regular FBS medium (control) (left). Representative images (right). Scale bar is 250 μm. **F** Rescue of the invasiveness in lipid depleted medium supplemented with LDL or HDL. Quantification of the length of the invasive protrusions of p95ErbB2 spheroids after 48 h culture in medium supplemented with lipid depleted FBS + LDL, or +HDL and in regular FBS medium and in lipid depleted FBS control (left). LDL and HDL were added in concentrations equal to their concentration in total cholesterol of the regular FBS (measured in Supplementary Fig. 1F). Representative images of invasive spheroids (right). Scale bar is 250 μm. Graph bars in **D**, **E** and **F** represent the mean ± SD of *n* = 3 biological repeats. Four spheroids of each biological repeat were quantified per treatment. *P* values for **D**, **E** and **F** are calculated by one-way ANOVA. Differences are considered significant at values *p* ≤ 0.05. **G** Transmembrane invasion assay of *NPC1* siRNA and plasmid transfected cells and their corresponding control constructs. Membranes were coated with BME matrix and the appearance of cells capable of invading through matrix were quantified as the area covered by cells on the chemoattractant side of the membrane. The area covered by cells was blotted as a function of time. Each point presents the mean ± SEM from *n* = 3 biological repeats with triplicate conditions.

Induced expression of p95ErbB2, a constitutively active, N-terminally truncated form of ErbB2 leads into rapid initiation of invasion [17, 20]. Its expression defines a clinically challenging form of an aggressive breast cancer, which is non-responsive to the standard ErbB2-targeting immunotherapy with trastuzumab and pertuzumab because it lacks their binding sites. We set up an RNA-seq experiment to understand biological processes leading to the activation of invasion by this constitutively active oncogene. We were specifically interested in lysosomes and their function in ErbB2-induced invasion and thus also in the activation of genes whose expression could be upregulated by MZF1, an ErbB2-inducible transcription factor that mediates p95ErbB2-induced early lysosome-dependent invasive changes [17, 21]. Of genes with reported lysosomal function [22] that are potentially activated by p95ErbB2 and attenuated by MZF1 depletion with CRISPR-Cas9 (RNA-seq results in https://db.cngb .org/search /project/CNP0003166/), we identified seven candidates matching these criteria (*ADA, AP3B2, CD68, IFI30, MFSD1, NPC1* and *SCARB1*; Supplementary Fig. 1A). This included two central genes of cholesterol transport and utilization of extracellular cholesterol: The lysosomal cholesterol transporter *NPC1* and scavenger receptor *SCARB1*. On the contrary, the expression of several of the enzymes of the major cholesterol synthesis pathway, mevalonate pathway, including *HMGCR*, the rate limiting enzyme 3-hydroxy-3-methylglutaryl coenzyme A (HMG-CoA) reductase, were reduced. (Fig. 1A, Supplementary Fig. 1B). These results suggest that the cells had likely switched from cholesterol synthesis to its uptake upon p95ErbB2 activation.

Sterol regulatory element binding proteins SREBPs/SREFBs are transcription factors that regulate biosynthesis of steroids and the expression of cholesterol metabolism genes [23]. Expression of SREBP/SREFB-regulated cholesterol biosynthesis genes was significantly reduced by p95ErbB2 expression (Fig. 1B). Significant attenuation of cholesterol biosynthesis regulating genes was also identified (Supplementary Fig. 1C). Supplementary Table 1 shows differentially expressed genes (DEG) with fold change ≥1.5 and FDR ≤ 0.05 using DESeq2 and Supplementary Table 2 shows GSEA pathways with *p*-value < 0.05.

p95ErbB2 expression increased the expression of NPC1 protein (Fig. 1C, Supplementary Fig. 1D). To investigate if increased NPC1 expression was involved in invasiveness, we set up a 3-dimensional (3D) spheroid invasion assay using p95ErbB2 expressing spheroids transfected with two different *NPC1* siRNAs, or with a corresponding non-targeting control (NTC) siRNA (siRNA efficiencies; Supplementary Fig. 1E). Expression of *NPC1* siRNAs inhibited invasion of spheroids into Matrigel significantly 72 h after transfection (Fig. 1D), corresponding 48 h culturing of spheroids. Supporting the importance of uptake of the extracellular cholesterol, invasion was inhibited in tissue culture media supplemented with lipid depleted serum (Fig. 1E; Supplementary Fig. 1F) and restored by supplementing lipid depleted serum with LDL or HDL (Fig. 1F). Expression of invasion markers MMP13 and fibronectin was detected in p95ErbB2 expressing cells (Supplementary Fig. 2A). Depletion of NPC1 didn´t decrease their expression indicating that NPC1 was not regulating their expression, but depletion of MZF1 led into downregulation of Fascin-1 and MMP13 (Supplementary Fig. 2B–D). Immunofluoresence analysis by quantitative image-based cytometry (QIBC) indicated significant cytosolic Fascin-1 and NPC1 downregulation in MZF1-depleted cells (Supplementary Fig. 2D). Expression of NPC1 plasmid in NPC1-depleted cells resulted in a complete invasion rescue (Fig. 1G). Moreover, mere overexpression of NPC1 increased invasion (Fig. 1G). These results indicate that the switch from the expression of cholesterol synthesis genes to cholesterol uptake gene *NPC1* coincided with increased cholesterol- and NPC1-dependent invasion.

### Increased NPC1 expression is associated with MZF1-regulated, increased cholesterol uptake and lysosomal accumulation

**Fig. 2:**
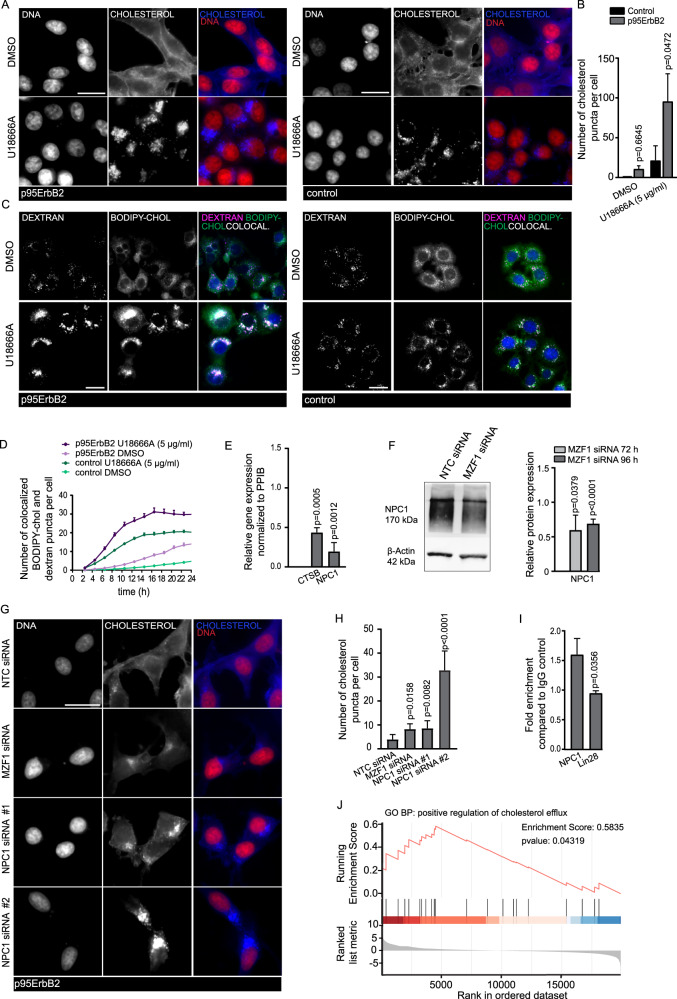
Increased NPC1 expression is associated with MZF1-regulated, increased cholesterol uptake and lysosomal accumulation. **A** Representative images of filipin III-stained free cholesterol in p95ErbB2 (images at left) and control cells (images at right) after treatment with DMSO or 5 μg/ml of U18666A for 24 h. Scale bar is 25 μm, *n* = 3 independent experiments. **B** Quantification of images in **A**. Bars are mean ± SD of *n* = 9 images acquired per triplicate of each condition from 3 biological experiments. **C** Representative images of cellular uptake of extracellularly administered BODIPY-cholesterol and its lysosomal accumulation (indicated as white areas) in p95ErbB2 cells (left side images) or corresponding control cells (right side images) treated with DMSO or U18666A at 16 h timepoint. Pretreatment with 10 kDa fluorescent dextran was used to mark the lysosomes. Scale bar is 25 μm, *n* ≥ 3 independent experiments. **D** Quantification of **C**. Cells were continuously imaged every 2 h for 24 h and the number of co-localized 10 kDa dextran (indicating lysosomes) and BODIPY-cholesterol puncta per cell was blotted as a function of time. Each point presents the mean ± SEM of 9 images acquired per triplicate condition from 3 biological experiments. **E** Quantitative RT-PCR measurement of the relative *NPC1* gene expression in *MZF1* siRNA depleted (20 nM for 72 h) p95ErbB2 cells. Gene expression was normalized to *PPIB*. NTC siRNA was used as a negative control. Attenuation of *CTSB* expression with *MZF1* siRNA was used as a positive control. Bars are mean ± SD, *n* = 3 biological experiments. **F** Representative immunoblot showing NPC1 expression in *MZF1* siRNA transfected (20 nM for 96 h) and corresponding non-targeted control p95ErbB2 cells (left). Quantification of immunoblots based on data of *n* = 3 biological experiments at 72 and 96 h after transfections (right). **G** Representative images of filipin III-stained free cholesterol accumulation in *MZF1*, *NPC1*#1 and *NPC1*#2 siRNA transfected (20 nM; 96 h) p95ErbB2 cells. *n* = 3 biological experiments. Scale bar is 25 μm. **H** Quantification of *n* = 6–9 images of **G** acquired per triplicate wells of each condition and from 3 biological experiments. **I** Chromatin Immunoprecipitation assay (ChIP). Representative quantification of ChIP of *n* = 3 biological experiments. *LIN28A* promoter region with no NPC1 binding sites was used as a control. **J** GSEA analysis of p95ErbB2-regulated genes. Enrichment plot represents "GOBP (Gene Ontology Biological Process) Positive regulation of cholesterol efflux" pathway. FDR 0.3422. Genes involved are *ABCA1, ABCA12, ABCA3, ABCA7, ABCA8, ABCG1, ABCG4, ADIPOQ, APOA1, APOE, CAV1, CES1, CES1, EEPD1, GPS2, GPS2, LRP1, MIR206, NFKBIA, NR1H2, NR1H3, PLTP, PON1, PPARG, PTCH1, RXRA, SIRT1, TREM2, ZDHHC8*. Statistical significance (p) was calculated for **B** and **F** by unpaired, two-tailed Student’s *t*-test and for **B**, **F** and **I** and for **H** by one-way ANOVA combined with Dunnett’s multiple comparisons. Differences are considered significant at values *p* ≤ 0.05.

We investigated whether increased expression of NPC1 has a functional role in cholesterol transport. For this, we used U18666A, which inhibits the ability of NPC1 to transport free cholesterol from lysosomes to the cytosol resulting in cholesterol accumulation in lysosomes. U18666A-treated p95ErbB2 cells expressing high levels of NPC1 accumulated significantly higher levels of cholesterol in lysosomes than the corresponding U18666A-treated control cells. This was evident both by the staining of the intracellular cholesterol with the free cholesterol binding dye filipin III (Fig. 2A, B), and administration of the fluorescent BODIPY-cholesterol to the growth medium (Fig. 2C, D). Comparison of *MZF1* siRNA [24] to the NTC siRNA supported the RNA sequencing results showing that the NPC1 expression was partially dependent on MZF1 both for its RNA (Fig. 2E) and for protein expression (Fig. 2F, Supplementary Fig. 3A). Depletion of either MZF1 or NPC1 resulted in significant accumulation of cholesterol (Fig. 2G, H). Connection between MZF1 and NPC1 was further supported by QIBC analysis where MZF1-depleted cells showed significant reduction of cytosolic NPC1 (Supplementary Fig. 2D). ChIP assay suggested MZF1 binding to the promoter region of *NPC1* (Fig. 2I). Gene Ontology Biological Process (GOBP) analysis indicated increased expression of the positive regulation of cholesterol efflux pathway genes in p95ErbB2 expressing cells, anticipating activation of cholesterol trafficking (Fig. 2J). These results identify MZF1 as a regulator of p95ErbB2-induced, increased *NPC1* expression and indicate that the activation of ErbB2 signaling and NPC1 is involved in the cholesterol metabolism of these cells.

### Expression of p95ErbB2 activates macropinocytosis

**Fig. 3:**
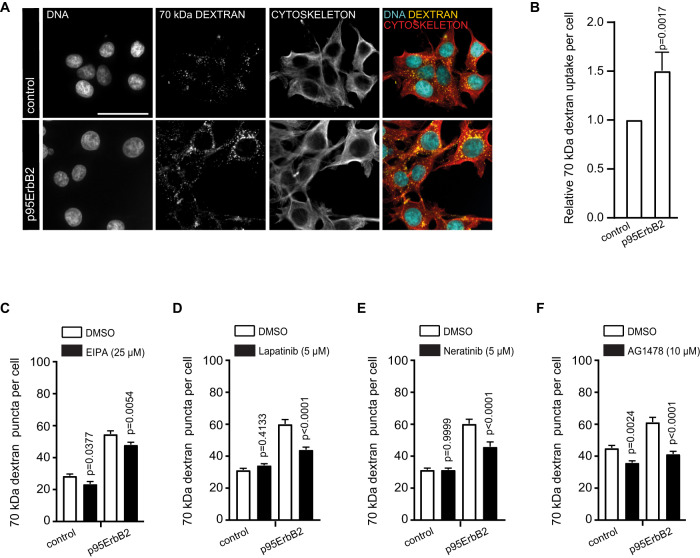
Expression of p95ErbB2 activates macropinocytosis. **A** Representative images of accumulation of 70 kDa dextran (2 h incubation) in p95ErbB2 and control cells. Scale bar is 50 μm. **B** Quantification of relative dextran uptake per cell of the images from **A**. Bars are mean ± SD of *n* = 3 biological experiments. **C** Quantification as in **B** of images of accumulation of 70 kDa dextran in p95ErbB2 and control cells after treatment with EIPA (25 μM) or DMSO. **D** Quantification of images as in **B** of accumulation of 70 kDa dextran in p95ErbB2 and control cells after treatment with lapatinib (5 μM) or DMSO. **E** Quantification of images as in **B** of accumulation of 70 kDa dextran in p95ErbB2 and control cells after treatment with neratinib (5 μM) or DMSO. **F** Quantification of images as in **B** of accumulation of 70 kDa dextran in p95ErbB2 cells and control cells after treatment with AG1478 (10 μM) or DMSO. In each case (**B**–**F**) *n* = 9 images were quantified from triplicate wells of each condition from 3 biological experiments. Statistical significance (*p* value) for **B** was calculated by unpaired, two-tailed Student’s *t*-test and one-way ANOVA combined with Dunnett’s multiple comparisons was used for **C**, **D**, **E** and **F**. Differences are considered significant at values p ≤ 0.05.

Epidermal growth factor receptor (EGFR) is one of the most common activators of macropinocytosis, an endocytotic process that is regulated by actin cytoskeleton [25]. Activation of ErbB2 and especially p95ErbB2, activate EGFR signaling and membrane ruffling [20], raising the possibility that the p95ErbB2-induced EGFR expression (Supplementary Fig. 3B, C) could activate macropinocytosis. Macropinocytosis assay measuring the intracellular accumulation of extracellularly administered high molecular weight, 70 kDa fluorescent dextran [26], indicated that the dextran uptake was significantly higher in p95ErbB2 expressing than in control cells (Fig. 3A, B). This coincided with the increased expression of EGFR (Supplementary Fig. 3B, C). Activation of macropinocytosis was verified with macropinocytosis inhibitor EIPA [27], which significantly inhibited appearance of dextran puncta even in a low (25 μM), non-toxic concentration (Fig. 3C; 50 μM EIPA, standardly used to inhibit macropinocytosis, was highly toxic). Treatment of p95ErbB2 cells with moderate concentrations of clinical ErbB2 inhibitors lapatinib (Fig. 3D) or neratinib (Fig. 3E), which primarily target ErbB2 and to minor extent EGFR, decreased dextran uptake. Treatment with a clinical EGFR inhibitor AG1478 (Fig. 3F) instead inhibited dextran uptake significantly in p95ErbB2 expressing and in control cells, both of which express endogenous EGFR, indicating its efficiency against EGFR-regulated macropinocytosis. These results support the activation of the EGFR-induced macropinocytosis by p95ErbB2 activation.

### Macropinocytosis and NPC1 increase cholesterol transport

**Fig. 4:**
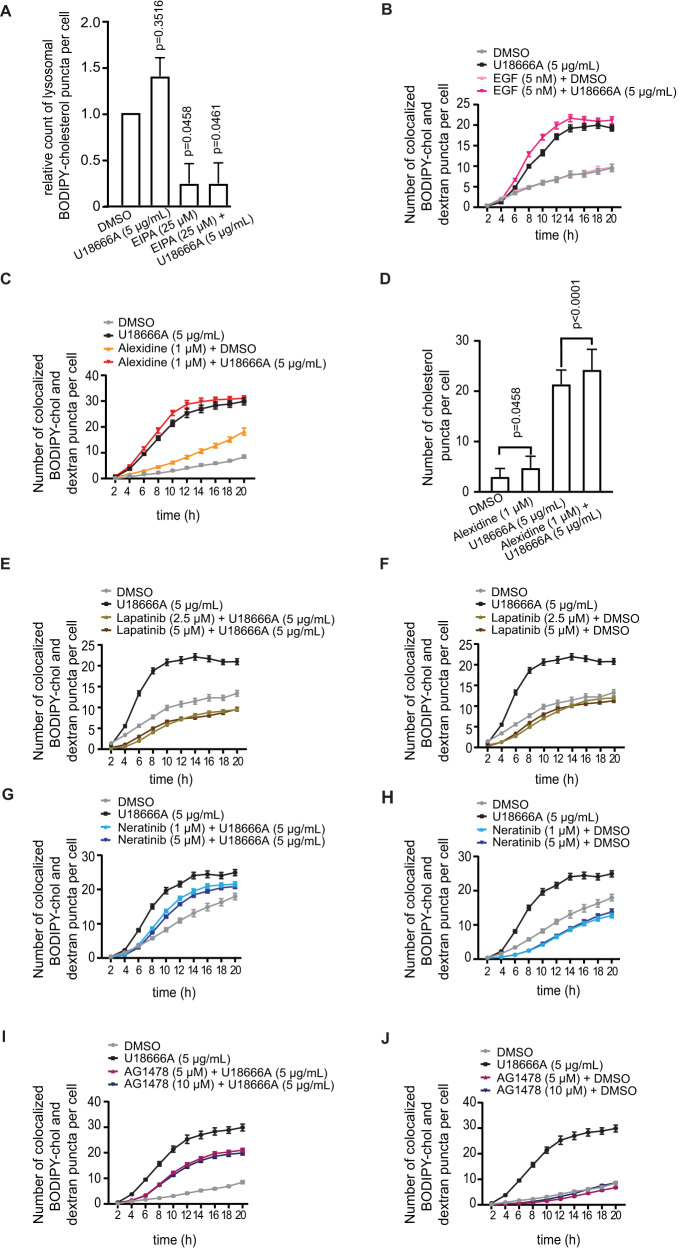
Macropinocytosis increases cholesterol transport. **A** Image-based quantification of BODIPY-cholesterol accumulation in 10 kDa dextran-stained lysosomes of p95ErbB2 cells that were treated with either 25 μM EIPA, 5 μg/ml U18666A, combination of both drugs or with DMSO alone for 6 h. Bars present mean ± SEM of *n* = 3 biological experiments quantified from 9 different images from triplicate wells normalized to the DMSO treated control. **B** Image-based quantification of BODIPY-cholesterol accumulation in 10 kDa dextran-stained lysosomes of p95ErbB2 cells treated with either 5 nM EGF, 5 μg/ml U18666A, their combination or DMSO alone for 20 h with imaging every 2 h. Graphs represent number of BODIPY-cholesterol puncta co-localized with lysosomes per cell blotted as a function of time. Each point is mean ± SEM of 9 images acquired per triplicate condition. **C** Image-based quantification, as in **B**, of p95ErbB2 cells treated with 1 μΜ alexidine, 5 μg/ml U18666A, combination of both drugs or with DMSO alone for 20 h. **D** Quantification of fluorescent images of filipin III-stained free cholesterol accumulation in p95ErbB2 cells after treatment with 1 μM alexidine, 5 μg/ml U18666A, both or with DMSO alone for 24 h. Bars present mean ± SEM of *n* = 3 biological experiments quantified from 9 different images from triplicate wells. **E**–**J** Image-based quantification of BODIPY-cholesterol accumulation in 10 kDa dextran-stained lysosomes of p95ErbB2 cells treated with indicated drugs.

Next, we studied whether macropinocytosis was involved in the cholesterol uptake and could contribute to its transport by using EIPA in the BODIPY-cholesterol uptake assay. Treatment of cells with 25 μM EIPA inhibited BODIPY-cholesterol uptake and its U18666A-dependent accumulation in lysosomes as soon as 6 h after administration (Fig. 4A; in longer timepoints EIPA treatment was cytotoxic). At 6 h timepoint, the effect of U18666A alone was non-significant compared to the DMSO-treated control (Fig. 4A). Activation of macropinocytosis by administration of epidermal growth factor (EGF) increased the lysosomal accumulation of BODIPY-cholesterol (Fig. 4B). Treatment of cells with alexidine, that increases the expression of NPC1 [28] alone or together with U18666A, resulted in an increased accumulation of lysosomal BODIPY-cholesterol (Fig. 4C). Supportively, alexidine increased significantly the filipin III puncta formation (Fig. 4D). Uptake of BODIPY-cholesterol was inhibited by treating cells with lapatinib (Fig. 4E, F) or neratinib (Fig. 4G, H), both of which resulted in the inhibition of EGFR, which is controlled by ErbB2 signaling in these cells (Supplementary Fig. 3B, C). Treatment of cells with lapatinib and neratinib or AG1478 with U18666A (Fig. 4E, G, I) or without (Fig. 4F, H, J) indicated decreased cholesterol uptake and accumulation in comparison to DMSO control and 5 μg/ml of U18666A. Treatment with any of these kinase inhibitors resulted in decreased lysosomal BODIPY-cholesterol puncta in comparison to U18666A treatment alone. These results support the involvement of NPC1 and EGFR-activated macropinocytosis in the increased cholesterol uptake.

### Inhibition of NPC1, ErbB2 and EGFR inhibit invasion but not migration

**Fig. 5:**
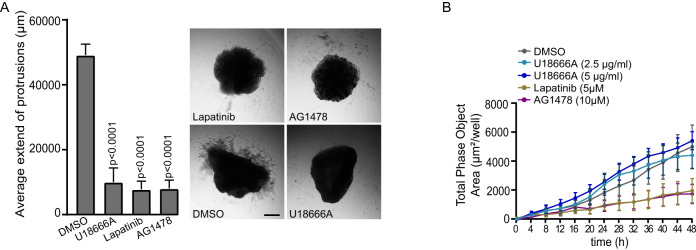
Inhibition of NPC1, ErbB2 and EGFR inhibit invasion but not migration. **A** U18666A, lapatinib and AG1478 treatment of p95ErbB2 spheroids. Quantification of the length of the invasive protrusions at the 48 h time-point of U18666A-, lapatinib- and AG1478-treated spheroids (left). Representative images of their growth in Matrigel (right). Spheroids are imaged 48 h after embedding in Matrigel. Graph bars represent the mean ± SD. Four spheroids from *n* = 3 biological repeats were quantified per treatment. *P* values are calculated by one-way ANOVA. Differences are considered significant at values *p* ≤ 0.05. **B** Chemotaxis migration assay. Cells were treated as indicated and their migration through porous membrane was quantified as appearance of cells capable of migrating through the membrane were quantified as total phase object area from the chemoattractant side of the membrane. Each point is mean ± SEM of 3 biological repeats.

Treatment with NPC1 inhibitor U18666A inhibited the invasion of p95ErbB2 expressing spheroids significantly (Fig. 5A). Similarly, spheroid invasion was inhibited by treatment with ErbB2 and EGFR inhibitors (Fig. 5A). In chemotaxis migration assay the U18666A-treatment did not affect the migration on contrary to lapatinib and AG1478 (Fig. 5B), supporting the observation that NPC1 is specifically regulating invasion.

### NPC1 is needed for invasion and invasion-promoting distribution of lysosomes at the cell periphery in wild type ErbB2 expressing ovarian cancer cells

**Fig. 6:**
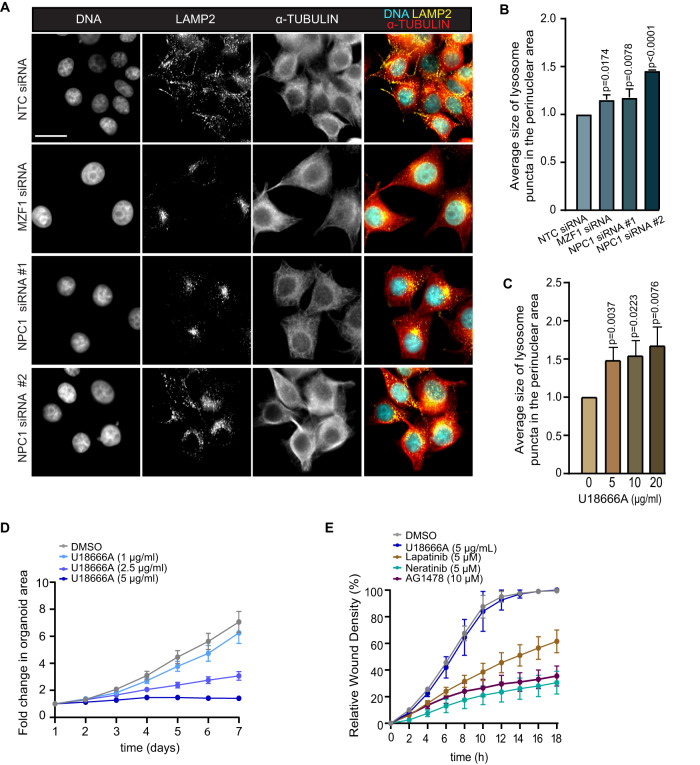
NPC1 is needed for the invasion and invasion-promoting distribution of lysosomes at the cell periphery in wild type ErbB2 expressing ovarian cancer cells. **A** Representative images of lysosomal accumulation at the perinuclear area of MZF1 siRNA, two different NPC1 siRNAs or a NTC siRNA transfected p95ErbB2 cells (72 h). Scale bar is 50 μm. **B** Quantification of perinuclear lysosome distribution in images from **A**. Bars are mean ± SD of *n* = 6–9 images acquired per triplicate of each condition from *n* = 3 independent experiments. **C** Quantification as in **B** of images of p95ErbB2 cells treated with 5 μg/ml of U18666A and DMSO for 24 h. **D** Quantification of images of the invasive growth of Matrigel-embedded ErbB2 positive OVC316 tumor organoids after treatment with 5 μg/ml U18666A and DMSO. Images were taken every 24 h for 7 days following the treatment. *n* = 3 independent experiments. Each point is mean ± SEM of 8 images acquired per triplicate condition. **E** Quantification of a live assay of migration of OVC316 in scratch-wound (wound healing) migration assay. Cells were treated as indicated, and the wound closure was measured over the time. Statistical significances (p) for **B** and **C** were calculated by one-way ANOVA combined with Dunnett’s multiple comparisons. Differences are considered significant at values p ≤ 0.05.

Invasion of p95ErbB2 expressing cells is connected to and correlates with the localization of lysosomes to the invadosome-like structures, which appear as protrusions in the cellular periphery in response to ErbB2 activation [17]. Only when localized to the cellular periphery, lysosomes can contribute to the invasion by releasing their digestive contents to the extracellular space to initiate and promote matrix degradation [29–31]. We investigated weather NPC1 contributed to lysosome-mediated invasion by regulating lysosome distribution. Depletion of either NPC1 or MZF1 by siRNAs lead to the re-positioning of lysosomes from their invasion promoting peripheral position to the perinuclear area (Fig. 6A, B). Similarly, the treatment of cells with U18666A promoted perinuclear distribution of lysosomes (Fig. 6C). Supporting the role of NPC1 in invasion, 5 μM U18666A treatment of ErbB2 positive ovarian cancer organoids, whose invasive growth is dependent on full-length ErbB2 [29], resulted in complete inhibition of invasive growth (Fig. 6D). As for p95ErbB2 cells (Fig. 5B), the migration was not affected by NPC1 inhibition, although it was strongly affected by ErbB2 and EGFR inhibition (Fig. 6E, Supplementary Fig. 3D). Prompted by the important role of cholesterol metabolism and trafficking in the invasive capacity of ErbB2-driven cancer cells, we investigated the effect of p95ErbB2 expression on the entire lipidome by mass spectrometry-based shotgun lipidomics. Unexpectedly, total lipidome analysis showed no significant differences in the total lipidome composition, including cholesterol, with p95ErbB2 expression (Supplementary Fig. 4A), raising the possibility that the uptake of cholesterol gave cells another advantage that increased their invasiveness than mere increase in total cholesterol. These results strongly support for a lysosome-targeting, invasion regulating role for NPC1 and the importance of its ability to regulate extracellular cholesterol uptake and trafficking for invasion.

## Discussion

Tightly regulated cholesterol homeostasis is crucial for cancer development and progression [1]. Cancer cells can overcome poor availability of nutrients by scavenging extracellular proteins and lipids to support their proliferation [32]. Here we identify macropinocytosis as a mechanism that cancer cells can employ for the regulation of cholesterol homeostasis when excess extracellular cholesterol is available. Macropinocytosis is an economical mechanism to fulfill cancer cells need of cholesterol, since it does not require specific receptor-carrier binding, and thus does not need to upregulate the expression of these molecules. Activation of macropinocytosis can make cells capable of bypassing receptor-mediated uptake of extracellular material. One of the well-known activators of macropinocytosis is EGFR [33], which is also one of the most frequently activated receptor tyrosine kinases in cancer [34]. Here we show how activation of EGFR signaling can lead to a coordinated activation of macropinocytosis and cholesterol uptake via enhanced expression of the lysosomal cholesterol transporter NPC1.

The level of NPC1 is a limiting factor for the efficient utilization of extracellular cholesterol. MZF1 is an oncogenic transcription factor that is activated by p95ErbB2 and is positively regulating lysosome-mediated invasion of cancer cells via cathepsin B expression [17, 21]. It is additionally orchestrating ErbB2-induced redistribution of lysosomes to the cellular periphery by the plasma membrane, which allows lysosomes to assist extracellular matrix degradation and invasion [30]. MZF1 is a likely regulator of NPC1 expression. Increased expression of NPC1 allows cells to take in, process and utilize extracellular cholesterol more efficiently. When cells utilize extracellular cholesterol, they are not dependent on cholesterol synthesis and can shut down the expression of cholesterol synthesis genes. Interestingly, increased cholesterol uptake in this case did not lead into higher cholesterol levels in cells as measured using shotgun lipidomics and comparing the cholesterol levels in p95ErbB2 expressing cells to that of the corresponding control cells. The importance of cholesterol homeostasis for the well-being of cancer cells and their ability to tightly control cholesterol metabolism is also indicated by the GOBP analysis showing positive regulation of the cholesterol efflux pathway to coincide with reduced expression of cholesterol synthesis genes. Cholesterol synthesis is an energy-consuming process and the production of one mole of cholesterol requires 36 moles of ATP. When extracellular cholesterol is available and can be taken in as a “by-product” of another cellular process, turning down cholesterol synthesis is a natural consequence. It allows them to use the excess energy for other functions such as migration and invasion, which is likely to occur here. Thus, saving energy and channeling it to other cellular functions is a likely outcome of this metabolic change.

Well-functioning cholesterol metabolism is fundamental for the invasion and metastasis of cancer cells. Cholesterol content of plasma membrane determines its fluidity, number of lipid rafts and is required for membrane ruffling and macropinocytosis [35]. The membrane localization on many signaling proteins requires cholesterol. One of these is Rac 1 whose ability to regulate actin reorganization needed for the growth factor-induced formation of membrane ruffles depends on cholesterol [35] and which is needed for the ErbB2-induced breast cancer cell invasion [36]. We show here that even total cholesterol level remains the same, cells become more invasive and that their increased invasiveness is dependent on NPC1. Another “benefit” for a cancer cell can be that the flow of free cholesterol through lysosomes can change cellular signaling. Recent studies show that lysosomal cholesterol level can regulate the activity of mTORC1 [37], which is activated among others by ErbB2 signaling (Supplementary Fig. 4B). It is often activated in breast cancer and involved in cell migration and invasion [38]. Its activation mechanism requires cholesterol accumulation in lysosomes, where it can recruit mTORC1 to lysosomal surface for activation [39]. Increased transport of extracellular cholesterol via lysosomes may be involved in the activation of mTORC1 signaling. The study was conducted in non-cancerous cell lines, and it indicated that the cholesterol-mediated mTORC1 activation is inversely dependent on NPC1 and its association with cholesterol [39]. That suggests that the fine-tuning of mTORC1 activity via recruitment to cholesterol in cancer cells may vary substantially from that of non-cancerous cells, which could make it a good therapeutical target. Moreover, the fact that mere increase in the expression level of NPC1 results in increased cholesterol uptake, suggests that NPC1 itself may regulate cholesterol uptake and could thus be targeted to some extent.

Cancer cells often activate macropinocytosis to avoid starvation [40, 41]. In this study, we show that cancer cells can additionally utilize macropinocytosis to scavenge significant amounts of biologically usable cholesterol, leading to cellular reprogramming. This reprogramming involves increased expression of the lysosomal cholesterol transporter NPC1, whose activity is needed for the rapid flow of cholesterol through the endocytic system and lysosomes, for it to become biologically useful. The cancer cell´s ability to utilize cholesterol that is taken in “randomly” by macropinocytosis, indicates the enormous flexibility of cancer cells and their endless ability to adapt to metabolic and environmental changes. It also demonstrates well how cancer cells can survive and find new mechanisms to overlive and spread. On the other hand, our studies further suggests that inhibition of cholesterol synthesis in cases where cancer cells are capable for activating macropinocytosis, cannot be an efficient treatment to control their growth, since it could result in an increased cholesterol uptake and invasion.

## Materials and methods

### Tissue culture and cell lines

Tetracycline repressed (TET-off) MCF7 M8-tTAS-pTRE-ΔNErbB2 breast cancer cells and the corresponding control MCF7 M8-tTAS-pTRE-vector cells were cultured and induced to express p95ErbB2 or the corresponding empty vector as before [17, 20]. In experiments p95ErbB2 refers to the TET-off cells where the expression of p95ErbB2 was induced by washing off the tetracycline. Vector control cells were treated the same way. CRISPR-Cas9 MZF1-depleted and CRISPR control p95ErbB2 cells are described elsewhere [21]. ErbB2 positive OVC316 ovarian tumor organoids were prepared from OVC316 ovarian cancer xenografts as described earlier [29].

### Fluorescent dyes, antibodies, siRNAs and inhibitors

List of dyes and antibodies is presented in Supplementary Table 3, siRNAs, primers in Supplementary Table 4 and inhibitors in Supplementary Table 5.

### Additional materials

HDL and LDL/VLDL Quantification Kit (Sigma-Aldrich, MAK045, Munich, Germany); Fetal Bovine serum, lipid depleted (Avantor, S181L-100, Radnor Township, Pennsylvania, USA); human LDL (Sigma-Aldrich, LP2, Munich, Germany); human HDL (Sigma-Aldrich, LP3, Munich, Germany).

### Transfection

Reverse transfection of siRNAs was performed in 6-well plates (Nunc™, Thermo Scientific, Waltham, Massachusetts, USA) with Lipofectamine RNAiMAX (Invitrogen, Waltham, Massachusetts, USA) and plasmids pLVX-NPC1(WT)-FLAG [42] which was a gift from Robert Zoncu (Addgene plasmid #164972; http://n2t.net/addgene:164972; RRID:Addgene_164972) and pLVX(Puro) (TaKaRa, #632164, Tokyo, Japan) with Fugene6 (Promega, Wisconsin,USA) according to the manufacturer’s protocol. For rescue experiment, cells were transfected first with siRNA and day after with plasmid. Cells were re-seeded in imaging plates and fixed for immunostaining 72 h after transfection or harvested for RNA or protein isolation.

### RNA isolation, quantitative RT-PCR, and RNA seq

Total RNA isolation and RT-PCR was performed and analyzed as described elsewhere [21]. The relative expression levels of target genes were normalized to PPIB using “Pfaffl method” [43]. RNA-seq clean reads were aligned to the hg19 UCSC RefSeq (RNA sequences, GRCh37) using Bowtie2 [44]. Read count and TPM values were obtained by transforming mapped transcript reads using RSEM [45]. DEGs were defined as genes with fold change ≥ 1.5 and FDR ≤ 0.05 using DESeq2 [46]. GSEA was carried out and visualized using clusterProfiler and clusterProfiler R [47].

### Chromatin immunoprecipitation

ChIP was performed as described [17] and detailed information of primer selection is described in Supplementary Materials and Methods.

### Imaging of LAMP2/lysosomes

Cells were seeded on ScreenStar 96-well microplate (Greiner, Kremsmünster, Austria) and fixed with 4% formaldehyde (Sigma-Aldrich, Munich, Germany), stained and imaged as described elsewhere [29]. Imaging was performed with ImageXpress Confocal HT.ai (Molecular Devices, San Jose, CA) under 40x magnification using ‘Widefield’ Acquisition Mode at 25 °C. 6–9 sites were scanned per replicate. Quantification of images was performed with MetaXpress^®^ High-Content Image Acquisition and Analysis Software (Molecular Devices, San Jose, CA) [29].

### Imaging of lysosomal free cholesterol

Cells were seeded on ScreenStar 96-well microplate and treated with indicated compounds 24 h later and fixed the day after. Samples were quenched with 50 mM NH_4_Cl at RT for 15 min. Cholesterol staining was performed with 0.1 μg/ml filipin III complex (Sigma-Aldrich, Munich, Germany) diluted in PBS and 10% fetal calf serum at RT for 2 h. Cells were incubated with 2 drops/mL NucRed™ Live 647 ReadyProbes™ Reagent (Invitrogen, Waltham, Massachusetts, USA) in PBS at RT for 15–30 min to stain the nucleus. Detection of cholesterol was performed after washing using the ImageXpress Confocal HT.ai. Nine sites were imaged per replicate. Filipin III was detected at the DAPI channel and NucRed at the Cy5 channel. Image analysis was performed via MetaXpress.

### Macropinocytosis

Day after seeding on Screenstar 96-well plates cells were pre-treated with U18666A or inhibitors for 3 h or EIPA (Sigma Aldrich, A3085, Waltham, Massachusetts, USA) for 1 h at 37 °C. Cells were given dextran, Texas Red, 70000 MW, Lysine Fixable (Thermo Scientific, D1864, Waltham, Massachusetts, USA) with U18666A, EIPA and/or inhibitors and incubated at 37 °C for 2 h and then cleared from dextran by incubation with media with inhibitors for 1 h at 37 °C. Following 6 h treatment with inhibitors or 4 h treatment with EIPA, cells were washed with PBS, fixed with 4% formaldehyde and stained for α-tubulin and DNA. Imaging was performed with ImageXpress Confocal HT.ai. Nine sites were imaged per replicate. Dextran was detected at the Texas Red, α-tubulin at Cy5 and DNA/nucleus at DAPI channel. Image analysis was performed via MetaXpress.

### Imaging of BODIPY-cholesterol

Cells were seeded in 96-well Screenstar plates. Next day, cells were incubated with 150 μg/mL dextran, Alexa Fluor™ 594; 10000 MW, Anionic, Fixable (Invitrogen, D22913, Waltham, Massachusetts, USA) for 20 h, to chase it into the lysosomes/late endosomes [48]. Remaining dextran was cleared with fresh media for 2 h. Cells were treated with 0.2 μM TopFluor® Cholesterol 23-(dipyrrometheneboron difluoride)-24-norcholesterol (BODIPY-cholesterol; Avanti Polar Lipids, Alabaster, Alabama), 0.3 μM Nuclear Violet LCS1 (Nuclear Violet, AAT Bioquest, Pleasanton, CA, USA), 0.2 μM Tubulin Tracker Deep Red (Invitrogen, Waltham, Massachusetts, USA) and inhibitors combined with 5 μg/mL U18666A or DMSO for 2 h. Cells were live-imaged every 2 h for 20 h with ImageXpress Confocal HT.ai at 37 °C and 5% CO_2_. Nine sites were imaged per replicate. Dextran was detected at Texas Red, BODIPY-cholesterol at FITC, a-tubulin at Cy5 and DNA/nucleus at DAPI channel. Image analysis was performed with MetaXpress.

### Immunoblotting

Cells were harvested, lysed, blotted and imaged as described before [29].

### 3D Matrigel Invasion assay for spheroids and organoids

Matrigel 3D invasion assays [17], invasive organoids assay and isolation of OVC316 tumor organoids are presented elsewhere [29].

### Transmembrane invasion and chemotaxis migration assays

Chemotaxis migration assay was performed according to “Incucyte Chemotaxis Cell Migration Assay” protocol (Sartorius). 1000 cells/well were seeded in Incucyte Clearview 96-well chemotaxis plate (Sartorius, 4582, Goettingen, Germany) in 60 µl culture media with 1% FBS and indicated drugs. Media with 10% FBS and 5 nM EGF were used as a chemoattractant. The plate was imaged in the IncuCyte ZOOM live analysis system (Sartorius, Goettingen, Germany) and analyzed with Incucyte Chemotaxis Analysis Software Module (Sartorius, Goettingen, Germany). For quantification the total phase object area of the bottom of the membrane was used and normalized to the area measured at timepoint 0. The transmembrane invasion assay was performed according to “Incucyte Chemotaxis Cell Invasion Assay” (Sartorius). 2000 cells embedded in 20 µl Cultrex Reduced Growth Factor Basement Membrane Extract, type 2, Pathclear (BME-2; Amsbio/R&D Systems, #3533-010-02, Minneapolis, USA) were seeded per well in an Incucyte Clearview 96-well chemotaxis plate. The matrix embedded cells were added in 40 µl culture media with 1% FBS together with indicated drugs. 200 µl media with 10% FBS and 5 nM EGF were added to the reservoir plate as a chemoattractant. The plate was imaged, analyzed and quantified as the chemotaxis migration assay above.

### Scratch-wound migration assay

OVC316 cells (25 × 10^5^) were seeded into an Incucyte® Imagelock 96-well plate (Essen Bioscience, 4379, Hertfordshire, UK) in medium with 10% FBS. Cells were wounded after 48 h incubation with Incucyte® 96-well WoundMaker Tool (Sartorius, 4563, Goettingen, Germany). Each well was washed with 200 µl PBS, after which 200 µl of the culture medium with indicated drugs were added. The plate was pre-incubated 30 min and imaged as indicated. The images were analyzed using the Incucyte® ZOOM software.

### Shotgun lipidomics and Immunofluorescence analysis by quantitative image-based cytometry (QIBC)

Shotgun lipidomics [49] utilizing LipidXplorer [50] and QIBC are presented in detail in Supplementary Materials and Methods.

### Quantification and statistical analysis

Experiments were repeated trice. Statistical analyses of RNA-seq were carried out using R software v4.2.0. Tests of statistical significance were performed using nonpaired two-tailored Student’s *t*-test on an assumption of normal distribution or one-way ANOVA with Dunnett’s correction. Differences were considered significant at values less than 0.05. Majority of quantified data is presented as chart bars, where the height of the bar represents the mean, and the error bar represents the ±SD or ±SEM using GraphPad Prism 5.9.1.

## Supplementary information

Supplementary figures and supplementary materials and methods

Supplementary Table 1

Supplementary Table 2

Supplementary Table 3

Supplementary Table 4

Supplementary Table 5

## Data Availability

The RNA-seq dataset is available at: CNGBdb (https://db.cngb.org/search/project/CNP0003166/).
