# Supplementary material for: Activation of invasion by oncogenic reprogramming of cholesterol metabolism via increased NPC1 expression and macropinocytosis
Source: Oncogene. 2023 Jul 7;42(33):2495–506. doi: 10.1038/s41388-023-02771-x (PMC10421736; doi:10.1038/s41388-023-02771-x)
Supplement: Supplementary file 1 — Supplementary figures and supplementary materials and methods [file 41388_2023_2771_MOESM1_ESM.docx]

**Skorda et al.2022;**

**Supplementary materials and methods and supplemental figures and figure texts**

|  |
| --- |
|  |

**Supplementary Materials and Methods:**

RNA-seq samples

For RNA-seq total RNA was isolated as three biological repeats 3 passages after induction from TET-off p95ErbB2 cells, corresponding control vector expressing cells and two clones of CIRSPR-Cas9 p95ErbB2 cells with two different MZF1 indels, MZF1-13 and MZF1-30, and a corresponding control cell line with no MZF1 indel, MZF1-12. Establishment of the CRISPR-Cas9 cell lines has been published before (*1*). The RNA-seq results of the three biological repeats are available in: <https://db.cngb.org/search/project/CNP0003166/> . Here the samples are named as: Vect-1,2,3; deltaN-1,2,3 (p95ErbB2); C-12-1,2,3; C-13-1,2,3, and C-30-1,2,3.

Shotgun lipidomics

-Materials:

Chemicals, solvents, and synthetic lipid standards were purchased from Sigma-Aldrich (St. Louis, MO), Rathburn Chemicals (Walkerburn, Scotland), Avanti Polar Lipids (Alabaster, AL), Larodan AB (Solna, Sweden).

-Methods:

Lipid Extraction: Aliquots of 200,000 mammalian cells were subjected to lipid extraction as described in (*2*) piked with internal lipid standard mixture containing: 31.3 pmol cardiolipin (CL), 27.8 pmol cholesteryl ester (CE) 15:0-D_7_, 25 pmol ceramide (Cer) 18:1;2/12:0;0, 306.6 pmol cholesterol (Chol)-D_4_, 12.5 pmol diacylglycerol (DAG) 12:0/12:0, 15.4 pmol dihexosylceramide (diHexCer) 18:1;2/17:0;0, 31.3 pmol hexosylceramide (HexCer) 18:1;2/12:0;0, 27.5 pmol lysophosphatidic acid (LPA) 17:0, 25 pmol lysophosphatidylcholine (LPC) 12:0, 27.1 pmol lysophosphatidylethanolamine (LPE) 13:0, 16.3 pmol lysophosphatidylglycerol (LPG) 17:1, 17.6 pmol lysophosphatidylinositol (LPI) 13:0, 23 pmol lysophosphatidylserine (LPS) 17:1, 25.5 pmol phosphatidic acid (PA) 12:0/12:0, 25 pmol phosphatidylcholine (PC) 12:0/12:0, 31.3 pmol phosphatidylethanolamine (PE) 12:0/12:0, 17.2 pmol phosphatidylglycerol (PG) 12:0/12:0, 17.1 pmol phosphatidylinositol (PI) 8:0/8:0, 10 pmol phosphatidylserine (PS) 12:0/12:0, 21.3 pmol sphingomyelin (SM) 18:1;2/12:0;0, 18.8 pmol trihexosylceramide (triHexCer) 18:1;2/17:0;0.

-Mass spectrometric lipid analysis:

Quantitative shotgun lipidomics were performed using a Orbitrap Fusion Tribrid mass spectrometer (Orbitrap Fusion, Thermo Fisher Scientific) equipped with a TriVersa NanoMate (Advion Biosciences, Ithaca, NY, USA). All data were recorded using Fourier transform mass spectrometry (FT MS) and FT MS/MS scans in the positive and negative ionization modes with optimal settings. Then, the data were transferred to the LipidXplorer software (*3*) for lipid identification and further processed for quantification using the homemade software LipidQ software (<https://github.com/ELELAB/lipidQ>) (*2*).

-Annotation of Lipid Species:

The GL and (lyso)GPL species are annotated according to their sum composition: <lipid class> <total number of carbon atoms in fatty acid moieties>:<total number of double bonds in fatty acid moieties> (e.g., PC 34:1). The SL species are also annotated according to their sum composition: <lipid class> <total number of carbon atoms in the long-chain base and fatty acid moiety>:<total number of double bonds in the long-chain base and fatty acid moiety>;<total number of OH groups in the long-chain base and fatty acid moiety> (e.g., HexCer 34:1;2).

Primer selection for quantitative Reverse Transcription qPCR and ChIP

Primer-BLAST from National Center for Biotechnology Information (NCBI) was used to design primer sequences. Primers were designed so that PCR products were as close as possible of 200 bp, making them suitable for quantitative PCR. NPC1 and PPIB primers for RNA quantification were planned to be intron spanning. Primers for NPC1 ChIP were planned to span sequences between -880- -698 from the transcription start site (TSS) that contains one putative MZF1 binding site. Four additional putative sites were found upstream and within 2000 bp region (minimum 98% confidentiality). Upstream promoter region of LIN28A (-4394- -4155 of TSS) that contains no putative NPC1 binding sites was used as a potential negative control.

Immunofluorescence analysis by quantitative image-based cytometry (QIBC)

Cells were treated with siRNAs and the next day seeded into Screenstar microplates (Greiner). 48 hours later cells were fixed with 4% formalin, permeabilized with 0.5% Triton X100, and stained with primary antibodies in 2%FBS containing PBS overnight at 4 degrees Celsius. Cells were washed 3x in 250µL PBS and nuclear staining (DAPI) as well as species-specific secondary antibodies (Alexa Fluor 488 and Alexa Fluor 647) were applied for 1 hour at room temperature in the dark. After 3 washes with 250µL PBS, cells were mounted with Flouromount (Southern Biotech) and imaged using the Olympus ScanR High-Content Screening Station at 20x magnification with pictures taken at 49 different viewing fields in each well. Automated image analysis was performed with the Olympus ScanR Image Analysis software. Nuclei were detected using the neural network-driven nuclear object mask and the cytoplasm was assessed in 10 pixels around the nuclear mask. Single cells were detected via population gating using circularity factor and nuclear area parameters as well as mean and total DAPI fluorescence intensities. Scatter plots for mean fluorescence of cytoplasmic proteins were generated using TIPCO Spotfire. A mean with SEM of the median fluorescence of 3 independent biological replicates was used to visualize differences in single protein staining in GraphPad Prism 9. Statistical significance between siRNA-treated samples was assessed by paired, 2-tailed t-test.

**Supplementary Figures and Figure texts:**

**
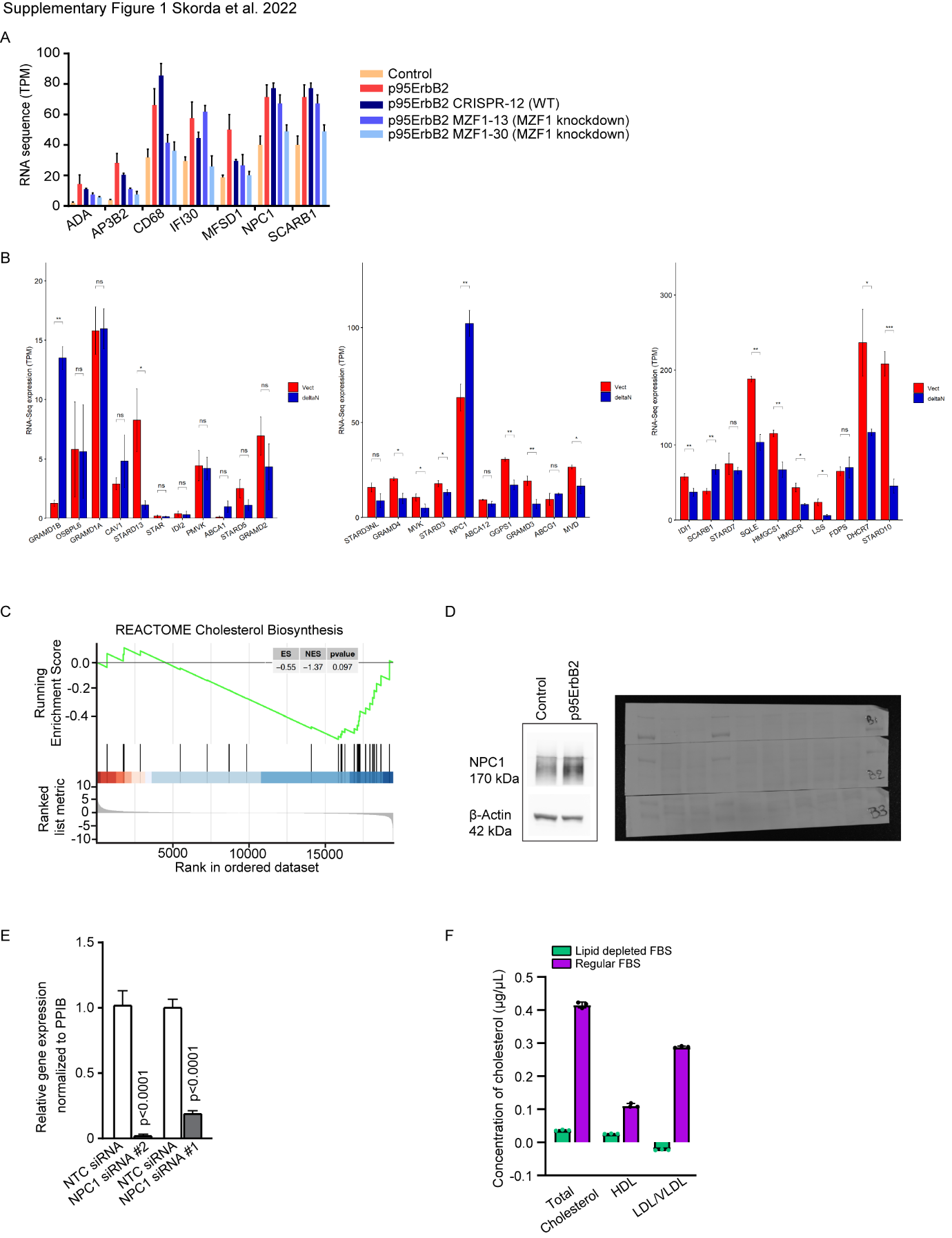
**

**Supplementary Figure 1.** **Lysosomal and cholesterol metabolism genes regulated by p95ErbB2 expression. A** RNA-seq results presented as transcripts per million (TPM) of lysosomal mRNAs that are upregulated by ErbB2 (red; wild type and black; negative CRISPR control with wild type MZF1 (CRISPR-12) ) and potentially downregulated by MZF1 depletion (dark blue (MZF1-13) and light blue (MZF1-30) for two MZF1 CRISPR clones with different indels and the empty vector control (orange) a respective control for the p95ErbB2 cells (*1*)). The graph is prepared from the RNA-seq results of three biological repeats available in: <https://db.cngb.org/search/project/CNP0003166/>. **B** Graphs of the expression of the selected cholesterol synthesis regulating mRNAs prepared from the RNA-seq results. Column diagrams are divided into three groups based on the expression levels of the mRNAs (low, medium, high). NPC1 and SCARB1 mRNAs are added as references in medium and high expression level graphs for the evaluation of their expression levels in comparison to the expression levels of the cholesterol synthesis genes. Statistical analyses were carried out using R software v4.2.0. **C** Gene set enrichment analysis (GSEA) of cell lines comparing p95ErbB2 and control cell lines. Enrichment plot of the REACTOME_CHOLESTEROL_BIOSYNTHESIS pathway including genes: ACAT2, ARV1, CYP51A1, DHCR24, DHCR7, EBP, FDFT1, FDPS, GGPS1, HMGCR, HMGCS1, HSD17B7, IDI1, IDI2, LBR, LSS, MSMO1, MVD, MVK, NSDHL, PLPP6, PMVK, SC5D, SQLE, TM7SF2. Analysis was done according to kallisto-method (*4*) **D** Immunoblot and membrane of the immunoblot of the Figure 1C. **E** Efficiency of the NPC1 siRNAs upon 72 h transfection measured by quantitative real time PCR. Expressions of downregulated NPC1 mRNAs were compared to their expression in NTC siRNA transfected cells. NPC1 expressions were normalized to the expression of PPIB gene. **F** LDL and HDL concentration measurement in the culture medium serums that are used in Figs 1E and F. This information was used to adjust the LDL and HDL concentrations in lipid-depleted serum to match that of the regular FBS for the rescue experiments in the Fig. 1F. Measurement was done according to the instructions of the HDL and LDL/VLDL Quantification Kit, Sigma-Aldrich, MAK045.


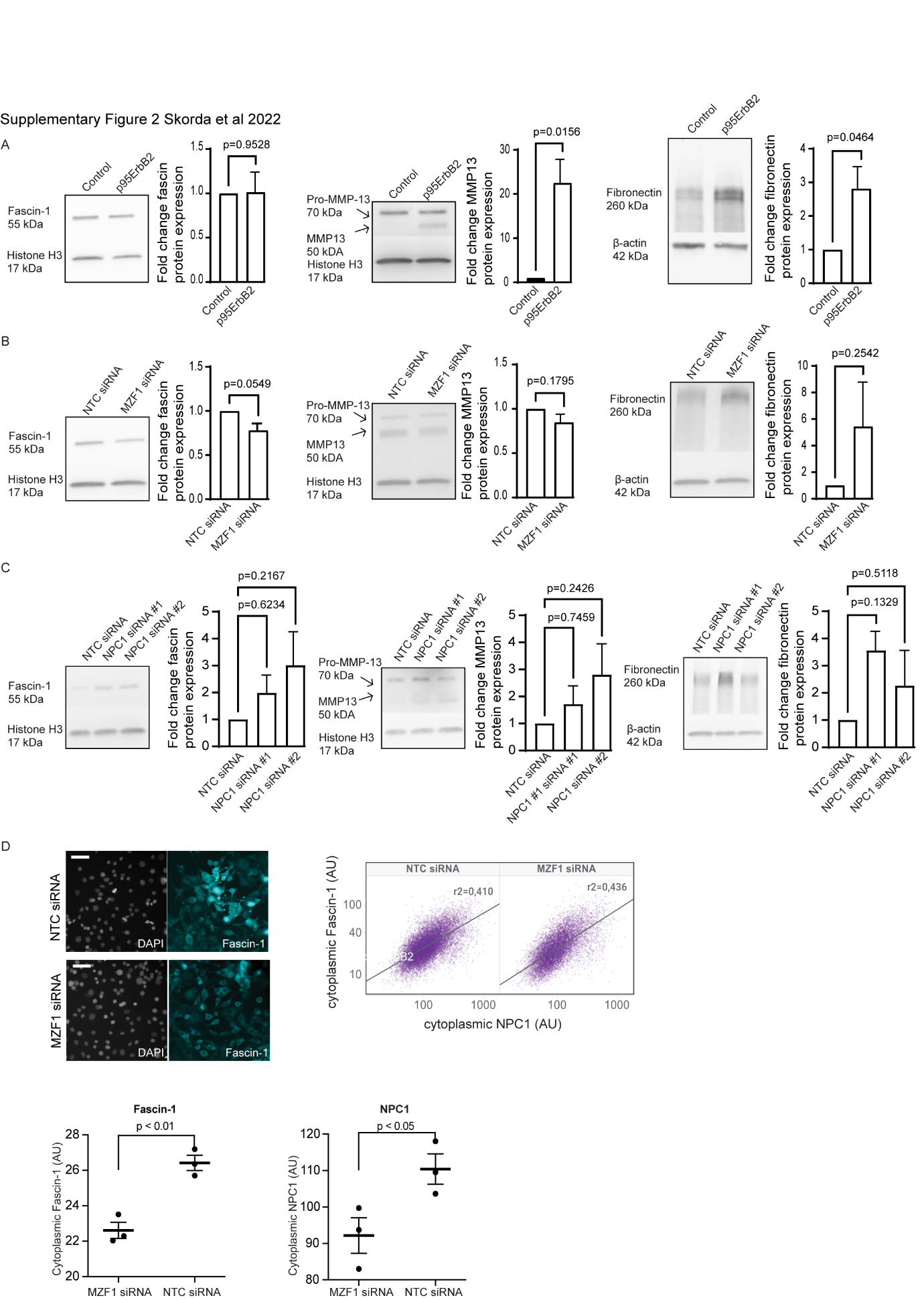


**Supplementary Figure 2. Invasion markers measured in control and p95ErbB2 cells. A-C** Immunoblots of three invasion markers: Fascin-1, MMP-13 and fibronectin. Protein expression is shown with a representative blot and quantification. **A** Western blot of the three invasion markers measured in control and p95ErbB2 cells. Quantification of three repeats is shown normalized to the loading control and as fold change in relation to the control cells. **B** Western blot of the three invasion markers measured in p95ErbB2 cells treated with either NTC siRNA or MZF1 siRNA for 72 hours. Quantification of three repeats is shown normalized to the loading control and as fold change in relation to the NTC siRNA. **C** Western blot of the three invasion markers measured in p95ErbB2 cells treated with either NTC siRNA, NPC1 siRNA #1 or NPC1 siRNA #2 for 72 hours. Quantification of three repeats is shown normalized to the loading control and as fold change in relation to the NTC siRNA. **D** Immunofluorescence analysis by quantitative image-based cytometry (QIBC). Representative images and analysis showing the correlation of cytoplasmic fascin-1/NPC (upper left). Scale bar is 50 μm. Mean fluorescence is measured 10 pixels surrounding the nuclear mask (upper right). The straight line and R2 of regression show correlation of the indicated proteins. Depicted are cumulates of 3 biological repeats. AU=arbitrary units of mean fluorescence. Individual median fluorescence of indicated cytoplasmic proteins (low). Fluorescence is measured 10 pixels surrounding the nuclear mask. Shown is the mean of 3 biological repeats (indicated as dots) with SEM. Statistical significance was determined by paired t-test of biological replicates. AU=arbitrary units of mean fluorescence.

**
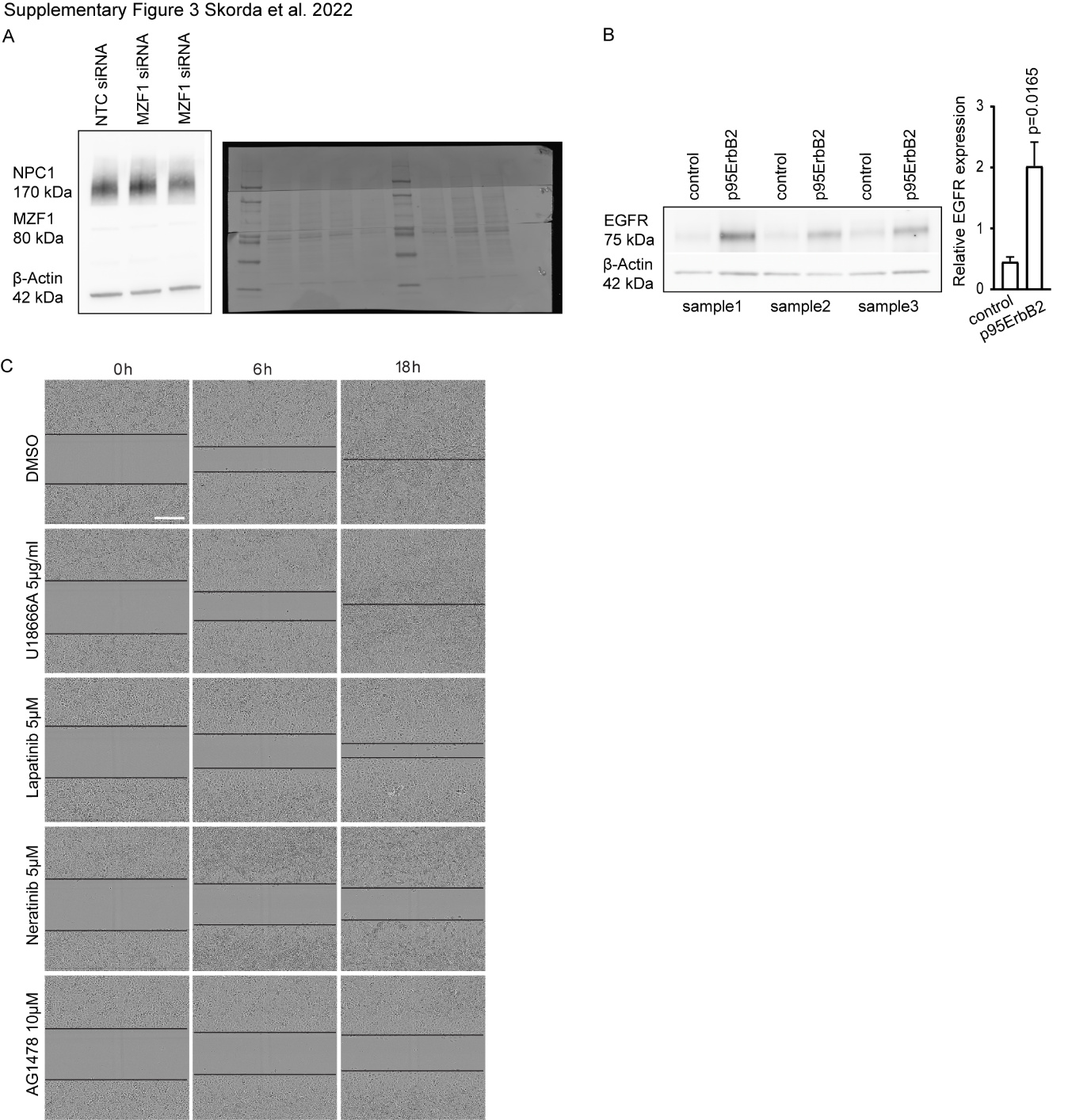
**

**Supplementary Figure 3.** **MZF1 regulation of the expression of NPC1.** **A** Membrane of the immunoblot and the repeats of the immunoblot of the Figure 2F. **B** p95ErbB2 increases expression of EGFR. Immunoblot of EGFR expression in response to p95ErbB2 expression. Quantification of EGFR expression normalized to the expression of beta-actin. n=3. **C** Representative images of wound healing migration assay presented in Figure 6E. Scale bar is 300 μm.


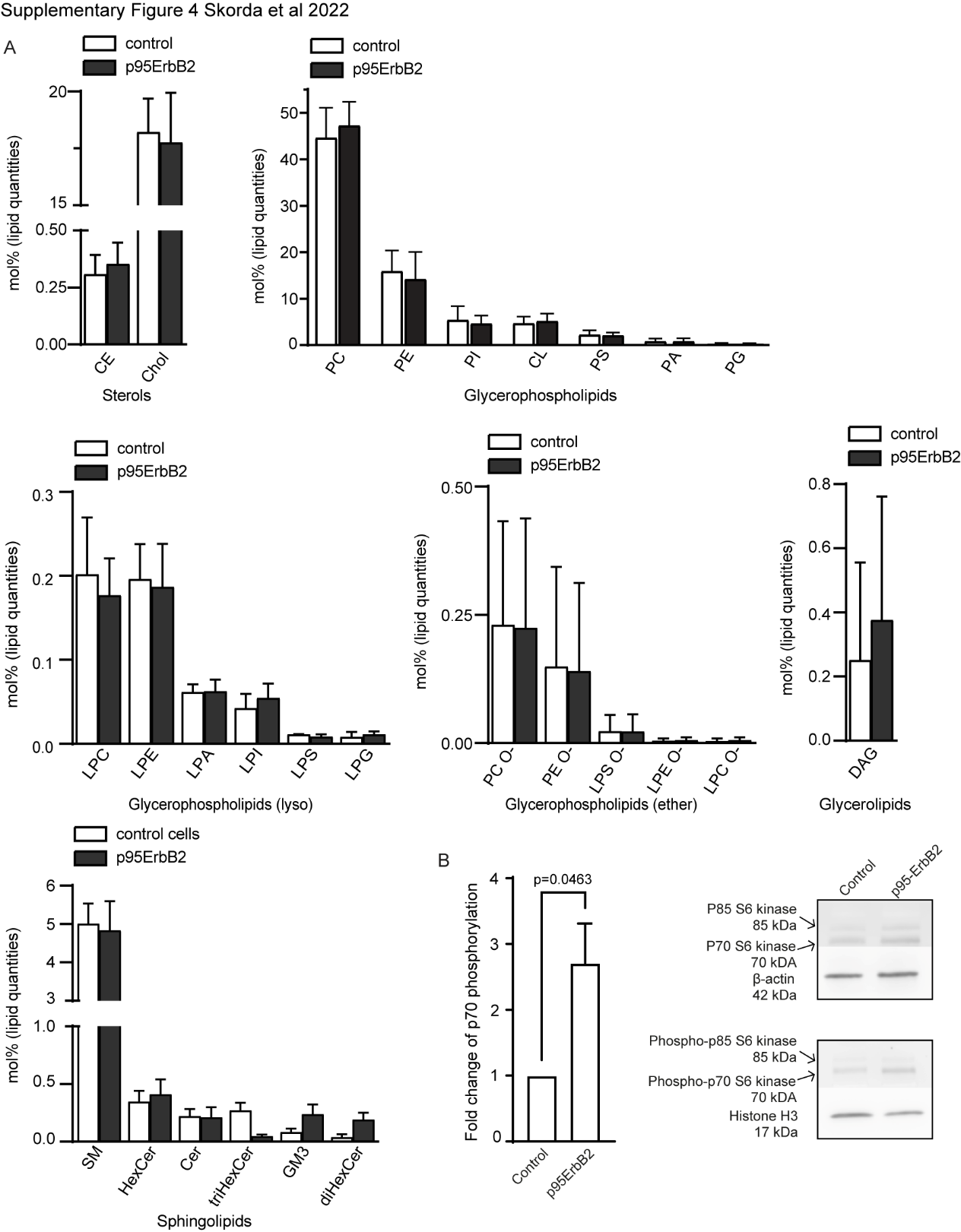


**Supplementary Figure 4. Expression of p95ErbB2 does not induce change in the intracellular lipid concentrations or compositions.** Pooled three biological repeats of shotgun lipidomics experiment of p95ErbB2 and corresponding control vector expressing cells. Quantitative shotgun lipidomics profiling of lipids classes within the p95ErbB2 and the corresponding control vector expressing cells. The lipidome profiling covers 27 classes from 380 quantified lipid species. Lipid quantities are reported as mol%. Lipid classes are abbreviated as: bis(monoacylglycero)phosphate (BMP), cholesteryl ester (CE), ceramide (Cer), ceramide phosphate (CerP) cholesterol (Chol), diacylglycerol (DAG), dihexosylceramide (diHexCer), hexosylceramide (HexCer), long-chain base (LCB), long-chain base phosphate (LCBP) , lysophosphatidic acid (LPA), lysophosphatidylcholine (LPC), lysophosphatidylethanolamine (LPE), lysophosphatidylglycerol (LPG), lysophosphatidylinositol (LPI), lysophosphatidylserine (LPS), lysosphingomyelin (LSM), phosphatidic acid (PA), phosphatidylcholine (PC), phosphatidylethanolamine (PE), phosphatidylglycerol (PG), phosphatidylinositol (PI), phosphatidylserine (PS), sphingomyelin (SM), sulfatide (SHexCer), triacylglycerol (TAG), trihexosylceramide (triHexCer). Glycerophospholipids with O-, for example PC O-, are the ether versions. **B** Immunoblot of mTOR activation quantified from the phosphorylation of P70 S6 kinase. Protein expression of P70 S6 kinase and phosphorylated P70 S6 kinase measured with immunoblot in control and p95ErbB2 cells shown with representative blots and quantification. Quantification is performed by normalizing the protein expression to the loading controls and calculating the relative amount of p70 S6 kinase, which had been phosphorylated. Bars represent mean ± SEM of n=3 independent experiments shown as fold change in relation to the control.

**Supplementary References:**

1. D. M. Brix, S. A. Tvingsholm, M. B. Hansen, K. B. Clemmensen, T. Ohman, V. Siino, M. Lambrughi, K. Hansen, P. Puustinen, I. Gromova, P. James, E. Papaleo, M. Varjosalo, J. Moreira, M. Jaattela, T. Kallunki, Release of transcriptional repression via ErbB2-induced, SUMO-directed phosphorylation of myeloid zinc finger-1 serine 27 activates lysosome redistribution and invasion. *Oncogene* **38**, 3170-3184 (2019).

2. I. O. Nielsen, A. Vidas Olsen, J. Dicroce-Giacobini, E. Papaleo, K. K. Andersen, M. Jaattela, K. Maeda, M. Bilgin, Comprehensive Evaluation of a Quantitative Shotgun Lipidomics Platform for Mammalian Sample Analysis on a High-Resolution Mass Spectrometer. *J Am Soc Mass Spectrom* **31**, 894-907 (2020).

3. R. Herzog, K. Schuhmann, D. Schwudke, J. L. Sampaio, S. R. Bornstein, M. Schroeder, A. Shevchenko, LipidXplorer: a software for consensual cross-platform lipidomics. *PLoS One* **7**, e29851 (2012).

4. N. L. Bray, H. Pimentel, P. Melsted, L. Pachter, Near-optimal probabilistic RNA-seq quantification. *Nat Biotechnol* **34**, 525-527 (2016).
