# Supplementary material for: Activation of invasion by oncogenic reprogramming of cholesterol metabolism via increased NPC1 expression and macropinocytosis
Source: Oncogene. 2023 Jul 7;42(33):2495–506. doi: 10.1038/s41388-023-02771-x (PMC10421736; doi:10.1038/s41388-023-02771-x)
Supplement: Supplementary file 3 — Supplementary Table 2 [file 41388_2023_2771_MOESM3_ESM.pdf]

Supplementary Table 2: GSEA pathway analysis with p-value <0.05 of the RNA sequence comparison of SVect vs. p95ErbB2.

ID	Description	setSize	enrichmentScore	NES	pvalue	p.adjust	qvalue	core_enrichment
R-HSA-5334118	DNA methylation	63	-0.737362698	-2.684268669	8.73E-13	8.16E-10	6.96E-10	8347/3021/8362/8341/333932/126961/8370/554313/8339/8355/8334/3014/8367/121504/653604/8368/8348/8364/55766/8335/8968/8366/3017/474382/8343/1789/8344/8354/3012/8360/8351/8365/94239/3015/8346/8358/8350/8357/8342/8331/8340/8345/8361/8356/8359/29947/8294/8352
R-HSA-73728	RNA Polymerase I Promoter Opening	61	-0.734204782	-2.639044883	6.69E-13	8.16E-10	6.96E-10	7343/3020/8338/5595/8347/3021/8362/8341/333932/126961/8370/554313/8339/8355/8334/3014/8367/121504/653604/8368/8348/8364/55766/8335/8968/8366/3017/474382/8343/8344/8354/3012/8360/8351/8365/94239/3015/8346/8358/8350/8357/8342/8331/8340/8345/8361/8356/8359/8294/8352
R-HSA-68616	Assembly of the ORC complex at the origin of replication	67	-0.715849053	-2.604331594	2.70E-12	1.01E-09	8.62E-10	8363/23594/8353/5001/3020/8338/5000/8347/3021/8362/8341/333932/126961/8370/554313/8339/8355/8334/3014/8367/121504/653604/8368/8348/8364/55766/8335/8968/8366/3017/474382/8343/8344/8354/3012/8360/8351/8365/94239/3015/8346/8358/8350/8357/8342/8331/8340/8345/8361/8356/8359/8294/8352
R-HSA-212300	PRC2 methylates histones and DNA	71	-0.703584384	-2.576010306	2.37E-12	1.01E-09	8.62E-10	8347/3021/8362/8341/333932/126961/8370/554313/8339/8355/8334/3014/8367/121504/653604/8368/8348/8364/55766/8335/8968/8366/3017/474382/8343/1789/8344/8354/3012/8360/8351/8365/94239/3015/8346/8358/8350/8357/8342/8331/8340/8345/8361/8356/8359/8294/8352
R-HSA-1500620	Meiosis	113	-0.627081035	-2.456006004	1.98E-12	1.01E-09	8.62E-10	10274/26277/8363/4683/8353/6119/25777/5888/641/3020/4361/8338/8347/3021/11083/5885/8362/8341/675/333932/126961/8370/554313/8339/8355/8334/3014/8367/8290/121504/653604/8368/8348/256126/8364/55766/8335/8968/8366/50511/4001/3017/474382/8343/8344/8354/10388/3012/8360/8351/10734/8365/94239/3015/8346/8358/8350/8357/8342/23345/8331/8340/8345/23224/8361/8356/8359/8294/8468/8352
R-HSA-2299718	Condensation of Prophase Chromosomes	72	-0.695525878	-2.554608517	3.60E-12	1.12E-09	9.58E-10	8347/3021/8362/8341/333932/126961/8370/554313/8339/8355/8334/3014/8367/8290/121504/653604/8368/8348/8364/55766/8335/8968/8366/3017/10592/474382/8343/8344/8354/3012/8360/8351/8365/94239/3015/8346/8358/8350/8357/8342/8331/8340/8345/8361/8356/8359/8294/8352
R-HSA-427389	ERCC6 (CSB) and EHMT2 (G9a) positively regulate rRNA expression	74	-0.672181564	-2.510072513	2.27E-11	5.30E-09	4.52E-09	8347/3021/8362/8341/333932/126961/8370/554313/8339/8355/8334/3014/8367/121504/653604/8368/8348/8364/55766/8335/8968/8366/3017/474382/8343/8344/8354/3012/8360/8351/8365/94239/3015/8346/8358/8350/8357/8342/8331/8340/8345/8361/8356/8359/8294/8352
R-HSA-5578749	Transcriptional regulation by small RNAs	104	-0.616234048	-2.381028535	2.01E-11	5.30E-09	4.52E-09	8347/3021/8362/8341/333932/126961/8370/554313/8339/8355/8334/3014/8367/121504/653604/8368/8348/8364/55766/8335/8968/8366/3017/474382/8343/8344/8354/3012/8360/8351/8365/94239/3015/8346/8358/8350/8357/8342/8331/8340/8345/8361/8356/8359/8294/8352
R-HSA-427359	SIRT1 negatively regulates rRNA expression	66	-0.705688129	-2.563961129	2.70E-11	5.58E-09	4.76E-09	3020/8338/8347/3021/8362/8341/333932/126961/8370/554313/8339/8355/6839/8334/3014/8367/121504/653604/8368/8348/8364/55766/8335/8968/8366/3017/474382/8343/8344/8354/3012/8360/8351/8365/94239/3015/8346/8358/8350/8357/8342/8331/8340/8345/8361/8356/8359/8294/8352
R-HSA-9710421	Defective pyroptosis	71	-0.685107426	-2.508361227	2.98E-11	5.58E-09	4.76E-09	8347/3021/8362/8341/333932/126961/8370/554313/8339/8355/8334/3014/8367/121504/653604/8368/8348/8364/55766/8335/8968/8366/3017/474382/8343/1789/8344/8354/3012/8360/8351/8365/94239/3015/8346/8358/8350/8357/8342/8331/8340/8345/8361/8356/8359/8294/8352
R-HSA-912446	Meiotic recombination	83	-0.659950423	-2.483532658	3.65E-11	6.20E-09	5.29E-09	8363/4683/8353/6119/5888/641/3020/4361/8338/8347/3021/8362/8341/675/333932/126961/8370/554313/8339/8355/8334/3014/8367/8290/121504/653604/8368/8348/8364/55766/8335/8968/8366/3017/474382/8343/8344/8354/3012/8360/8351/8365/94239/3015/8346/8358/8350/8357/8342/8331/8340/8345/8361/8356/8359/8294/8352
R-HSA-3214847	HATs acetylate histones	140	-0.551160631	-2.200909535	5.70E-11	8.88E-09	7.58E-09	6871/8338/55167/55929/8347/440689/8362/317772/8341/333932/126961/8370/554313/8330/8339/8355/10499/8332/8334/8367/8336/121504/653604/8368/57325/54556/8348/8364/8850/8335/8968/23522/8366/8969/3017/8343/8344/8354/3012/8360/8351/8365/8329/8346/85235/8358/8350/8357/8342/8331/8340/8345/8361/8356/8359/8294/8352

R-HSA-1912422	Pre-NOTCH Expression and Processing	107	-0.606602003	-2.333702523	6.52E-11	9.37E-09	8.00E-09	8347/3021/8362/8341/333932/126961/8370/554313/8339/8355/8334/3014/8367/121504/6487/653604/8368/8348/1869/8364/8850/55766/8335/8968/8366/3017/474382/8343/8344/8354/3012/8360/8351/8365/94239/3015/3955/8346/8358/8350/8357/8342/8331/8340/8345/489/8361/8356/8359/4855/8294/8352/84441
R-HSA-9018519	Estrogen-dependent gene expression	148	-0.55138263	-2.212507173	7.11E-11	9.50E-09	8.10E-09	8347/51523/3021/5885/8362/1509/8341/57678/333932/126961/8370/554313/8339/8355/10499/8334/3014/8367/121504/653604/8368/8348/8364/8850/55766/8335/8968/8366/3017/474382/8343/8344/8354/3012/8360/8351/8365/94239/3015/8346/8358/7031/8350/8357/8342/8331/8340/8345/2625/8361/8356/4602/200845/8359/8294/9687/7033/2099/8352/2494/596/6387/2066
R-HSA-3214815	HDACs deacetylate histones	92	-0.635954262	-2.440307522	7.74E-11	9.65E-09	8.24E-09	8338/6907/8347/440689/79685/5926/8362/317772/9611/8341/333932/126961/8370/554313/8330/8339/8355/8332/8334/8367/8336/121504/653604/8368/8348/8364/8335/8968/8366/8969/3017/8343/8344/8354/3012/8360/8351/8365/8329/8346/85235/8358/8350/8357/8342/8331/8340/8345/8361/8356/8359/8294/8352
R-HSA-8936459	RUNX1 regulates genes involved in megakaryocyte differentiation and platelet function	93	-0.621357559	-2.39150409	9.58E-11	1.12E-08	9.55E-09	23067/9070/8970/23112/57690/58508/2811/55904/8363/8353/3065/3020/8338/3674/7057/8347/3021/8362/8341/333932/126961/8370/554313/8339/8355/8334/3014/8367/121504/653604/8368/8348/8364/8850/55766/8335/8968/8366/3017/474382/8343/8344/8354/3012/8360/8351/8365/94239/3015/8346/8358/8350/8357/8342/8331/8340/8345/8361/8356/8359/8294/8352
R-HSA-1221632	Meiotic synapsis	75	-0.664144722	-2.482615312	1.86E-10	2.05E-08	1.75E-08	8347/11083/5885/8362/8341/8370/554313/8339/8334/3014/8367/8290/121504/8368/8348/256126/8364/55766/8335/8366/50511/4001/3017/474382/8343/8344/10388/3012/8360/10734/8365/94239/3015/8346/8342/23345/8331/8340/8345/23224/8361/8359/8294/8468
R-HSA-1912408	Pre-NOTCH Transcription and Translation	91	-0.622512447	-2.382137201	2.29E-10	2.38E-08	2.03E-08	8347/3021/8362/8341/333932/126961/8370/554313/8339/8355/8334/3014/8367/121504/653604/8368/8348/1869/8364/8850/55766/8335/8968/8366/3017/474382/8343/8344/8354/3012/8360/8351/8365/94239/3015/8346/8358/8350/8357/8342/8331/8340/8345/8361/8356/8359/4855/8294/8352/84441
R-HSA-72766	Translation	287	0.511136678	1.905092013	3.03E-10	2.98E-08	2.55E-08	200916/1917/26986/9553/64969/1978/7818/7453/1974/6176/51069/64979/6169/90480/219927/6189/6233/23480/8891/55173/6232/3735/6207/6206/8664/7407/6170/51073/64928/6230/10480/6155/1936/8565/51520/6158/8666/29104/8663/6183/4528/54460/64951/6231/6165/2935/9045/1938/65080/8667/6217/8668/6142/2107/6157/7311/8892/51649/6144/79590/6193/6125/6223/6218/51116/4677/51373/3646/6224/2617/6154/8890/1615/8894/51065/10573/6141/6301/6234/6222/2193/51121/6235/128308/65008/3035/6147/6210/6152/6188/25873/51386/1933/9521/6181/9349/28973/6229/6173/64975/8669/3376/63875/118487/65005/6135/6136/64983/327/6167/60488/6204/8662/8661/6124/6201/9669/64949/6161/10056/5464/6228/6175/29074/122704/6146/6156/84340/1968/6209/23521/6731/11224/6730/6159/7284/6122/6132/6128/6191/6182/90701/28977/64960/51650/6134/6227/60559/54148/54534/10884/6168/51263/6150/27335/6143/64978/6202/6194/6205/64981/92935/3921/6137/8665/6160/26589/6133/1973/6203/29088/1981/28957/9801/6746/6187/64968/55052/7965/51253/56945/6139/4736/124995/54938/6897/6748/23107/55699/79731/1915/54998/922
R-HSA-201722	Formation of the beta-catenin:TCF transactivating complex	89	-0.616279121	-2.352344897	3.65E-10	3.42E-08	2.92E-08	8363/8353/7089/6932/3065/3020/8338/8347/3021/8362/8341/333932/126961/8370/554313/8339/8355/8334/3014/8367/8290/121504/653604/8368/8348/8364/55766/8335/8968/8366/3017/474382/8343/8344/8354/3012/8360/8351/8365/51176/94239/3015/8346/7088/8358/8350/8357/8342/8331/8340/8345/8361/8356/8359/8294/83439/8313/8352

R-HSA-6791226	Major pathway of rRNA processing in the nucleolus and cytosol	181	0.557415816	1.987610662	8.82E-10	7.85E-08	6.70E-08	100861532/102800317/10799/200916/23246/9136/28987/9188/6176/6169/6189/6233/54512/8568/6232/6207/6206/23160/6170/26354/51202/2091/6230/8780/6155/9277/6158/56915/5393/10969/25879/6231/6165/9045/11102/65083/55131/6217/6142/10557/55720/6157/134430/7311/55813/6144/6193/6125/6223/6218/6224/84916/6154/51065/56902/55759/6141/6234/6222/51121/6235/6147/6210/6152/6188/25873/6181/9349/6229/4691/6173/10607/51118/6135/705/6136/114049/23016/6167/6204/26168/6124/6201/79050/6161/6228/6175/6146/6156/6209/23521/4809/57418/11224/54913/11103/6159/6122/6132/6128/6191/55127/6134/6227/1454/27042/81887/51388/6168/57647/84128/10199/6143/6202/6194/11056/6205/10171/8602/22984/3921/6137/6160/27043/10885/92856/9790/54555/6133/6203/6187/6139/4736
R-HSA-3214858	RMTs methylate histone arginines	77	-0.638937439	-2.391399569	9.70E-10	8.25E-08	7.04E-08	8362/55193/317772/196528/333932/126961/8370/554313/8330/8355/8332/8334/3014/8367/8336/121504/653604/8368/8364/55766/8335/8968/8366/8969/474382/8354/3012/8360/8351/8365/8329/94239/3015/85235/8358/8350/8357/8331/8361/8356/8359/8294/8352
R-HSA-211000	Gene Silencing by RNA	129	-0.544496821	-2.173685474	1.13E-09	9.17E-08	7.82E-08	8347/54487/3021/8362/8341/333932/126961/8370/554313/8339/8355/8334/3014/8367/121504/653604/8368/8348/8364/55766/8335/8968/8366/3017/474382/8343/143689/8344/8354/3012/8360/8351/8365/94239/3015/8346/8358/8350/55124/8357/8342/8331/8340/8345/8361/8356/4603/8359/8294/8468/8352
R-HSA-606279	Deposition of new CENPA-containing nucleosomes at the centromere	72	-0.647401659	-2.377852277	1.75E-09	1.31E-07	1.11E-07	55355/8338/79172/64105/8347/8362/8341/55166/57082/8370/554313/378708/8339/8334/79019/11339/3014/8367/387103/121504/8368/8348/8364/55766/8335/8366/3017/474382/2491/8343/8344/3012/8360/8365/94239/3015/8346/100526739/8342/8331/8340/8345/8361/8359/8294
R-HSA-774815	Nucleosome assembly	72	-0.647401659	-2.377852277	1.75E-09	1.31E-07	1.11E-07	55355/8338/79172/64105/8347/8362/8341/55166/57082/8370/554313/378708/8339/8334/79019/11339/3014/8367/387103/121504/8368/8348/8364/55766/8335/8366/3017/474382/2491/8343/8344/3012/8360/8365/94239/3015/8346/100526739/8342/8331/8340/8345/8361/8359/8294
R-HSA-68875	Mitotic Prophase	139	-0.534507943	-2.137563329	1.96E-09	1.39E-07	1.19E-07	8970/255919/9663/891/5594/79902/54892/81876/8363/8353/7112/140609/3020/8338/5595/8347/3021/8362/8341/333932/126961/8370/554313/8339/4926/8355/8334/3014/8367/8290/121504/653604/8368/8348/8364/55766/8335/8968/8366/4001/3017/10592/474382/8343/8344/8354/3012/8360/8351/8365/94239/3015/8346/8358/8350/8357/8342/8331/8340/8345/8361/8356/8359/8294/8352
R-HSA-8868773	rRNA processing in the nucleus and cytosol	191	0.549175047	1.969052528	2.01E-09	1.39E-07	1.19E-07	100861532/102800317/55505/10799/200916/23246/9136/28987/9188/27292/6176/6169/6189/6233/54512/8568/6232/6207/6206/23160/6170/26354/51202/2091/6230/8780/6155/9277/6158/56915/5393/10969/25879/6231/6165/9045/11102/65083/55131/6217/6142/10557/55720/6157/134430/7311/55813/115939/6144/6193/6125/6223/6218/6224/84916/6154/51065/56902/55759/6141/6234/6222/51121/6235/6147/6210/6152/6188/25873/6181/9349/6229/4691/6173/10607/51118/6135/705/6136/114049/23016/6167/6204/26168/6124/6201/79050/6161/6228/6175/6146/6156/6209/23521/4809/57418/11224/54913/11103/6159/6122/6132/6128/6191/55127/6134/6227/1454/27042/81887/51388/6168/57647/84128/10199/6143/6202/6194/11056/6205/10171/8602/22984/3921/6137/6160/27043/10885/92856/9790/54555/6133/6203/4839/6187/6139/4736

R-HSA-1280215	Cytokine Signaling in Immune system	614	0.418555379	1.633869989	3.07E-09	2.05E-07	1.75E-07	53832/3569/9173/6775/402569/2635/7850/4312/3576/3557/8809/8784/3606/5698/4314/3589/2323/23650/5657/3934/1436/1848/51191/8519/5696/90865/9180/960/2534/5971/942/3587/6097/301/1440/27189/8638/282618/3635/3689/2185/10410/3575/3383/3568/3684/3437/11074/2357/1271/3656/3556/3936/4609/11009/84166/330/3669/1026/3091/3433/3566/5292/1896/9246/2335/3570/58985/3601/302/3726/3394/414062/3115/3429/5743/10107/3055/356/3605/3119/10581/4791/1435/4938/8808/26525/122706/6349/3662/10913/3598/6776/3929/60401/3976/6672/25939/7409/4261/9636/1958/6804/4582/91543/3106/7422/10437/3665/5724/3460/9306/7318/6774/7040/3660/8744/128178/3134/3688/9830/1849/3105/9021/6624/64127/3459/3430/654483/1846/10538/6059/6197/23198/1052/79923/8764/53840/3725/9020/1265/6283/6095/5699/604/4502/10379/4088/10019/6196/3434/51330/11097/9466/7006/1974/4055/4170/23321/841/4507/7324/6233/3597/57162/3133/53833/7187/7076
R-HSA-5250924	B-WICH complex positively regulates rRNA expression	89	-0.595883143	-2.274493204	3.32E-09	2.14E-07	1.83E-07	8347/3021/8362/8341/333932/126961/8370/554313/8339/8355/8334/3014/8367/121504/653604/8368/8348/8364/8850/55766/8335/8968/8366/3017/474382/8343/8344/8354/3012/8360/8351/8365/94239/3015/8346/8358/8350/8357/8342/8331/8340/8345/8361/8356/8359/8294/8352
R-HSA-73772	RNA Polymerase I Promoter Escape	89	-0.594662333	-2.269833356	3.96E-09	2.47E-07	2.11E-07	8347/3021/8362/8341/333932/126961/8370/554313/8339/8355/8334/3014/8367/121504/653604/8368/8348/8364/55766/8335/8968/8366/3017/474382/8343/8344/8354/3012/8360/8351/8365/94239/3015/8346/8358/8350/8357/8342/8331/8340/8345/8361/8356/8359/8294/8352
R-HSA-5625886	Activated PKN1 stimulates transcription of AR (androgen receptor) regulated genes KLK2 and KLK3	65	-0.654664403	-2.389556864	4.44E-09	2.54E-07	2.17E-07	8347/3021/8362/8341/333932/126961/8370/554313/8339/8355/10499/8334/3014/8367/121504/653604/8368/8348/8364/55766/8335/8968/8366/3017/474382/8343/8344/8354/3012/8360/8351/8365/94239/3015/8346/8358/8350/8357/8342/8331/8340/8345/8361/8356/8359/367/8294/8352
R-HSA-5625740	RHO GTPases activate PKNs	92	-0.598050926	-2.294863421	4.46E-09	2.54E-07	2.17E-07	8347/3021/4629/8362/995/8341/333932/126961/8370/554313/8339/8355/10499/8334/3014/8367/121504/653604/8368/8348/8364/55766/8335/8968/8366/3017/474382/8343/8344/8354/3012/8360/8351/8365/94239/3015/8346/8358/8350/8357/8342/8331/8340/8345/8361/8356/388/8359/367/8294/8352
R-HSA-72312	rRNA processing	201	0.534409668	1.924578243	4.48E-09	2.54E-07	2.17E-07	100861532/102800317/55505/10799/200916/23246/9136/28987/9188/27292/6176/6169/6189/6233/54512/8568/6232/6207/6206/23160/6170/26354/51202/2091/6230/8780/6155/9277/6158/56915/5393/10969/25879/6231/6165/9045/11102/65083/55131/6217/6142/10557/55720/6157/134430/55178/7311/55813/115939/6144/6193/6125/6223/6218/6224/84916/6154/51065/56902/55759/6141/6234/6222/51121/6235/6147/6210/6152/6188/25873/6181/9349/6229/4691/6173/10607/51118/6135/705/6136/114049/23016/6167/6204/26168/6124/6201/79050/6161/6228/6175/6146/6156/6209/23521/4809/57418/79922/11224/54913/11103/6159/6122/6132/6128/6191/60528/55127/6134/6227/1454/27042/81887/51388/6168/57647/84128/10199/6143/6202/6194/11056/6205/10171/8602/22984/3921/51106/6137/6160/27043/10885/92856/9790/54555/6133/6203/4839/6187/6139/4736
R-HSA-171306	Packaging Of Telomere Ends	50	-0.704493123	-2.409081664	7.56E-09	4.16E-07	3.55E-07	8347/8362/8341/8370/554313/8339/8334/3014/8367/8290/121504/8368/8348/8364/55766/8335/8366/3017/474382/8343/8344/3012/8360/8365/94239/3015/8346/8342/8331/8340/8345/8361/8359/8294
R-HSA-5617472	Activation of anterior HOX genes in hindbrain development during early embryogenesis	115	-0.541369869	-2.134935828	1.42E-08	7.36E-07	6.28E-07	3020/22976/8338/8347/3021/8362/9611/8341/333932/126961/8370/554313/8339/8355/8334/3014/8367/121504/653604/8368/8348/8364/55766/8335/8968/8366/3017/474382/8343/5914/8344/8354/6256/3012/8360/8351/8365/94239/3015/8346/8358/9935/8350/8357/8342/8331/8340/8345/8361/8356/8359/4211/8294/3221/8352

R-HSA-5619507	Activation of HOX genes during differentiation	115	-0.541369869	-2.134935828	1.42E-08	7.36E-07	6.28E-07	3020/22976/8338/8347/3021/8362/9611/8341/333932/126961/8370/554313/8339/8355/8334/3014/8367/121504/653604/8368/8348/8364/55766/8335/8968/8366/3017/474382/8343/5914/8344/8354/6256/3012/8360/8351/8365/94239/3015/8346/8358/9935/8350/8357/8342/8331/8340/8345/8361/8356/8359/4211/8294/3221/8352
R-HSA-110330	Recognition and association of DNA glycosylase with site containing an affected purine	54	-0.684393454	-2.39142403	1.64E-08	7.87E-07	6.71E-07	8347/8362/8341/8370/554313/8339/8334/3014/8367/8290/121504/8368/8348/8364/55766/8335/8366/3017/474382/8343/8344/3012/8360/8365/94239/3015/8346/8342/8331/8340/8345/8361/8359/8294
R-HSA-110331	Cleavage of the damaged purine	54	-0.684393454	-2.39142403	1.64E-08	7.87E-07	6.71E-07	8347/8362/8341/8370/554313/8339/8334/3014/8367/8290/121504/8368/8348/8364/55766/8335/8366/3017/474382/8343/8344/3012/8360/8365/94239/3015/8346/8342/8331/8340/8345/8361/8359/8294
R-HSA-73927	Depurination	54	-0.684393454	-2.39142403	1.64E-08	7.87E-07	6.71E-07	8347/8362/8341/8370/554313/8339/8334/3014/8367/8290/121504/8368/8348/8364/55766/8335/8366/3017/474382/8343/8344/3012/8360/8365/94239/3015/8346/8342/8331/8340/8345/8361/8359/8294
R-HSA-1474165	Reproduction	133	-0.514146493	-2.051925637	2.39E-08	1.07E-06	9.17E-07	10274/26277/8363/4683/8353/6119/25777/5888/641/3020/4361/8338/8347/3021/11083/5885/8362/8748/8341/675/333932/126961/8370/554313/8339/8355/8334/3014/8367/8290/121504/653604/84329/8368/8348/256126/8364/55766/8335/8968/8366/50511/4001/928/3017/474382/8343/8344/347732/8354/10388/3012/8360/8351/10734/8365/94239/3015/8346/8358/8350/8357/8342/23345/8331/8340/8345/23224/113177/8361/8356/8359/57828/8294/8468/22917/8352
R-HSA-3247509	Chromatin modifying enzymes	269	-0.411705728	-1.799762809	2.41E-08	1.07E-06	9.17E-07	84787/10474/1386/55170/6605/79595/5928/9575/8289/23326/29072/5253/10919/6602/23338/23067/9070/8970/7403/10524/79813/79723/10362/55250/55869/64426/58508/54496/84289/55904/6598/8363/8353/6595/3065/2146/7468/64754/6871/8338/2122/55167/55729/6907/55929/8347/7404/440689/79685/5926/8362/55193/317772/79918/9611/8341/196528/333932/126961/8370/554313/8330/8339/8355/10499/8332/6839/8334/3014/8367/8336/121504/4790/653604/8368/57325/54556/8348/8364/8850/55766/8335/8968/23522/8366/8969/3017/474382/8343/8344/51702/8354/3012/8360/8351/8365/8329/94239/3015/8346/85235/8358/8350/8357/8342/8331/8340/8345/8361/8356/23569/8359/8294/11240/8352
R-HSA-4839726	Chromatin organization	269	-0.411705728	-1.799762809	2.41E-08	1.07E-06	9.17E-07	84787/10474/1386/55170/6605/79595/5928/9575/8289/23326/29072/5253/10919/6602/23338/23067/9070/8970/7403/10524/79813/79723/10362/55250/55869/64426/58508/54496/84289/55904/6598/8363/8353/6595/3065/2146/7468/64754/6871/8338/2122/55167/55729/6907/55929/8347/7404/440689/79685/5926/8362/55193/317772/79918/9611/8341/196528/333932/126961/8370/554313/8330/8339/8355/10499/8332/6839/8334/3014/8367/8336/121504/4790/653604/8368/57325/54556/8348/8364/8850/55766/8335/8968/23522/8366/8969/3017/474382/8343/8344/51702/8354/3012/8360/8351/8365/8329/94239/3015/8346/85235/8358/8350/8357/8342/8331/8340/8345/8361/8356/23569/8359/8294/11240/8352
R-HSA-9610379	HCMV Late Events	114	-0.529912392	-2.088150418	3.30E-08	1.44E-06	1.23E-06	8347/440689/8362/317772/8341/333932/126961/8370/554313/8330/8339/8355/8332/79643/8334/8367/8336/121504/653604/8368/8348/8364/8335/8968/8366/8969/3017/8343/8344/8354/3012/8360/8351/8365/8329/8346/85235/8358/8350/8357/8342/8331/8340/8345/8361/8356/8359/8294/8352
R-HSA-72689	Formation of a pool of free 40S subunits	98	0.615405885	2.054257647	4.25E-08	1.81E-06	1.54E-06	200916/6176/6169/6189/6233/6232/6207/6206/8664/6170/6230/10480/6155/6158/8666/8663/6231/6165/9045/8667/6217/8668/6142/6157/7311/6144/6193/6125/6223/6218/3646/6224/6154/51065/6141/6234/6222/51121/6235/6147/6210/6152/6188/25873/51386/6181/9349/6229/6173/8669/6135/6136/6167/6204/8662/8661/6124/6201/6161/6228/6175/6146/6156/6209/23521/11224/6159/6122/6132/6128/6191/6134/6227/6168/27335/6143/6202/6194/6205/3921/6137/8665/6160/6133/6203/6187/6139/4736

R-HSA-110328	Recognition and association of DNA glycosylase with site containing an affected pyrimidine	59	-0.654038509	-2.329751848	4.58E-08	1.82E-06	1.55E-06	8347/8362/8341/79661/8370/554313/8339/7374/8334/3014/8367/8290/121504/8368/8348/8364/55766/8335/8366/3017/474382/8343/8344/3012/8360/8365/94239/3015/8346/8342/8331/8340/8345/8361/8359/8294
R-HSA-110329	Cleavage of the damaged pyrimidine	59	-0.654038509	-2.329751848	4.58E-08	1.82E-06	1.55E-06	8347/8362/8341/79661/8370/554313/8339/7374/8334/3014/8367/8290/121504/8368/8348/8364/55766/8335/8366/3017/474382/8343/8344/3012/8360/8365/94239/3015/8346/8342/8331/8340/8345/8361/8359/8294
R-HSA-73928	Depyrimidination	59	-0.654038509	-2.329751848	4.58E-08	1.82E-06	1.55E-06	8347/8362/8341/79661/8370/554313/8339/7374/8334/3014/8367/8290/121504/8368/8348/8364/55766/8335/8366/3017/474382/8343/8344/3012/8360/8365/94239/3015/8346/8342/8331/8340/8345/8361/8359/8294
R-HSA-5250913	Positive epigenetic regulation of rRNA expression	104	-0.550043825	-2.125280235	4.67E-08	1.82E-06	1.55E-06	8347/3021/8362/8341/333932/126961/8370/554313/8339/8355/8334/3014/8367/121504/653604/8368/8348/8364/8850/55766/8335/8968/8366/3017/474382/8343/8344/8354/3012/8360/8351/8365/94239/3015/8346/8358/8350/8357/8342/8331/8340/8345/8361/8356/8359/8294/8352
R-HSA-9670095	Inhibition of DNA recombination at telomere	66	-0.636205258	-2.311510547	5.84E-08	2.23E-06	1.90E-06	3020/8338/8347/3021/8362/8341/8370/554313/8339/8334/3014/8367/8290/121504/8368/8348/8364/55766/8335/8366/3017/474382/8343/8344/3012/8360/8365/94239/3015/8346/8342/8331/8340/8345/8361/8359/8294
R-HSA-72613	Eukaryotic Translation Initiation	116	0.588766853	2.009687063	7.24E-08	2.65E-06	2.26E-06	200916/26986/1978/1974/6176/6169/6189/6233/8891/6232/6207/6206/8664/6170/6230/10480/6155/6158/8666/8663/6231/6165/9045/8667/6217/8668/6142/6157/7311/8892/6144/6193/6125/6223/6218/3646/6224/6154/8890/8894/51065/6141/6234/6222/51121/6235/6147/6210/6152/6188/25873/51386/6181/9349/6229/6173/8669/6135/6136/6167/6204/8662/8661/6124/6201/9669/6161/6228/6175/6146/6156/1968/6209/23521/11224/6159/6122/6132/6128/6191/6134/6227/6168/27335/6143/6202/6194/6205/3921/6137/8665/6160/6133/1973/6203/1981/6187/6139/4736/1975/1965/6208/8893/6129/1967
R-HSA-72737	Cap-dependent Translation Initiation	116	0.588766853	2.009687063	7.24E-08	2.65E-06	2.26E-06	200916/26986/1978/1974/6176/6169/6189/6233/8891/6232/6207/6206/8664/6170/6230/10480/6155/6158/8666/8663/6231/6165/9045/8667/6217/8668/6142/6157/7311/8892/6144/6193/6125/6223/6218/3646/6224/6154/8890/8894/51065/6141/6234/6222/51121/6235/6147/6210/6152/6188/25873/51386/6181/9349/6229/6173/8669/6135/6136/6167/6204/8662/8661/6124/6201/9669/6161/6228/6175/6146/6156/1968/6209/23521/11224/6159/6122/6132/6128/6191/6134/6227/6168/27335/6143/6202/6194/6205/3921/6137/8665/6160/6133/1973/6203/1981/6187/6139/4736/1975/1965/6208/8893/6129/1967
R-HSA-73854	RNA Polymerase I Promoter Clearance	108	-0.539658114	-2.09700069	8.94E-08	3.21E-06	2.74E-06	3065/7343/3020/8338/5595/8347/3021/8362/8341/333932/126961/8370/554313/8339/8355/8334/3014/8367/121504/653604/8368/8348/8364/8850/55766/8335/8968/8366/3017/474382/8343/8344/8354/3012/8360/8351/8365/94239/3015/8346/8358/8350/8357/8342/8331/8340/8345/8361/8356/8359/8294/8352
R-HSA-9645723	Diseases of programmed cell death	101	-0.54686658	-2.122347343	9.39E-08	3.29E-06	2.81E-06	8347/3021/8362/995/8341/333932/126961/8370/554313/8339/8355/8334/3014/8367/121504/653604/8368/8348/8364/55766/8335/8968/8366/4001/3017/474382/8343/1789/8344/8354/3012/8360/8351/8365/94239/3015/8346/8358/8350/8357/8342/8331/8340/8345/8361/8356/8359/8294/8352
R-HSA-156842	Eukaryotic Translation Elongation	90	0.608005067	2.0063152	9.50E-08	3.29E-06	2.81E-06	200916/1917/6176/6169/6189/6233/6232/6207/6206/6170/6230/6155/1936/6158/6231/6165/9045/1938/6217/6142/6157/7311/6144/6193/6125/6223/6218/6224/6154/51065/6141/6234/6222/51121/6235/6147/6210/6152/6188/25873/1933/6181/9349/6229/6173/6135/6136/6167/6204/6124/6201/6161/6228/6175/6146/6156/6209/23521/11224/6159/6122/6132/6128/6191/6134/6227/6168/6143/6202/6194/6205/3921/6137/6160/6133/6203/6187/6139/4736

R-HSA-2408557	Selenocysteine synthesis	90	0.605392312	1.997693545	1.19E-07	4.03E-06	3.44E-06	118672/200916/6176/6169/6189/6233/6232/6207/6206/6170/6230/6155/6158/6231/6165/9045/6217/6142/6157/7311/6144/6193/6125/6223/6218/6224/6154/51065/6141/6301/6234/6222/51121/6235/6147/6210/6152/6188/25873/6181/9349/6229/6173/6135/6136/6167/6204/6124/6201/6161/6228/6175/6146/6156/6209/23521/11224/6159/6122/6132/6128/6191/6134/6227/6168/6143/6202/6194/6205/3921/6137/6160/6133/6203/6187/6139/4736
R-HSA-9633012	Response of EIF2AK4 (GCN2) to amino acid deficiency	98	0.601630405	2.008274361	1.41E-07	4.71E-06	4.02E-06	467/1051/1649/200916/6176/6169/6189/6233/6232/6207/6206/6170/6230/6155/6158/6231/6165/9045/6217/6142/6157/7311/6144/6193/440/6125/6223/6218/6224/6154/8894/51065/6141/6234/6222/51121/6235/6147/6210/6152/6188/25873/6181/9349/6229/6173/6135/6136/6167/6204/6124/6201/6161/6228/6175/6146/6156/1968/6209/23521/440275/11224/468/6159/10985/6122/6132/6128/6191/6134/6227/6168/6143/6202/6194/6205/3921/6137/6160/1054/6133/6203/6187/6139/4736
R-HSA-73929	Base-Excision Repair, AP Site Formation	61	-0.641646428	-2.306350712	1.45E-07	4.77E-06	4.07E-06	8347/8362/8341/79661/8370/554313/8339/7374/8334/3014/8367/8290/121504/8368/8348/8364/55766/8335/8366/3017/474382/8343/8344/3012/8360/8365/94239/3015/8346/8342/8331/8340/8345/8361/8359/8294
R-HSA-73884	Base Excision Repair	90	-0.565231655	-2.158118734	1.50E-07	4.85E-06	4.14E-06	5985/26277/23583/8363/5426/7515/6119/8338/2237/8347/5427/8362/8341/3978/79661/8370/554313/8339/7374/8334/3014/8367/8290/121504/8368/8348/8364/55766/8335/8366/3017/474382/8343/8344/3012/8360/8365/94239/3015/8346/8342/8331/8340/8345/8361/8359/8294
R-HSA-72764	Eukaryotic Translation Termination	89	0.614726417	2.023342694	1.61E-07	5.09E-06	4.35E-06	200916/6176/6169/6189/6233/6232/6207/6206/6170/6230/6155/6158/29104/6231/6165/2935/9045/6217/6142/2107/6157/7311/6144/6193/6125/6223/6218/6224/6154/51065/6141/6234/6222/51121/6235/6147/6210/6152/6188/25873/6181/9349/6229/6173/6135/6136/327/6167/6204/6124/6201/6161/6228/6175/6146/6156/6209/23521/11224/6159/6122/6132/6128/6191/6134/6227/6168/6143/6202/6194/6205/3921/6137/6160/6133/6203/6187/6139/4736
R-HSA-72706	GTP hydrolysis and joining of the 60S ribosomal subunit	109	0.590394391	1.997784597	1.69E-07	5.28E-06	4.51E-06	200916/1974/6176/6169/6189/6233/6232/6207/6206/8664/6170/6230/10480/6155/6158/8666/8663/6231/6165/9045/8667/6217/8668/6142/6157/7311/6144/6193/6125/6223/6218/3646/6224/6154/8894/51065/6141/6234/6222/51121/6235/6147/6210/6152/6188/25873/51386/6181/9349/6229/6173/8669/6135/6136/6167/6204/8662/8661/6124/6201/9669/6161/6228/6175/6146/6156/1968/6209/23521/11224/6159/6122/6132/6128/6191/6134/6227/6168/27335/6143/6202/6194/6205/3921/6137/8665/6160/6133/1973/6203/1981/6187/6139/4736
R-HSA-2559580	Oxidative Stress Induced Senescence	120	-0.514129956	-2.051861678	1.98E-07	6.07E-06	5.18E-06	8363/84733/8353/5600/2146/3020/8338/5595/8347/3021/8362/8341/333932/126961/5608/8370/554313/8339/8355/5602/8334/3014/8367/121504/653604/1031/8368/8348/1869/8364/55766/8335/8968/8366/57332/3017/100532731/474382/8343/8344/1870/8354/3012/8360/8351/8365/94239/3015/8346/8358/23466/8350/8357/8342/8331/8340/8345/8361/8356/8359/8294/8352
R-HSA-156827	L13a-mediated translational silencing of Ceruloplasmin expression	108	0.592805479	2.003221572	2.43E-07	7.33E-06	6.26E-06	200916/26986/1974/6176/6169/6189/6233/6232/6207/6206/8664/6170/6230/10480/6155/6158/8666/8663/6231/6165/9045/8667/6217/8668/6142/6157/7311/6144/6193/6125/6223/6218/3646/6224/6154/8894/51065/6141/6234/6222/51121/6235/6147/6210/6152/6188/25873/51386/6181/9349/6229/6173/8669/6135/6136/6167/6204/8662/8661/6124/6201/6161/6228/6175/6146/6156/1968/6209/23521/11224/6159/6122/6132/6128/6191/6134/6227/6168/27335/6143/6202/6194/6205/3921/6137/8665/6160/6133/1973/6203/1981/6187/6139/4736
R-HSA-192823	Viral mRNA Translation	86	0.611531957	2.011356943	2.73E-07	8.11E-06	6.92E-06	200916/6176/6169/6189/6233/6232/6207/6206/6170/6230/6155/6158/6231/6165/9045/6217/6142/6157/7311/6144/6193/6125/6223/6218/6224/6154/51065/6141/6234/6222/51121/6235/6147/6210/6152/6188/25873/6181/9349/6229/6173/6135/6136/6167/6204/6124/6201/6161/6228/2926/6175/6146/6156/6209/23521/11224/6159/6122/6132/6128/6191/6134/6227/6168/6143/6202/6194/6205/3921/6137/6160/6133/6203/6187/6139/4736

R-HSA-975956	Nonsense Mediated Decay (NMD) independent of the Exon Junction Complex (EJC)	91	0.609780823	2.014439554	2.88E-07	8.30E-06	7.08E-06	200916/26986/6176/6169/6189/6233/6232/6207/6206/6170/6230/6155/6158/6231/6165/2935/9045/6217/6142/2107/6157/7311/6144/6193/6125/6223/6218/6224/6154/51065/6141/6234/6222/51121/6235/6147/6210/6152/6188/25873/6181/9349/6229/6173/6135/6136/6167/6204/6124/6201/6161/6228/6175/6146/6156/6209/23521/11224/6159/6122/6132/6128/6191/6134/6227/6168/6143/6202/6194/6205/3921/6137/6160/6133/6203/1981/6187/6139/4736
R-HSA-156902	Peptide chain elongation	86	0.610663417	2.008500273	2.87E-07	8.30E-06	7.08E-06	200916/6176/6169/6189/6233/6232/6207/6206/6170/6230/6155/6158/6231/6165/9045/1938/6217/6142/6157/7311/6144/6193/6125/6223/6218/6224/6154/51065/6141/6234/6222/51121/6235/6147/6210/6152/6188/25873/6181/9349/6229/6173/6135/6136/6167/6204/6124/6201/6161/6228/6175/6146/6156/6209/23521/11224/6159/6122/6132/6128/6191/6134/6227/6168/6143/6202/6194/6205/3921/6137/6160/6133/6203/6187/6139/4736/1915
R-HSA-427413	NoRC negatively regulates rRNA expression	105	-0.526410484	-2.037359563	3.43E-07	9.73E-06	8.30E-06	8347/3021/79685/8362/8341/333932/126961/8370/554313/8339/8355/8334/3014/8367/121504/653604/8368/8348/8364/55766/8335/8968/8366/3017/474382/8343/1789/8344/8354/3012/8360/8351/8365/94239/3015/8346/8358/8350/8357/8342/8331/8340/8345/8361/8356/8359/8294/8352
R-HSA-977225	Amyloid fiber formation	97	-0.539144825	-2.093760217	4.43E-07	1.24E-05	1.06E-05	4069/3020/8338/8347/3021/8362/8341/23621/333932/126961/8370/554313/8339/8355/8334/3014/8367/121504/653604/8368/8348/8364/8335/8968/8366/3017/474382/8343/8344/8354/3012/8360/8351/8365/3015/8346/793/8358/8350/8357/8342/8331/8340/8345/83464/8361/8356/8359/10098/8294/8352
R-HSA-5250941	Negative epigenetic regulation of rRNA expression	108	-0.523031709	-2.032393892	5.18E-07	1.43E-05	1.22E-05	8347/3021/79685/8362/8341/333932/126961/8370/554313/8339/8355/6839/8334/3014/8367/121504/653604/8368/8348/8364/55766/8335/8968/8366/3017/474382/8343/1789/8344/8354/3012/8360/8351/8365/94239/3015/8346/8358/8350/8357/8342/8331/8340/8345/8361/8356/8359/8294/8352
R-HSA-212165	Epigenetic regulation of gene expression	147	-0.463908252	-1.8583823	9.84E-07	2.67E-05	2.28E-05	8347/3021/79685/8362/8341/333932/126961/8370/554313/8339/8355/6839/8334/3014/8367/121504/653604/8368/8348/8364/8850/55766/8335/8968/8366/3017/474382/8343/1789/8344/8354/3012/8360/8351/8365/94239/3015/54790/80312/8346/8358/8350/8357/8342/8331/8340/8345/8361/8356/8359/29947/8294/8352
R-HSA-927802	Nonsense-Mediated Decay (NMD)	111	0.565088131	1.915580573	1.13E-06	2.97E-05	2.53E-05	200916/26986/6176/6169/6189/6233/6232/6207/6206/6170/6230/6155/6158/6231/6165/2935/9045/6217/6142/2107/6157/7311/6144/6193/5520/6125/6223/6218/6224/6154/51065/6141/6234/6222/51121/6235/6147/6210/6152/6188/25873/6181/9349/6229/6173/4116/6135/6136/6167/6204/6124/6201/6161/6228/6175/6146/6156/6209/23521/11224/6159/6122/6132/6128/6191/6134/6227/6168/6143/6202/6194/6205/10921/3921/6137/6160/23381/6133/6203/1981/9887/6187/6139/4736
R-HSA-975957	Nonsense Mediated Decay (NMD) enhanced by the Exon Junction Complex (EJC)	111	0.565088131	1.915580573	1.13E-06	2.97E-05	2.53E-05	200916/26986/6176/6169/6189/6233/6232/6207/6206/6170/6230/6155/6158/6231/6165/2935/9045/6217/6142/2107/6157/7311/6144/6193/5520/6125/6223/6218/6224/6154/51065/6141/6234/6222/51121/6235/6147/6210/6152/6188/25873/6181/9349/6229/6173/4116/6135/6136/6167/6204/6124/6201/6161/6228/6175/6146/6156/6209/23521/11224/6159/6122/6132/6128/6191/6134/6227/6168/6143/6202/6194/6205/10921/3921/6137/6160/23381/6133/6203/1981/9887/6187/6139/4736
R-HSA-6809371	Formation of the cornified envelope	70	0.623110507	1.988658666	1.19E-06	3.07E-05	2.62E-05	3887/6699/8688/5266/1041/6703/11202/6707/3892/3872/6698/353135/3855/643414/1823/144501/3868/23581/404203/3882/3857/3849/3891/3853/353141/25818/3889/125115/1824
R-HSA-73886	Chromosome Maintenance	139	-0.475822333	-1.902872322	1.20E-06	3.07E-05	2.62E-05	5422/55320/5985/26277/63922/8363/1060/6119/641/55355/3020/55135/8338/79172/2237/64105/8347/3021/8362/8341/55166/57082/3978/80119/8370/554313/378708/8339/8334/79019/11339/3014/8367/387103/8290/121504/8368/8348/8364/55766/8335/8366/3017/474382/2491/8343/8344/3012/8360/8365/94239/3015/8346/100526739/8342/8331/8340/8345/8361/8359/8294

R-HSA-449147	Signaling by Interleukins	404	0.417394803	1.595114545	1.73E-06	4.38E-05	3.73E-05	53832/3569/9173/6775/7850/4312/3576/3557/8809/3606/5698/4314/3589/5657/3934/1436/1848/5696/90865/9180/2534/942/3587/6097/301/1440/27189/282618/3635/3689/2185/3575/3383/3568/3684/2357/1271/3656/3556/3936/4609/11009/84166/1026/3091/3566/5292/2335/3570/58985/3601/302/3726/414062/5743/3055/356/3605/4791/1435/8808/26525/122706/6349/3662/3598/6776/3929/3976/7409/6804/4582/7422/5724/6774/7040/3688/1849/9021/6624/64127/654483/1846/10538/6197/23198/1052/79923/3725/1265/6283/6095/5699/604
R-HSA-168249	Innate Immune System	870	0.365780222	1.443460619	2.28E-06	5.69E-05	4.86E-05	2266/3310/6278/2244/629/4846/5698/6280/6286/5266/51297/1520/5657/710/6317/6590/8972/3934/140683/1848/51191/92747/57126/5696/84659/960/2534/5971/114548/978/338324/7097/968/837/22914/3428/8993/5337/6279/23406/10326/3689/79895/54210/3684/5627/4585/4586/9961/2357/3656/1116/84166/7128/283463/284266/5329/330/1823/4651/25797/5799/4584/5265/22861/10622/6282/9246/11031/54941/22875/11001/302/23601/417/11026/1380/388697/122618/78989/147945/2950/3055/2206/84658/1794/2243/2512/4791/10394/4689/613211/122706/3579/1536/566/4842/54509/2171/9844/3929/719/1514/84418/353376/7409/10810/2040/64135/9636/4582/116842/3106/5836/90423/8976/3665/1143/976/1687/5724/12/6793/7318/10875/79132/11010/55313/5768/4311/64170/966/23220/272/7077/116844/4860/1540/1849/10493/25798/3105/10584/5336/64127/148022/634/1846/1992/3708/654364/6197/23198/3725/127829/9020/1265/1/6283/5699/718/8915/5912/245972/7454/6196/5058/6386/7006/143662/5236/475/11277/8987/200958/1476/728/842/3984/11322/3163/5435/201294/9296/10095/841/71/9588/6233/2760/23593/57162/3133/4680/64386/718
R-HSA-9616222	Transcriptional regulation of granulopoiesis	87	-0.53518585	-2.032311098	2.43E-06	5.99E-05	5.11E-05	8347/3021/8362/8341/333932/126961/8370/554313/8339/8355/8334/3014/8367/121504/653604/8368/8348/1869/8364/55766/8335/8968/8366/3017/474382/8343/5914/8344/8354/6256/3012/8360/8351/8365/51176/94239/3015/8346/8358/8350/8357/8342/8331/8340/8345/1050/8361/8356/4602/8359/8294/8352
R-HSA-1799339	SRP-dependent cotranslational protein targeting to membrane	109	0.561203775	1.89900899	3.08E-06	7.48E-05	6.38E-05	200916/6176/6169/6189/6233/23480/6232/6207/6206/6170/6230/6155/6158/6231/6165/9045/6217/6142/6157/7311/6144/6193/6125/6223/6218/6224/6154/51065/6141/6234/6222/51121/6235/6147/6210/6152/6188/25873/6181/9349/6229/6173/6135/6136/6167/6204/6124/6201/6161/6228/6175/6146/6156/6209/23521/6731/11224/6730/6159/6122/6132/6128/6191/90701/6134/6227/60559/6168/6143/6202/6194/6205/3921/6137/6160/6133/6203/6746/6187/6139/4736/6748
R-HSA-69473	G2/M DNA damage checkpoint	92	-0.523259941	-2.007872651	3.49E-06	8.36E-05	7.14E-05	7157/672/79184/9156/7156/9656/7158/144715/84142/116028/7533/11073/6117/983/8970/10524/891/83695/5985/5883/11200/8363/4683/6119/641/7468/4361/165918/8347/8362/83990/995/8341/8370/554313/8339/80010/3014/8367/8290/121504/8368/8348/8364/8366/3017/8343/8344/580/8360/8365/8346/8342/8340/8345/8361/8359/8294
R-HSA-909733	Interferon alpha/beta signaling	56	0.646395505	1.987252724	3.97E-06	9.39E-05	8.02E-05	8519/5696/8638/10410/3437/3669/3433/3394/3429/10581/4938/3662/25939/9636/1958/91543/3106/3665/3660/3134/3105/9021/3430/6059/10379/3434/3133/11274/4599/6772
R-HSA-6799990	Metal sequestration by antimicrobial proteins	5	0.974517491	1.727312679	4.23E-06	9.88E-05	8.43E-05	6278/6280/3934/338324/6279
R-HSA-73864	RNA Polymerase I Transcription	110	-0.487016945	-1.898972594	5.80E-06	0.0001338	0.00011419	3065/7343/3020/8338/5595/8347/3021/8362/8341/333932/126961/8370/554313/8339/8355/8334/3014/8367/121504/653604/8368/8348/8364/8850/55766/8335/8968/8366/3017/474382/8343/8344/8354/3012/8360/8351/8365/94239/3015/8346/8358/8350/8357/8342/8331/8340/8345/8361/8356/8359/8294/8352
R-HSA-913531	Interferon Signaling	173	0.49114392	1.742685775	6.11E-06	0.0001394	0.00011892	402569/2635/23650/51191/8519/5696/960/8638/10410/3383/3437/11074/3669/3433/9246/3394/3115/3429/10107/3119/10581/4938/3662/6672/25939/4261/9636/1958/91543/3106/10437/3665/5724/3460/7318/3660/3134/9830/3105/9021/3459/3430/6059/53840/4502/10379/3434/11097/1974/23321/7324/6233/3133

R-HSA-168273	Influenza Viral RNA Transcription and Replication	132	0.511781209	1.768559944	1.13E-05	0.0002552	0.00021777	200916/11097/5435/6176/6169/6189/6233/6232/6207/6206/3320/6170/6230/6155/6158/6231/6165/9045/5438/6217/6142/6157/7311/6144/6193/6125/6223/6218/6224/6154/51065/6141/6234/6222/51121/6235/81929/2963/6147/6210/6152/5437/6188/25873/6181/9349/6229/6173/3843/6135/6136/6167/6204/6124/6201/6161/6228/2926/6175/6146/6156/6209/23521/11224/6159/6122/6132/6128/6191/6134/6227/6168/6143/6202/6194/6205/9818/3921/6137/6160/2962/6133/6203/6187/6139/4736
R-HSA-68867	Assembly of the pre-replicative complex	141	-0.440515499	-1.762060093	1.20E-05	0.0002681	0.00022875	8363/23594/8353/84515/5001/4171/3020/8338/5000/8347/3021/8362/8341/333932/126961/8370/554313/8339/8355/8334/3014/8367/121504/653604/8368/8348/8364/55766/8335/8968/8366/3017/474382/8343/8344/8354/3012/8360/8351/8365/94239/3015/8346/8358/8350/8357/8342/8331/8340/8345/8361/8356/8359/8294/8352
R-HSA-168255	Influenza Infection	153	0.49755303	1.743328211	1.36E-05	0.0002999	0.00025596	402569/9636/7040/200916/11097/5435/6176/6169/6189/6233/6232/6207/6206/3320/6170/6230/6155/6158/6231/6165/9045/5438/6217/6142/6157/7311/6144/6193/6125/6223/6218/6224/6154/51065/6141/6234/6222/51121/3840/6235/81929/2963/6147/6210/6152/5437/6188/25873/6181/9349/6229/6173/3843/6135/6136/6167/6204/6124/6201/6161/6228/2926/6175/6146/6156/6209/23521/11224/6159/6122/1211/6132/6128/6191/3841/6134/6227/6168/6143/6202/6194/6205/9818/3921/6137/6160/2962/6133/6203/6187/6139/4736/6396/5901/5440/6208/6129/3303/5611/5610/23279/5441/3839/6138
R-HSA-3214842	HDMs demethylate histones	49	-0.606379359	-2.071712719	1.41E-05	0.0003027	0.00025833	7404/8362/333932/126961/8370/554313/8355/8367/121504/653604/8368/8364/8968/8366/8354/8360/8351/8365/8358/8350/8357/8361/8356/8359/8294/8352
R-HSA-2559586	DNA Damage/Telomere Stress Induced Senescence	78	-0.530097492	-1.990562739	1.40E-05	0.0003027	0.00025833	26277/23523/8363/4683/3006/4361/8338/7290/8347/8362/8341/3008/8370/554313/8339/25842/8334/3014/8367/8290/121504/8368/8348/8364/55766/8335/9134/8366/4001/3017/474382/8343/8344/3012/8360/8365/94239/3015/3007/8346/8342/8331/8340/3009/8345/8361/8359/8294
R-HSA-6805567	Keratinization	89	0.565598929	1.861641911	1.69E-05	0.0003582	0.00030565	3887/6699/8688/5266/1041/6703/11202/6707/3892/3872/6698/353135/440021/3855/643414/83897/1823/144501/3868/23581/404203/3882/337880/3857/3849/3891/3853/353141
R-HSA-157579	Telomere Maintenance	111	-0.475098169	-1.854909695	1.85E-05	0.0003883	0.00033139	5422/5985/26277/63922/8363/6119/641/3020/55135/8338/2237/8347/3021/8362/8341/3978/80119/8370/554313/8339/8334/3014/8367/8290/121504/8368/8348/8364/55766/8335/8366/3017/474382/8343/8344/3012/8360/8365/94239/3015/8346/8342/8331/8340/8345/8361/8359/8294
R-HSA-5693571	Nonhomologous End-Joining (NHEJ)	66	-0.561888864	-2.041498428	1.90E-05	0.0003956	0.00033763	8363/4683/7468/64421/4361/22976/165918/8347/8362/8341/8370/554313/8339/3014/8367/8290/121504/8368/8348/8364/8366/3017/8343/8344/580/8360/8365/8346/8342/8340/8345/8361/8359/8294
R-HSA-3214841	PKMTs methylate histone lysines	71	-0.533459942	-1.953139297	2.01E-05	0.0004129	0.00035237	29072/10919/23067/9070/79813/79723/58508/55904/8363/8353/2146/7468/64754/2122/55729/8362/79918/333932/126961/8370/554313/8355/6839/8367/121504/4790/653604/8368/8364/8968/8366/8354/8360/8351/8365/8358/8350/8357/8361/8356/8359/8294/8352
R-HSA-9609690	HCMV Early Events	132	-0.437102015	-1.741951493	2.72E-05	0.0005466	0.00046646	8347/440689/8362/317772/9611/8341/333932/126961/8370/554313/8330/8339/81027/8355/8332/8334/8367/8336/79861/121504/4790/653604/8368/8348/8364/8335/8968/8366/8969/3017/8343/8344/7846/8354/3012/8360/8351/8365/8329/8346/85235/8358/8350/8357/8342/8331/8340/8345/113457/8361/8356/8359/8294/8352
R-HSA-69002	DNA Replication Pre-Initiation	158	-0.411144608	-1.670318497	2.69E-05	0.0005466	0.00046646	8363/23594/8353/84515/5426/5001/6119/4171/3020/8338/5000/8347/5427/3021/8362/8341/333932/126961/8370/554313/8339/8355/8334/8318/3014/8367/121504/653604/8368/8348/8364/55766/8335/8968/8366/3017/474382/8343/8344/8354/3012/8360/8351/8365/94239/3015/8346/8358/8350/8357/8342/8331/8340/8345/8361/8356/8359/8294/8352
R-HSA-140875	Common Pathway of Fibrin Clot Formation	18	0.805667877	1.971422661	2.81E-05	0.0005528	0.00047178	2266/2244/5270/5657/57126/7056/2149/10544/5627

R-HSA-109582	Hemostasis	519	0.37687587	1.459240536	2.80E-05	0.0005528	0.00047178	2266/2244/5138/4312/5270/4846/5593/5672/3672/3798/5657/710/5669/6404/57126/7056/3678/960/2534/5271/5675/347733/7277/6546/5678/3690/11069/1608/3655/2149/3635/3689/23046/347688/54210/3673/3684/492/10544/5627/9162/8654/23764/440533/3827/5673/10381/5329/11343/7066/5026/6281/5152/5582/23533/5265/7280/6383/2335/3778/10846/3959/302/2161/3273/6401/7018/5676/6543/4099/7044/1794/2243/151/493/1048/5671/9127/2812/2993/5153/3046/4842/7409/87/7422/2786/135228/1950/5592/64780/12/2/5054/5768/3675/7040/3660/3688/308/88/6566/5336/2886/284/2152/22920/1609/634/7035/84876/3708/3797/5526/2817/30846/1/5345/5575
R-HSA-6803157	Antimicrobial peptides	31	0.713556079	1.990325147	3.25E-05	0.0006332	0.00054038	6278/6280/5266/51297/5657/3934/140683/92747/84659/338324/7097/8993/6279
R-HSA-202733	Cell surface interactions at the vascular wall	112	0.51952641	1.765730619	4.78E-05	0.0009213	0.00078624	4312/5672/5669/6404/57126/7056/3678/960/2534/5675/5678/3690/3655/3635/3689/54210/3684/10544/5627/440533/5673/6383/2335/6401/5676/4099/1048/5671/2993/3675/7040/3688/6566/2886/284/634
R-HSA-2408522	Selenoamino acid metabolism	114	0.510122385	1.734091878	5.15E-05	0.0009828	0.00083871	4143/118672/200916/1491/6176/6169/6189/6233/6232/3735/6207/6206/191/875/6170/6230/6155/51520/6158/6231/6165/9045/6217/6142/6157/7311/6144/6193/6125/6223/6218/6224/3176/6154/1615/51065/6141/6301/6234/6222/51121/6235/6147/6210/6152/6188/7296/25873/9521/6181/9349/6229/6173/3376/6135/6136/6167/6204/6124/6201/6161/6228/6175/6146/6156/6209/23521/11224/6159/6122/6132/6128/6191/6134/6227/6168/6143/6202/6194/6205/3921/6137/6160/6133/6203/6187/7965/6139/4736
R-HSA-5693538	Homology Directed Repair	136	-0.432939042	-1.731303771	5.70E-05	0.0010776	0.00091958	7156/9656/7158/144715/25/84142/116028/51426/51750/11073/6117/23514/8970/10524/83695/5892/5985/5883/8363/4683/5426/7515/6119/5888/146956/641/63967/7468/8914/4361/84464/2237/165918/8347/5427/8362/83990/8341/675/8370/554313/8339/80010/10721/3014/8367/8290/121504/8368/8348/8364/8366/3017/10635/348654/8343/8344/580/8360/8365/8346/8342/8340/8345/8361/8359/8294
R-HSA-8939236	RUNX1 regulates transcription of genes involved in differentiation of HSCs	127	-0.443012628	-1.762327826	6.62E-05	0.0012382	0.0010566	8347/3021/8362/8341/333932/126961/8370/554313/8339/8355/8334/3014/8367/121504/653604/8368/8348/8364/55766/8335/8968/8366/3017/474382/8343/8344/8354/3012/8360/8351/8365/94239/3015/8346/8358/8350/8357/8342/8331/8340/8345/2625/8361/8356/4602/8359/4004/8294/8352
R-HSA-5693607	Processing of DNA double-strand break ends	95	-0.472586657	-1.830414199	6.79E-05	0.0012581	0.00107359	9156/7156/9656/7158/144715/84142/116028/11073/6117/8970/10524/83695/5985/5883/8363/4683/6119/641/63967/7468/8914/4361/165918/8347/8362/83990/8341/8370/554313/8339/80010/3014/8367/8290/121504/8368/8348/8364/8366/3017/8343/8344/580/8360/8365/8346/8342/8340/8345/8361/8359/8294
R-HSA-1474244	Extracellular matrix organization	256	0.43287119	1.601488398	7.44E-05	0.0013648	0.0011647	2266/4322/2244/3371/55214/4312/1305/4314/3672/3908/3918/1520/140766/26254/284217/3909/3678/960/1306/3690/10536/3655/5650/3910/26577/3817/3689/3383/3673/3684/4017/340267/4319/1292/388743/3680/3914/3694/1282/4038/6383/2335/7148/2199/5644/170690/2201/2331/1311/1215/2243/1634/10319/8728/1463/1504/283208/7092/1290/1514/92291/87/85301/824/4053/10516/4146/11095/2/5054/3675/7040/2200/3688/5352/7077/8038/634/91522/9644/4326/4052/1299/5339/4148/1834/831/1285/147968/3915/8974/8754/79709/7087/8751/8425/4323/5045/4680/64386/7076/50859
R-HSA-72695	Formation of the ternary complex, and subsequently, the 43S complex	49	0.620810505	1.873763749	9.74E-05	0.0017678	0.00150861	6189/6233/6232/6207/6206/8664/6230/10480/8666/8663/6231/8667/6217/8668/6193/6223/6218/3646/6224/8894/51065/6234/6222/6235/6210/6188/51386/6229/8669/6204/8662/8661/6201/6228/1968/6209/6191/6227/27335/6202/6194/6205/3921/8665/6203/6187
R-HSA-8878171	Transcriptional regulation by RUNX1	223	-0.366429242	-1.562071005	0.0001085	0.0019512	0.00166505	8362/55193/8341/196528/57678/333932/126961/8370/554313/8339/8355/4773/8334/3014/8367/121504/653604/8368/8348/8364/8850/55766/8335/8968/8366/57332/3017/100532731/474382/8343/8344/8354/3012/8360/8351/8365/94239/3015/8346/8358/23466/8350/8357/8342/8331/8340/8345/2625/8361/8356/50943/4602/200845/8359/4004/8294/26053/1515/2099/8352/7122

R-HSA-198725	Nuclear Events (kinase and transcription factor activation)	59	0.58524731	1.81766728	0.0001198	0.0021338	0.00182088	1848/23237/1961/8061/6457/8941/3397/2354/3726/4066/429/10221/1958/6446/1849/4209/2152/1846/6197/7425/6196/4664
R-HSA-140877	Formation of Fibrin Clot (Clotting Cascade)	30	0.69423453	1.917903772	0.0001365	0.0024078	0.00205475	2266/2244/5270/5657/710/57126/7056/2149/10544/5627/3827/2161/2243/2812/2/2152/7035
R-HSA-5683057	MAPK family signaling cascades	292	0.412823208	1.539013744	0.000149	0.0026035	0.00222175	2266/3569/685/2244/374/5593/5698/5596/2323/2069/1848/1847/5696/1839/2534/2676/10602/25780/3690/4233/3568/2118/2064/57731/4609/377630/5159/1956/148170/2335/8437/3570/10642/9965/2251/3815/1844/2243/7039/122706/5156/9542/255324/8555/1950/8682/2906/1742/88/23179/221002/1849/284/9462/1843/1846/23198/5526/3725/5699/896/5058/58489/51114/9693/10434/2246/1852/5245/71/6233
R-HSA-9711097	Cellular response to starvation	152	0.469066741	1.644958488	0.0001685	0.0029171	0.00248937	58528/467/1051/83667/1649/90423/200916/245972/6176/6169/9296/6189/6233/526/6232/155066/6207/6206/6170/6230/6155/6158/6231/6165/9045/6217/6142/6009/6157/9550/7311/6144/533/6193/440/6125/6223/6218/6224/6154/8894/51065/6141/6234/6222/51121/6235/81929/6147/6210/6152/6188/25873/6181/9349/6229/6173/79726/6135/6136/6167/10670/6204/6124/6201/6161/527/6228/6175/6146/6156/1968/6209/23521/440275/11224/528/468/6159/10985/6122/6132/6128/6191/8131/6134/6227/6168/10312/6143/6202/6194/6205/10542/51606/3921/144577/6137/6160/57600/1054/6133/6203/523/6187/6139/4736/57761/6396/1965/8992/6208/2475/6129/51382
R-HSA-5693565	Recruitment and ATM-mediated phosphorylation of repair and signaling proteins at DNA double strand breaks	74	-0.488913975	-1.825711379	0.0001855	0.0031342	0.00267463	11200/8363/4683/7468/4361/165918/8347/8362/8341/8370/554313/8339/3014/8367/8290/121504/8368/8348/8364/8366/3017/8343/8344/580/8360/8365/8346/8342/8340/8345/8361/8359/8294
R-HSA-8941332	RUNX2 regulates genes involved in cell migration	8	0.888604625	1.794254638	0.0001829	0.0031342	0.00267463	4322/9358/3678
R-HSA-2559582	Senescence-Associated Secretory Phenotype (SASP)	107	-0.444383193	-1.709618785	0.000186	0.0031342	0.00267463	8347/3021/8362/8341/333932/126961/8370/554313/8339/8355/8334/3014/8367/121504/4790/653604/1031/8368/8348/8364/55766/8335/8968/8366/3017/474382/8343/8344/8354/3012/8360/8351/8365/94239/3015/8346/8358/8350/8357/8342/8331/8340/8345/8361/8356/8359/3552/8294/27287/8352
R-HSA-5693567	HDR through Homologous Recombination (HRR) or Single Strand Annealing (SSA)	130	-0.427565626	-1.710881995	0.0001947	0.0032504	0.0027738	9156/7156/9656/7158/144715/25/84142/116028/51426/51750/11073/6117/23514/8970/10524/83695/5892/5985/5883/8363/4683/5426/6119/5888/146956/641/63967/7468/8914/4361/84464/165918/8347/5427/8362/83990/8341/675/8370/554313/8339/80010/3014/8367/8290/121504/8368/8348/8364/8366/3017/10635/348654/8343/8344/580/8360/8365/8346/8342/8340/8345/8361/8359/8294
R-HSA-179812	GRB2 events in EGFR signaling	13	0.832843678	1.858561566	0.0002051	0.0033942	0.00289648	685/374/2069/1839/1956/7039/255324/1950
R-HSA-1236975	Antigen processing-Cross presentation	97	0.514231219	1.711118122	0.0002098	0.0034407	0.00293613	2266/2244/5698/6280/1520/5696/7097/6279/6891/6890/2243/4689/122706/1536/1514/9902/3106/3134/3105/23198/5699
R-HSA-72649	Translation initiation complex formation	56	0.588588281	1.809532486	0.0002359	0.0038366	0.00327404	26986/1974/6189/6233/6232/6207/6206/8664/6230/10480/8666/8663/6231/8667/6217/8668/6193/6223/6218/3646/6224/8894/51065/6234/6222/6235/6210/6188/51386/6229/8669/6204/8662/8661/6201/6228/1968/6209/6191/6227/27335/6202/6194/6205/3921/8665/1973/6203/1981/6187/1975/1965
R-HSA-449836	Other interleukin signaling	21	0.741901945	1.891214778	0.0002539	0.0040938	0.0034935	5657/1436/1440/282618/1435/6804
R-HSA-8875878	MET promotes cell motility	37	0.645420361	1.847819356	0.0002811	0.0044934	0.00383448	3908/3918/84951/284217/3909/3910/4233/3673/3914/2335/10319/1290/85301/3675/3688
R-HSA-180336	SHC1 events in EGFR signaling	14	0.808907403	1.841588177	0.0002842	0.0045034	0.003843	685/374/2069/1839/1956/7039/255324/1950
R-HSA-72702	Ribosomal scanning and start codon recognition	56	0.583536863	1.794002604	0.0002937	0.0046157	0.00393883	1974/6189/6233/6232/6207/6206/8664/6230/10480/8666/8663/6231/8667/6217/8668/6193/6223/6218/3646/6224/8894/51065/6234/6222/6235/6210/6188/51386/6229/8669/6204/8662/8661/6201/6228/1968/6209/6191/6227/27335/6202/6194/6205/3921/8665/1973/6203/1981/6187/1975/1965
R-HSA-5693606	DNA Double Strand Break Response	75	-0.486025371	-1.816793823	0.0003168	0.0049052	0.00418593	11200/8363/4683/7468/4361/165918/8347/8362/8341/8370/554313/8339/3014/8367/8290/121504/8368/8348/8364/8366/3017/8343/8344/580/8360/8365/8346/8342/8340/8345/8361/8359/8294

R-HSA-9010553	Regulation of expression of SLITs and ROBOs	165	0.447612319	1.578732238	0.0003174	0.0049052	0.00418593	5698/5696/3670/122706/64221/200916/26986/23198/5699/6176/6169/6189/6233/6232/6207/6206/6170/6230/6155/6158/6231/6165/2935/9045/6217/6142/2107/6157/7311/6144/6193/3199/6125/6223/6218/6224/6154/51065/6141/6234/6222/51121/6235/5690/5688/6147/6210/6152/6188/25873/6181/9349/6229/4440/6173/4116/6135/6136/6167/5692/6204/6124/6201/6161/5691/5700/6228/6175/6146/6156/6209/23521/11224/6159/6122/6132/6128/6191/5708/6134/6227/9491/6168/6143/6202/6194/6205/89884/10921/3921/6923/6137/6160/6133/6203/1981/6187/5682/5684/6921/6139/5704/4736/5718/5713/5719/5693/5695/5717/9861/5686/10197
R-HSA-6798695	Neutrophil degranulation	420	0.378678013	1.448632479	0.0003223	0.0049395	0.00421515	3310/6278/6280/6286/1520/5657/6317/6590/8972/3934/57126/960/978/7097/968/8993/5337/6279/23406/10326/3689/79895/3684/9961/2357/1116/5329/1823/25797/5799/5265/6282/11031/22875/11001/302/23601/11026/388697/2950/84658/1794/2512/10394/3579/1536/566/54509/2171/719/84418/353376/2040/3106/5836/1143/976/5724/12/6793/10875/11010/55313/5768/4311/966/272/7077/116844/4860/10493/25798/3105/634/1992/654364/127829/1265/1/6283/718/5912/6386/5236/8987/1476/728/11322/3163/201294/9588/2760/23593/4680/64386/199675/3683/83716/5315/5269/3240/3320/55276/240/8804/5660/4831/51209/1378/411/51719/929/1508/23250/64333/4048/1938/226/5328/56888/100302736/10043/3326/1828/121260/3107
R-HSA-72662	Activation of the mRNA upon binding of the cap-binding complex and eIFs, and subsequent binding to 43S	57	0.590137854	1.816219051	0.0003323	0.0050517	0.00431091	26986/1978/1974/6189/6233/6232/6207/6206/8664/6230/10480/8666/8663/6231/8667/6217/8668/6193/6223/6218/3646/6224/8894/51065/6234/6222/6235/6210/6188/51386/6229/8669/6204/8662/8661/6201/6228/1968/6209/6191/6227/27335/6202/6194/6205/3921/8665/1973/6203/1981/6187/1975/1965
R-HSA-5368286	Mitochondrial translation initiation	87	0.521064964	1.712009884	0.0003655	0.0055124	0.00470404	9553/64969/7818/51069/64979/90480/219927/55173/51073/64928/6183/4528/54460/64951/65080/51649/79590/51116/51373/10573/128308/65008/28973/64975/63875/118487/65005/64983/60488/64949/29074/122704/6182/28977/64960/51650/54148/54534/10884/51263/6150/64978/64981/26589/29088/28957/9801/64968/55052/51253/56945/124995/23107/54998/92259/29093/5018/65003/11222/3396/55037/54948
R-HSA-5419276	Mitochondrial translation termination	87	0.519535469	1.706984575	0.0003957	0.0059202	0.00505205	9553/64969/7818/51069/64979/90480/219927/55173/51073/64928/6183/54460/64951/65080/51649/79590/51116/51373/10573/128308/65008/28973/64975/63875/118487/65005/64983/60488/64949/29074/122704/84340/6182/28977/64960/51650/54148/54534/10884/51263/6150/64978/64981/26589/29088/28957/9801/64968/55052/51253/56945/124995/23107/54998/92259/29093/5018/65003/11222/3396/55037/54948
R-HSA-182971	EGFR downregulation	30	0.673681491	1.861123607	0.0004007	0.0059465	0.00507453	685/374/2069/1839/6457/10252/1956/7039/255324/1950
R-HSA-69306	DNA Replication	185	-0.368757031	-1.530104862	0.0004098	0.0060339	0.00514911	5422/5985/8363/23594/8353/84515/5426/5001/6119/4171/51659/9837/3020/8338/5000/2237/8347/5427/3021/8362/8341/333932/126961/3978/8370/554313/8339/8355/8334/8318/3014/8367/121504/653604/8368/8348/8364/55766/8335/8968/9134/8366/3017/474382/8343/8344/8354/3012/8360/8351/6502/8365/94239/3015/8346/8358/8350/8357/8342/8331/8340/8345/8361/8356/8359/8294/8352
R-HSA-201681	TCF dependent signaling in response to WNT	217	-0.359597718	-1.528667067	0.0004226	0.006174	0.00526864	8341/333932/126961/8321/8370/554313/83999/8339/8355/8334/3014/8367/8290/121504/653604/8368/8348/8364/55766/8335/8968/8366/3017/474382/8343/8344/6659/8354/3012/8360/6657/8351/8365/51176/94239/3015/8346/7088/8358/8350/8357/8342/8331/8340/8345/8361/8356/6658/8359/54361/8294/89780/83439/8313/8322/8352/54894
R-HSA-5638302	Signaling by Overexpressed Wild-Type EGFR in Cancer	8	0.873925501	1.764614812	0.0004314	0.0062054	0.00529548	685/374/2069/1839/1956/7039/255324/1950
R-HSA-5638303	Inhibition of Signaling by Overexpressed EGFR	8	0.873925501	1.764614812	0.0004314	0.0062054	0.00529548	685/374/2069/1839/1956/7039/255324/1950
R-HSA-1236974	ER-Phagosome pathway	85	0.525376165	1.722380031	0.0004423	0.0063139	0.00538802	2266/2244/5698/6280/5696/7097/6279/6891/6890/2243/122706/3106/3134/3105/23198/5699/6233/3133/23480

R-HSA-5693532	DNA Double-Strand Break Repair	165	-0.368783448	-1.497319771	0.0004903	0.006946	0.00592748	84142/116028/51426/51750/11073/6117/23514/8970/10524/83695/5892/5985/5883/11200/8363/4683/5426/7515/6119/5888/146956/641/63967/7468/8914/64421/4361/22976/84464/2237/165918/8347/5427/8362/83990/8341/675/8370/554313/8339/80010/10721/3014/8367/8290/121504/8368/8348/8364/8366/3017/10635/348654/8343/8344/580/8360/8365/8346/8342/8340/8345/8361/8359/8294
R-HSA-5368287	Mitochondrial translation	93	0.503075147	1.66447917	0.0005497	0.0077287	0.00659536	9553/64969/7818/51069/64979/90480/219927/55173/51073/64928/6183/4528/54460/64951/65080/51649/79590/51116/51373/10573/128308/65008/28973/64975/63875/118487/65005/64983/60488/64949/29074/122704/84340/7284/6182/28977/64960/51650/54148/54534/10884/51263/6150/64978/64981/26589/29088/28957/9801/64968/55052/51253/56945/124995/23107/54998/92259/29093/5018/65003/11222/3396/55037/54948
R-HSA-8939211	ESR-mediated signaling	213	-0.358780269	-1.519441843	0.000641	0.008945	0.00763337	8347/51523/3021/5885/8362/1509/8341/2771/57678/333932/126961/8370/554313/8339/8355/10499/8334/3014/8367/8503/121504/653604/8368/8348/8364/8850/55766/8335/2785/8968/8366/3017/474382/8343/8344/8354/3012/8360/8351/8365/94239/3015/8346/4318/8358/7031/8350/8357/8342/8331/8340/8345/2625/1903/8361/8356/5295/51764/4602/200845/8359/8294/9687/7033/2099/8352/2494/596/6387/2066
R-HSA-9006934	Signaling by Receptor Tyrosine Kinases	475	0.360937435	1.389124979	0.0006485	0.0089829	0.00766566	685/374/4846/3908/2323/3918/2069/1848/29126/84951/284217/3909/1839/2534/348/5125/23237/1961/8061/3690/9047/3910/6457/117/4233/2185/3673/1003/8941/3397/2064/10252/627/8877/4609/1292/639/5159/3914/2354/3091/1956/1282/2335/3726/9965/4908/2251/4066/9982/3815/429/1215/10319/7039/4689/1536/5156/9542/1290/10221/6776/9844/4914/7409/10810/255324/1958/85301/90423/7422/1950/6461/9306/1742/4486/6774/3675/3688/253260/6446/1849/4209/2886/8038/2152/3480/1846/53834/3708/6197/1299/7425/245972/6016/10019/6196/11140/5058/1285/3915/7006/10603/200958/2246/4664/5435/4145/5899/9296/2549/9564/71/581/6233/117145
R-HSA-5389840	Mitochondrial translation elongation	87	0.508760288	1.671581666	0.0006861	0.0094337	0.00805039	9553/64969/7818/51069/64979/90480/219927/55173/51073/64928/6183/54460/64951/65080/51649/79590/51116/51373/10573/128308/65008/28973/64975/63875/118487/65005/64983/60488/64949/29074/122704/7284/6182/28977/64960/51650/54148/54534/10884/51263/6150/64978/64981/26589/29088/28957/9801/64968/55052/51253/56945/124995/23107/54998/92259/29093/5018/65003/11222/3396/55037/54948
R-HSA-9735869	SARS-CoV-1 modulates host translation machinery	34	0.622495755	1.767315384	0.0007141	0.0097477	0.00831829	6189/6233/6232/6207/6206/6230/6231/6217/6193/6223/6218/6224/51065/6234/6222/6235/6210/6188/6229/6204/6201/6228/6209/6191/6227/6202/6194/6205/3921/6203/6187/1915
R-HSA-5607761	Dectin-1 mediated noncanonical NF-kB signaling	59	0.549795301	1.707560056	0.0007693	0.0104246	0.008896	5698/5696/5971/4791/122706/23198/9020/5699/6233/7311/5970/5690/5688/9040/5692/5691/1147/5700/5708/23291/9491/8945/5682/5684/5704/5718/5713/5719/5693/5695/5717/8454/9861/5686/10197/5714/5721/7316/5694/5687/5685
R-HSA-212718	EGFR interacts with phospholipase C-gamma	9	0.856814247	1.784462596	0.0008033	0.0108072	0.00922244	685/374/2069/1839/1956/7039/255324/1950
R-HSA-8874081	MET activates PTK2 signaling	26	0.685803456	1.83925422	0.0008354	0.0111582	0.00952203	3908/3918/284217/3909/3910/4233/3673/3914/2335/10319/1290/85301/3675/3688
R-HSA-5684996	MAPK1/MAPK3 signaling	254	0.400418869	1.482454022	0.0009136	0.0121165	0.01033977	2266/3569/685/2244/374/5593/5698/2323/2069/1848/1847/5696/1839/2534/2676/25780/3690/4233/3568/2064/57731/377630/5159/1956/2335/8437/3570/9965/2251/3815/1844/2243/7039/122706/5156/9542/255324/1950/8682/2906/1742/88/23179/221002/1849/284/9462/1843/1846/23198/5526/5699/58489/51114/9693/10434/2246/1852/5245/71/6233
R-HSA-73621	Pyrimidine catabolism	8	0.851533187	1.71940065	0.001048	0.0137183	0.01170669	4907/7378/1890
R-HSA-5676590	NIK-->noncanonical NF-kB signaling	58	0.548838858	1.70106404	0.001049	0.0137183	0.01170669	5698/5696/5971/4791/122706/23198/9020/5699/6233/7311/5690/5688/9040/5692/5691/1147/5700/5708/23291/9491/8945/5682/5684/5704/5718/5713/5719/5693/5695/5717/8454/9861/5686/10197/5714/5721/7316/5694/5687/5685
R-HSA-6783783	Interleukin-10 signaling	33	0.638119507	1.794193825	0.0010719	0.0139192	0.01187811	3569/7850/3576/3557/3606/942/3587/1440/3383/2357/414062/5743/1435/6349/3976/5724/6774
R-HSA-211916	Vitamins	6	0.881704182	1.652598958	0.001174	0.01514	0.01291991	1591/340665/1594/120227/56603

R-HSA-9609646	HCMV Infection	157	-0.368988135	-1.498427146	0.0012095	0.0154915	0.01321987	8347/440689/8362/317772/9611/8341/333932/126961/8370/554313/8330/8339/81027/8355/8332/79643/8334/8367/8336/79861/121504/4790/653604/8368/8348/8364/8335/8968/8366/8969/3017/8343/8344/7846/8354/3012/8360/8351/8365/8329/8346/85235/8358/8350/8357/8342/8331/8340/8345/113457/8361/8356/8359/8294/8352
R-HSA-9031628	NGF-stimulated transcription	38	0.614454991	1.773238403	0.0013972	0.0177742	0.01516788	23237/1961/8061/6457/8941/3397/2354/3726/4066/429/10221/1958/6446/4209/2152/7425/4664
R-HSA-157118	Signaling by NOTCH	228	-0.337343397	-1.439648114	0.0014114	0.0178328	0.01521785	9611/8341/23129/333932/126961/8370/554313/8339/8355/8334/23462/3014/8367/121504/6487/653604/8368/182/8348/1869/8364/8850/55766/8335/8968/8366/3017/474382/8343/8344/8354/3012/8360/8351/8365/94239/3015/3955/8346/408/7088/8358/8350/8357/8342/26960/8331/54492/8340/8345/83464/489/8361/8356/8359/4855/8294/23493/9148/8352/84441
R-HSA-174824	Plasma lipoprotein assembly, remodeling, and clearance	59	0.531405337	1.650444313	0.0018273	0.0229336	0.01957068	19/348/5125/26291/4864/338328/9388/8029/55908/341/2/7436/949/55911/5360/6233/5045/51129/9619/7376
R-HSA-450408	AUF1 (hnRNP D0) binds and destabilizes mRNA	55	0.539643858	1.647262751	0.0018453	0.023005	0.0196316	5698/5696/122706/26986/23198/5699/6233/7311/5690/5688/5692/3312/5691/5700/5708/9491/3315/1981/5682/5684/5704/5718/5713/5719/5693/5695/5717/9861/5686/10197/5714/3303/5721/7316/5694/5687/5685
R-HSA-351202	Metabolism of polyamines	58	0.538925038	1.670337277	0.0020062	0.0248453	0.02120207	5698/5696/6303/54498/122706/51686/79814/196743/6723/6611/23198/5699
R-HSA-211935	Fatty acids	13	-0.759392546	-1.915530676	0.0021578	0.0265471	0.02265429	1572/66002/11283/1549/1580/1573/126410
R-HSA-2559583	Cellular Senescence	190	-0.337412685	-1.404011034	0.0022181	0.0271099	0.0231346	23112/10524/79813/4194/57690/5594/29882/26277/23523/8363/4683/84733/8353/5600/2146/3006/3020/4361/8338/5595/7290/8347/3021/8362/8341/3008/333932/126961/5608/8370/554313/8339/25842/8355/5602/8334/3014/8367/8290/121504/4790/653604/1031/8368/8348/1869/8364/55766/8335/8968/9134/8366/57332/4001/3017/100532731/474382/8343/8344/1870/8354/3012/8360/8351/8365/94239/3015/3007/8346/8358/23466/8350/8357/8342/8331/8340/3009/8345/8361/8356/8359/3552/8294/27287/8352
R-HSA-373760	L1CAM interactions	106	0.466823859	1.578575039	0.0022346	0.0271345	0.02315563	1272/3672/6331/6329/284217/3678/347733/7277/3690/347688/3673/287/57731/3680/10381/286/1956/7280/3786/27255/1463/1641/1742/3688/6197/84617/6196/5058/6386/3915/6900/27330/71
R-HSA-5340588	Signaling by RNF43 mutants	8	-0.835786371	-1.787419126	0.002292	0.0276519	0.02359711	89780/8322/54894
R-HSA-112409	RAF-independent MAPK1/3 activation	23	0.673469001	1.747237452	0.0024215	0.0290274	0.02477089	3569/1848/1847/3570/1844/8682/1849/1843/1846/1852
R-HSA-1643713	Signaling by EGFR in Cancer	25	0.664937893	1.76398049	0.0027597	0.0328392	0.0280238	685/374/2069/1839/1956/7039/255324/1950/11140/2549/6233/3320/6464/867
R-HSA-180534	Vpu mediated degradation of CD4	50	0.543285027	1.644423162	0.0027976	0.0328392	0.0280238	5698/5696/122706/23198/5699/6233/7311/5690/5688/5692/5691/5700/5708/9491/8945/5682/5684/5704/5718/5713/5719/5693/5695/5717/9861/5686/10197/5714/5721/7316/5694/5687/5685
R-HSA-75815	Ubiquitin-dependent degradation of Cyclin D	51	0.541240812	1.640364545	0.0028098	0.0328392	0.0280238	5698/5696/122706/23198/5699/6233/7311/595/5690/5688/5692/5691/5700/5708/9491/5682/5684/5704/5718/5713/5719/5693/5695/5717/9861/5686/10197/5714/5721/2932/7316/5694/5687/5685
R-HSA-199418	Negative regulation of the PI3K/AKT network	94	0.469726121	1.552325126	0.002805	0.0328392	0.0280238	685/9173/374/2323/2069/90865/1839/2534/942/4233/8395/3556/2064/5159/1956/8870/9965/2251/3815/7039/5156/9542/7409/255324/1950
R-HSA-76009	Platelet Aggregation (Plug Formation)	35	0.59522114	1.703273797	0.0029267	0.0339937	0.02900901	2266/2244/3690/11069/7066/2335/2243/151/2812
R-HSA-5673001	RAF/MAP kinase cascade	248	0.387760813	1.431051849	0.0030211	0.0348728	0.0297592	2266/685/2244/374/5593/5698/2323/2069/1848/1847/5696/1839/2534/2676/25780/3690/4233/3568/2064/57731/377630/5159/1956/2335/8437/9965/2251/3815/1844/2243/7039/122706/5156/9542/255324/1950/8682/2906/1742/88/23179/221002/1849/284/9462/1843/1846/23198/5526/5699/58489/51114/9693/10434/2246/1852/5245/71/6233
R-HSA-6811558	PI5P, PP2A and IER3 Regulate PI3K/AKT Signaling	87	0.480316396	1.578126477	0.003213	0.0368608	0.03145562	685/9173/374/2323/2069/90865/1839/2534/942/4233/8395/3556/2064/5159/1956/8870/9965/2251/3815/7039/5156/9542/7409/255324/1950
R-HSA-446652	Interleukin-1 family signaling	139	0.426603596	1.48199839	0.0032556	0.0371217	0.0316783	9173/7850/3557/8809/3606/5698/5696/90865/3656/3556/84166/4791/8808/26525/122706/6774/64127/23198/6283/5699/4088/841/6233/57162/5782/11274/57161/240/351/8878/4792/79155/4615/7311/7335/5604/5970/3654/4793
R-HSA-8941858	Regulation of RUNX3 expression and activity	54	0.536785001	1.644540759	0.0032973	0.0373695	0.03188973	5698/5696/864/7040/5699/6233/57154/7311/5690/5688/64750/4193/6714/5692/5691/5700/5708/9491/5682/5684/5704/5718/5713/5719/5693/5695/5717/9861/5686/10197/5714/5721/7316/5694/5687/5685
R-HSA-156588	Glucuronidation	13	-0.745690972	-1.880969123	0.0033932	0.0382245	0.03261941	54577/54658/54578/54659/54579/54657

R-HSA-1474290	Collagen formation	76	0.489297818	1.575378339	0.0035688	0.0399616	0.03410174	4322/55214/1305/4314/3918/1520/140766/3909/1306/10536/3655/26577/4017/340267/1292/3914/1282/283208/7092/1290/1514/85301/5352/91522/1299/5339/1285/8974/79709
R-HSA-8848021	Signaling by PTK6	51	0.534175081	1.618950094	0.0038801	0.0429341	0.03663835	685/2069/120892/1839/2064/2034/3091/1956/9542/9844/898/1950/6774/10656/9021/10457/55620/9564/6233
R-HSA-9006927	Signaling by Non-Receptor Tyrosine Kinases	51	0.534175081	1.618950094	0.0038801	0.0429341	0.03663835	685/2069/120892/1839/2064/2034/3091/1956/9542/9844/898/1950/6774/10656/9021/10457/55620/9564/6233
R-HSA-180292	GAB1 signalosome	17	0.704177364	1.692945524	0.0041117	0.0452292	0.03859691	685/374/2069/1839/1956/7039/255324/1950
R-HSA-211733	Regulation of activated PAK-2p34 by proteasome mediated degradation	49	0.542920617	1.638672289	0.0045355	0.0495984	0.04232542	5698/5696/122706/23198/5699/6233/7311/5690/5688/5692/5691/5700/5708/9491/5682/5684/5704/5718/5713/5719/5693/5695/5717/9861/5686/10197/5714/5721/7316/5694/5687/5685
R-HSA-216083	Integrin cell surface interactions	73	0.493610586	1.579439096	0.0047049	0.0499892	0.04265898	2266/2244/3371/1305/3672/3678/960/3690/3655/3689/3383/3673/3684/1292/3680/3694/1282/2335/1311/2243/1290/3675/2200/3688
R-HSA-168179	Toll Like Receptor TLR1:TLR2 Cascade	107	0.447756166	1.514773437	0.0046653	0.0499892	0.04265898	2266/2244/6280/1848/7097/6279/3656/84166/2243/4791/1849/64127/1846/6197/3725/6283/6196/841/6233/57162/11274/57161/7867/351/929/6271/4792/79155/4615/7311/7335/51295/5516/5604/5970/3654/4793
R-HSA-181438	Toll Like Receptor 2 (TLR2) Cascade	107	0.447756166	1.514773437	0.0046653	0.0499892	0.04265898	2266/2244/6280/1848/7097/6279/3656/84166/2243/4791/1849/64127/1846/6197/3725/6283/6196/841/6233/57162/11274/57161/7867/351/929/6271/4792/79155/4615/7311/7335/51295/5516/5604/5970/3654/4793
R-HSA-166016	Toll Like Receptor 4 (TLR4) Cascade	135	0.432424764	1.496299063	0.0046995	0.0499892	0.04265898	2266/2244/6280/1848/7097/6279/3689/3684/3656/84166/330/2243/4791/3929/353376/3665/1849/5336/64127/148022/1846/6197/3725/6283/6196/841/6233/57162/7187/11274/57161/7867/351/929/6271/4792/100302736/79155/4615/7311/7335/51295/5516/5604/5970/3654/4793
R-HSA-1632852	Macroautophagy	131	0.427700488	1.477960365	0.0046528	0.0499892	0.04265898	5563/100526767/123/285973/347733/7277/58528/23710/1780/347688/2034/10381/7280/65018/10226/55062/5565/84617/81631/60673/6233/11152/84557/3320/9821/51422/8878/7416/54543/9927/6009/7311/7335/22863/112714/10382/25978/5289/11345/1457/10452/8678/10533/6714/10670/84790/3312/4077/5830/9776/3297
R-HSA-354192	Integrin signaling	26	0.640077507	1.716621936	0.0048492	0.0506589	0.04323044	2266/2244/3690/11069/2335/2243
R-HSA-8854691	Interleukin-20 family signaling	19	0.674452323	1.671425977	0.0048319	0.0506589	0.04323044	53832/6775/282618/11009/58985/6776/6774
R-HSA-418346	Platelet homeostasis	68	0.502328347	1.589993806	0.0048008	0.0506589	0.04323044	5138/4846/5593/6546/492/8654/5026/5152/3778/10846/6543/493/9127/5153/4842/2786/5592/84876/3708/5526
R-HSA-3000157	Laminin interactions	28	0.61163407	1.657144581	0.0048791	0.0506879	0.04325523	3672/3908/3918/284217/3909/3655/3910/3673/3914/1282/10319/3675/3688/1285/3915
R-HSA-6785807	Interleukin-4 and Interleukin-13 signaling	90	0.462473397	1.526084988	0.0049168	0.050798	0.04334916	3569/4312/3576/3606/4314/3934/6097/301/3689/3383/3684/4609/1026/3091/3566/5292/2335/3570/3726/5743/356/3605/3662/3598/3929/3976/4582/7422/6774/7040/3688/9021/6624/10538/1052/79923/6095/604
R-HSA-1266695	Interleukin-7 signaling	33	-0.545688127	-1.72939372	0.0051217	0.0526238	0.0449072	333932/126961/8355/8503/653604/64109/8968/8354/8351/8835/8358/8350/8357/1154/8356/5295/8352
R-HSA-75205	Dissolution of Fibrin Clot	12	0.746543336	1.663743475	0.0053652	0.0548246	0.04678533	5270/5271/5329/6281/302/3273/5054/5345
R-HSA-391160	Signal regulatory protein family interactions	12	0.74532064	1.661018579	0.0054265	0.0551498	0.04706284	2533/10326/2185
R-HSA-349425	Autodegradation of the E3 ubiquitin ligase COP1	51	0.523128715	1.585471342	0.0055397	0.0556952	0.04752823	5698/5696/122706/23198/5699/6233/7311/5690/5688/5692/5691/5700/5708/9491/5682/5684/5704/5718/5713/5719/5693/5695/5717/9861/5686/10197/5714/5721/7316/5694/5687/5685
R-HSA-1234174	Cellular response to hypoxia	73	0.490293573	1.568825426	0.005512	0.0556952	0.04752823	768/5698/5696/2034/3091/122706/7422/126374/23198/25994/5699/112399/6233/7311/7321/5690/5688/5692/5691/5700/5708/9491/6923/5682/5684/6921/8994/5704/54583/5718/5713/5719/5693/5695/5717/9861/5686/10197/5714/405/5721
R-HSA-4420097	VEGFA-VEGFR2 Pathway	93	0.458799895	1.517989655	0.0056001	0.0560009	0.04778914	4846/2534/3690/9047/2185/1003/8877/4689/1536/9844/7409/10810/7422/6461/253260/3708/5058/9564/71/117145/3320/5829/7867/3710/5578/5590/25759/10000
R-HSA-5687128	MAPK6/MAPK4 signaling	87	0.469115225	1.541323936	0.005669	0.0563886	0.04811994	5698/5596/5696/10602/2118/4609/148170/10642/122706/8555/23198/3725/5699/896/5058/6233/192670/7311/2308/5690/5688/3337/5692/5597/5691/5700/5708/9491/3315/10435/5682/5684/8550/5704/5718/5713/5719/5693/5695/5717/9861/5686/10197/5879/5714
R-HSA-5603041	IRAK4 deficiency (TLR2/4)	15	0.708364038	1.641122251	0.0057658	0.0570475	0.04868225	2266/2244/6280/7097/6279

R-HSA-169911	Regulation of Apoptosis	52	0.532045022	1.618662702	0.005812	0.0572027	0.04881465	5698/5696/122706/23198/5699/6233/7311/5690/5688/5692/5691/5700/5708/9491/4976/5682/5684/5704/79658/5718/5713/5719/5693/5695/5717/9861/5686/10197/5714/5721/7316/5694/5687/5685
R-HSA-166058	MyD88:MAL(TIRAP) cascade initiated on plasma membrane	106	0.449312975	1.519361606	0.0060341	0.0587695	0.0501517	2266/2244/6280/1848/7097/6279/3656/84166/2243/4791/1849/64127/1846/6197/3725/6283/6196/841/6233/57162/11274/57161/7867/351/929/6271/4792/79155/4615/7311/7335/51295/5516/5604/5970/3654/4793
R-HSA-168188	Toll Like Receptor TLR6:TLR2 Cascade	106	0.449312975	1.519361606	0.0060341	0.0587695	0.0501517	2266/2244/6280/1848/7097/6279/3656/84166/2243/4791/1849/64127/1846/6197/3725/6283/6196/841/6233/57162/11274/57161/7867/351/929/6271/4792/79155/4615/7311/7335/51295/5516/5604/5970/3654/4793
R-HSA-8857538	PTK6 promotes HIF1A stabilization	6	0.841206312	1.576692845	0.0060972	0.0590769	0.05041409	120892/1839/3091/1956
R-HSA-174184	Cdc20:Phospho-APC/C mediated degradation of Cyclin A	72	0.482031759	1.54484969	0.0061779	0.0594534	0.05073537	5698/5696/8900/122706/23198/5699/7324/6233/7311/10393/7321/5690/5688/246184/5692/27338/5691/5700/4085/5708/9491/5682/5684/5704/5718/5713/5719/5693/5695/5717/9861/5686/10197/64682/5714/5721/51433/7316/890/5694/5687/5685/9184
R-HSA-195721	Signaling by WNT	306	-0.294274029	-1.296876082	0.0061997	0.0594534	0.05073537	333932/126961/8321/8370/554313/83999/8339/8355/8334/3014/8367/8290/121504/51384/653604/8368/8348/8364/55766/8335/2785/8968/8366/3017/23002/474382/8343/5148/8344/6659/8354/3012/8360/6657/4920/8351/8365/51176/94239/3015/8346/7088/3709/8358/8350/8357/8342/8331/8340/5530/8345/2775/8361/8356/6658/50855/51764/8359/54361/8294/89780/5330/83439/8313/8322/8352/54894
R-HSA-9759194	Nuclear events mediated by NFE2L2	76	0.474167609	1.526664032	0.0062699	0.0598201	0.0510483	5698/5696/4609/5699/4851/7975/6233/23657/8878/4097/2730/7311/4780/140809/5690/5688/7296/5692/5691/5700/3162/468/5226/5708/9491/8945/5682/5684/5704/5718/5713/5719/5693/5695/5717/8454/9861/5686/10197/5714/5721/2932/7316/5694/7086/5687/5685
R-HSA-166520	Signaling by NTRKs	126	0.43260388	1.490104817	0.0063058	0.0598567	0.05107948	1848/2534/5125/23237/1961/8061/6457/117/8941/3397/627/2354/3726/4908/4066/429/10221/4914/1958/6774/6446/1849/4209/2152/1846/6197/7425/6016/6196/4664/5899/2549/581/5045
R-HSA-354194	GRB2:SOS provides linkage to MAPK signaling for Integrins	15	0.705939715	1.635505633	0.0065607	0.0619625	0.05287651	2266/2244/3690/2335/2243
R-HSA-9762114	GSK3B and BTRC:CUL1-mediated-degradation of NFE2L2	52	0.528996154	1.609387004	0.0068784	0.0646366	0.05515846	5698/5696/5699/6233/7311/4780/5690/5688/5692/5691/5700/5708/9491/8945/5682/5684/5704/5718/5713/5719/5693/5695/5717/8454/9861/5686/10197/5714/5721/2932/7316/5694/5687/5685
R-HSA-392154	Nitric oxide stimulates guanylate cyclase	17	0.685041664	1.6469405	0.0069561	0.0647163	0.05522648	5138/4846/5593/8654/5152/3778/10846/5153/4842/5592/3708
R-HSA-3000178	ECM proteoglycans	66	0.500551108	1.5791719	0.0069399	0.0647163	0.05522648	3371/3908/284217/3909/3690/3910/3673/1292/3680/3694/1282/4038/2335/7148/2331/1311/1634/1463/1290/4146/5054/7040/3688/1299/4148/1834/1285/3915/3381/1280/63827/1277/351/3693
R-HSA-6806834	Signaling by MET	74	0.47299636	1.514573781	0.0071204	0.0659169	0.05625103	3908/3918/84951/284217/3909/3910/6457/4233/3673/3914/2335/10319/1290/85301/6774/3675/3688/3915/200958/2549/6233/1280/6464/1277/867
R-HSA-8963898	Plasma lipoprotein assembly	12	0.733397478	1.634446668	0.007456	0.0686831	0.05861165	19/348/341/2
R-HSA-2179392	EGFR Transactivation by Gastrin	9	0.794406855	1.654488501	0.0075992	0.0693197	0.05915488	4314/1839/1956
R-HSA-9012546	Interleukin-18 signaling	6	0.8365974	1.568054253	0.0075863	0.0693197	0.05915488	8809/3606
R-HSA-8854050	FBXL7 down-regulates AURKA during mitotic entry and in early mitosis	53	0.522889494	1.596188216	0.0077935	0.0707472	0.06037303	5698/5696/122706/23198/5699/6233/7311/5690/5688/5692/5691/5700/5708/9491/5682/5684/5704/5718/5713/5719/5693/5695/5717/8454/9861/5686/10197/5714/5721/7316/5694/5687/5685
R-HSA-168898	Toll-like Receptor Cascades	155	0.401543379	1.404045365	0.0079059	0.0714208	0.06094793	2266/2244/6280/1520/1848/7097/6279/3689/3684/3656/84166/330/2243/4791/3929/1514/353376/3665/1687/1849/5336/64127/148022/1846/6197/3725/6283/6196/841/6233/57162/7187/11274/57161/7867/351/929/1508/6271/4792/100302736/79155/4615/7311/7335/51295/5516/5604/5970/3654/4793
R-HSA-196791	Vitamin D (calciferol) metabolism	11	0.753996093	1.668440056	0.0079839	0.0714349	0.06095992	1591/1594/120227/8029/7421
R-HSA-9754678	SARS-CoV-2 modulates host translation machinery	48	0.524959685	1.577049339	0.0079753	0.0714349	0.06095992	6606/6189/6233/6232/6207/6206/79833/6636/6230/8487/6231/6217/6193/6223/6218/6607/6224/51065/6234/6222/6235/6210/6188/6229/6204/6201/6228/6209/25929/6191/6227/6202/6194/6205/6637/6633/3921/6203/6187/6632
R-HSA-4641263	Regulation of FZD by ubiquitination	17	-0.650324528	-1.755190134	0.0083231	0.0741149	0.06324692	89780/8322/54894
R-HSA-9755511	KEAP1-NFE2L2 pathway	98	0.445839965	1.488237569	0.0085281	0.0755808	0.06449792	5698/5696/4609/83667/122706/23198/5699/4851/7975/81631/6233/23657/8878/4097/2730/7311/4780/10000/140809/5690/1457/5688/7296/5692/5691/5700/3162/468/5226/5708/1459/9491/8945/5682/5684/5704/55666/1460/5718/5713/5719/5693/5695/5717/8454/9861/5686/10197/26043/5714/5721/2932/7316/27244/5694/7086/5687/5685
R-HSA-5686938	Regulation of TLR by endogenous ligand	15	0.698935814	1.619279148	0.0088907	0.0784223	0.06692271	2266/2244/6280/7097/6279/2243/3929/1687

R-HSA-6790901	rRNA modification in the nucleus and cytosol	61	0.499967906	1.546650476	0.0089764	0.0788071	0.06725109	100861532/55505/9136/27292/23160/51202/2091/9277/25879/65083/134430/55813/115939/84916/56902/10607/51118/114049/6201/79050/4809/11103/55127/27042/57647/84128/10199/6194/11056/10171/8602/22984/10885/92856/9790/54555/6203/4839/6187/5822/6208/55651/51602/55226
R-HSA-5602498	MyD88 deficiency (TLR2/4)	14	0.717071182	1.632510478	0.00907	0.0792562	0.06763435	2266/2244/6280/7097/6279
R-HSA-1257604	PIP3 activates AKT signaling	246	0.373802668	1.380050995	0.0095365	0.082945	0.07078218	685/9173/374/5698/2323/2069/5696/90865/1839/2534/58528/942/4233/6591/8395/3556/2064/5159/1026/1956/8870/57167/9965/2251/3815/7039/122706/5156/9542/6615/7409/255324/1958/1950/3164/253260/10014/23198/5526/3725/5468/5699
R-HSA-69601	Ubiquitin Mediated Degradation of Phosphorylated Cdc25A	51	0.503232027	1.525169493	0.0097348	0.0835047	0.07125983	5698/5696/122706/23198/5699/6233/7311/5690/5688/5692/5691/5700/5708/9491/5682/5684/5704/5718/5713/5719/5693/5695/5717/9861/5686/10197/5714/5721/7316/5694/5687/5685
R-HSA-69610	p53-Independent DNA Damage Response	51	0.503232027	1.525169493	0.0097348	0.0835047	0.07125983	5698/5696/122706/23198/5699/6233/7311/5690/5688/5692/5691/5700/5708/9491/5682/5684/5704/5718/5713/5719/5693/5695/5717/9861/5686/10197/5714/5721/7316/5694/5687/5685
R-HSA-69613	p53-Independent G1/S DNA damage checkpoint	51	0.503232027	1.525169493	0.0097348	0.0835047	0.07125983	5698/5696/122706/23198/5699/6233/7311/5690/5688/5692/5691/5700/5708/9491/5682/5684/5704/5718/5713/5719/5693/5695/5717/9861/5686/10197/5714/5721/7316/5694/5687/5685
R-HSA-936440	Negative regulators of DDX58/IFIH1 signaling	35	0.551898894	1.579303658	0.0100604	0.0859039	0.07330727	51191/84166/7128/9246/54941/64135/9636/7318/1540/6233/7187/83737/7311/7321/23586/84282/79671/55593
R-HSA-5610780	Degradation of GLI1 by the proteasome	58	0.501725796	1.555042426	0.0101691	0.0862611	0.07361208	5698/5696/122706/2735/23198/5699/6233/83737/7311/5690/5688/5692/5691/5700/5708/9491/8945/5682/5684/5704/5718/5713/5719/5693/5695/5717/8454/9861/5686/10197/5714/5721/7316/5694/5687/5685
R-HSA-177929	Signaling by EGFR	49	0.514838772	1.553914149	0.0101945	0.0862611	0.07361208	685/374/2069/1839/6457/10252/1956/7039/255324/1950/8038/2549/6233/5782/6464/867/5829
R-HSA-179419	APC:Cdc20 mediated degradation of cell cycle proteins prior to satisfation of the cell cycle checkpoint	73	0.474221295	1.517397875	0.0102653	0.0864688	0.07378934	5698/5696/8900/122706/23198/5699/7324/6233/7311/10393/7321/5690/5688/246184/5692/27338/5691/5700/4085/5708/9491/5682/5684/5704/5718/5713/5719/5693/5695/5717/9861/5686/10197/64682/5714/5721/51433/7316/890/5694/5687/5685/9184
R-HSA-4641258	Degradation of DVL	54	0.506228238	1.550924429	0.0109756	0.0920373	0.07854128	5698/5696/122706/23198/5699/6233/1855/7311/5690/5688/5692/5691/5700/5708/9491/5682/5684/5704/5718/5713/5719/5693/5695/5717/9861/5686/10197/5714/5721/7316/5694/5687/5685
R-HSA-174113	SCF-beta-TrCP mediated degradation of Emi1	54	0.503871391	1.543703791	0.0116149	0.0969637	0.08274531	5698/5696/122706/23198/5699/6233/7311/5690/5688/5692/5691/5700/5708/9491/8945/5682/5684/5704/5718/5713/5719/5693/5695/5717/8454/9861/5686/10197/5714/5721/7316/5694/5687/5685
R-HSA-418457	cGMP effects	13	0.733157696	1.636103812	0.0118821	0.0987534	0.08427256	5138/5593/8654/5152/3778/10846/5153/5592/3708
R-HSA-1280218	Adaptive Immune System	667	0.318159445	1.244630516	0.0120903	0.1000391	0.08536967	2266/2244/2533/5698/6280/1520/3798/29126/51191/8519/5696/10870/2534/11119/347733/10666/7277/25780/7097/22914/11069/942/221687/6279/131405/3635/3689/1780/347688/4345/54210/3383/80380/1519/114907/51676/353514/7498/153579/6891/10381/6890/64167/55008/7280/9246/11275/259197/11006/11026/3820/3115/2243/390594/3119/7535/10859/4689/122706/1536/1510/1514/7409/80176/26232/9902/3106/10437/57630/127544/26249/5592/10385/7318/100302652/5817/6494/3134/3688/253260/912/57823/3105/5336/9021/26091/22920/51088/79176/84876/10384/3708/23198/3797/5526/8764/9020/7220/114088/5699/718/130340/5493/8915/84617/7454/5058/151636/7328/26270/7087/9903/147700/7324/6233/117145/64219/100507436/3133/2280/23480/4008/55585/285852/3111/974/3683/997/51465/93611/64837/8722/51725/1280/1277/114876/83737/51752/55294/929/1508/6271/57154/3693/10484/26191/27071
R-HSA-211859	Biological oxidations	168	-0.325052135	-1.32001681	0.0121774	0.1003159	0.08560594	9420/2687/6916/54996/22977/219970/348158/246181/1588/51000/128/29785/5740/2052/1572/2944/10861/6256/64757/57491/66002/54577/54658/54905/2939/1577/6820/9060/1589/11283/2678/1549/54578/1580/1573/1548/126410/54659/54579/84532/54657/4129

R-HSA-9612973	Autophagy	145	0.406117442	1.417791042	0.0124827	0.1023803	0.08736758	5563/100526767/123/285973/347733/7277/58528/23710/1780/347688/2034/10381/7280/65018/10226/55062/5565/84617/81631/137492/60673/6233/11152/84557/3320/9821/51422/8878/7416/54543/51271/79720/9927/3326/6009/7311/7335/22863/112714/10382/25978/5289/11345/1457/10452/8678/10533/6714/10670/84790/3312/4077/5830/9776/3297
R-HSA-3000171	Non-integrin membrane-ECM interactions	54	0.501286772	1.535785328	0.0131067	0.1070285	0.09133416	3371/3908/3918/284217/3909/3690/3655/3910/3673/3914/1282/6383/2335/10319/1290/87/7040/3688
R-HSA-2871837	FCERI mediated NF-kB activation	76	0.455347087	1.466068132	0.0132066	0.1073756	0.09163042	5698/5696/2206/122706/23198/5699/8915/6233/997/4792/7311/7335/5970/7321/4067/5690/5688/5692/5691/1147/5700/5708/23291/9491/8945/5682/5684/5704/5718/5713/5719/5693/5695/5717/8454/9861/5686/10197/5714/8517/5721/7316/5694
R-HSA-8953854	Metabolism of RNA	665	0.310615107	1.215139267	0.0133105	0.107752	0.09195157	5698/10644/100861532/5696/102800317/27350/10642/403314/115024/122706/134637/164668/55505/10799/200916/23246/7538/29974/26986/23198/9136/55621/5699/28987/6606/9188/11097/1974/1022/5435/27292/6176/27238/6169/6189/6233/54512/54974/8568/6232/81890/6207/6206/4904/79833/23160/285755/6636/90353/6170/26354/51202/2091/6230/8780/11260/6155/8487/2068/9277/5546/6158/56915/1207/24140/5393/84321/10969/25879/6231/6165/2935/9045/5356/5438/11102/54802/65083/55131/6217/6142/10557/2107/55720/6157/5578/134430/2966/55178/7311/55813/115939/25949/6144/6193/5520/6627/6125/6223/6218/103/6607/6224/84916/91801/113179/83480/8896/6154/11157/84967/9129/51065/56902/8125/134353/55759/80746/6141/10285/6234/6222/51639/51121/10907/9092/6235/54888/5690/81929/5688/2963/6147/6210/6152/29107/5437/6188/25873/6181/9349/6229/4691/6173/10607/51118/4116/6135/705/6136/114049/10914/23016/6829/6167/5692/10940/6204/84705/26168/3312/6124/6201/79050/6161/5691/7534/5700/348180/6228/6175/10946/6146/6156/53981/10450/116461/9261/6209/23521/100534599/4809/57418/79922/51637/1
R-HSA-69017	CDK-mediated phosphorylation and removal of Cdc6	72	0.460707545	1.476508334	0.0134263	0.108221	0.09235182	5698/5696/8900/122706/898/23198/5699/7324/6233/7311/10393/7321/5690/5688/246184/5692/27338/5691/5700/5708/9491/5682/5684/5704/5718/5713/5719/5693/5695/5717/9861/5686/10197/64682/5714/5721/51433/7316/890/5694
R-HSA-1059683	Interleukin-6 signaling	11	0.728769887	1.612619589	0.0136337	0.1094204	0.09337539	3569/3570/6774/9021/867/6772
R-HSA-446107	Type I hemidesmosome assembly	9	0.773582266	1.611117723	0.0138304	0.1105246	0.09431766	3918/3909/3655/3914
R-HSA-8963899	Plasma lipoprotein remodeling	22	0.605701684	1.564798824	0.0139744	0.1112005	0.09489443	348/5125/26291/338328/9388/55908/5360/5045/51129/9619
R-HSA-372708	p130Cas linkage to MAPK signaling for integrins	15	0.685255168	1.587584128	0.0141721	0.1118222	0.09542501	2266/2244/3690/2335/2243
R-HSA-9006925	Intracellular signaling by second messengers	283	0.360477788	1.34184854	0.0141382	0.1118222	0.09542501	685/9173/374/5698/2323/2069/5696/90865/1839/2534/58528/942/107/4233/6591/8395/3556/2064/5159/1026/1956/5582/8870/57167/9965/2251/3815/108/7039/122706/5156/5153/9542/6615/7409/255324/1958/113/1950/3164/253260/10014/3708/23198/5526/3725/5468/5699/5575
R-HSA-350562	Regulation of ornithine decarboxylase (ODC)	50	0.498247228	1.508102086	0.0145303	0.1141663	0.09742533	5698/5696/122706/51686/23198/5699/4953/5690/5688/5692/5691/5700/5708/9491/5682/5684/5704/5718/5713/5719/5693/5695/5717/9861/5686/10197/5714/5721/5694/5687/5685
R-HSA-5610785	GLI3 is processed to GLI3R by the proteasome	58	0.49285236	1.527540214	0.0146288	0.1144594	0.09767547	5698/5696/122706/23198/5699/6233/7311/5690/5688/5692/5691/5700/5708/9491/8945/5682/5684/5704/5718/5713/5719/5693/5695/5717/8454/9861/5686/10197/5714/5721/2932/7316/5694/5687/5685
R-HSA-180585	Vif-mediated degradation of APOBEC3G	53	0.500289456	1.527198659	0.0150247	0.1170675	0.09990111	5698/5696/122706/23198/5699/6233/7311/5690/5688/5692/5691/5700/5708/9491/6923/5682/5684/6921/5704/5718/5713/5719/5693/5695/5717/9861/5686/10197/5714/5721/7316/5694/5687/5685
R-HSA-189085	Digestion of dietary carbohydrate	5	0.839882637	1.488675105	0.0150969	0.1171416	0.09996433	8972/280
R-HSA-5610783	Degradation of GLI2 by the proteasome	58	0.492156595	1.52538377	0.0152513	0.1178507	0.10056943	5698/5696/122706/23198/5699/6233/7311/5690/5688/5692/5691/5700/5708/9491/8945/5682/5684/5704/5718/5713/5719/5693/5695/5717/8454/9861/5686/10197/5714/5721/2932/7316/5694/5687/5685
R-HSA-176814	Activation of APC/C and APC/C:Cdc20 mediated degradation of mitotic proteins	76	0.451402729	1.453368593	0.0154245	0.1186988	0.1012932	5698/5696/8900/122706/23198/5699/7324/6233/7311/10393/7321/5690/5688/246184/5692/27338/5691/5700/4085/5708/9491/5682/5684/5704/5718/5713/5719/5693/5695/5717/9861/5686/10197/64682/5714/5721/51433/7316/890/5694/5687/5685/9184
R-HSA-1169410	Antiviral mechanism by IFN-stimulated genes	79	0.447132128	1.448637011	0.0158373	0.1211783	0.10340915	402569/51191/8638/9246/4938/9636/7318/6059/3434/11097/1974/7324/6233/11274/4599/6772

R-HSA-418555	G alpha (s) signalling events	104	0.416975996	1.406928869	0.0158763	0.1211783	0.10340915	5138/9340/3678/3274/3640/3363/107/117/10267/2869/2863/5141/2335/10846/136/108/5153/117579/4159/27115/113/2786/3688/285601/7253/554/133/5575/1081/6019/11245
R-HSA-1474151	Tetrahydrobiopterin (BH4) synthesis, recycling, salvage and regulation	12	0.700990765	1.562225198	0.0159763	0.1214462	0.10363774	4846/5593
R-HSA-77111	Synthesis of Ketone Bodies	8	-0.765812921	-1.63777337	0.016153	0.1219343	0.10405425	79611/56898/54511
R-HSA-1234176	Oxygen-dependent proline hydroxylation of Hypoxia-inducible Factor Alpha	65	0.476604618	1.495376255	0.016171	0.1219343	0.10405425	5698/5696/2034/3091/122706/126374/23198/5699/112399/6233/7311/7321/5690/5688/5692/5691/5700/5708/9491/6923/5682/5684/6921/8994/5704/54583/5718/5713/5719/5693/5695/5717/9861/5686/10197/5714/5721/7316/5694
R-HSA-9725371	Nuclear events stimulated by ALK signaling in cancer	19	0.628772473	1.558222293	0.0164802	0.122781	0.10477677	597/1051/2335/3726/6776/6774
R-HSA-176408	Regulation of APC/C activators between G1/S and early anaphase	80	0.442943403	1.438914024	0.0164788	0.122781	0.10477677	5698/5696/8900/122706/23198/5699/7324/6233/7311/10393/7321/5690/5688/246184/5692/27338/5691/5700/4085/5708/9491/8945/5682/5684/5704/5718/5713/5719/5693/5695/5717/8454/9861/5686/10197/64682/5714/5721/51433/7316/890/5694/5687/5685/9184
R-HSA-9663891	Selective autophagy	76	0.44679694	1.438539465	0.0164326	0.122781	0.10477677	5563/123/347733/7277/1780/347688/2034/10381/7280/65018/10226/5565/84617/81631/6233/84557/3320/51422/8878/7416/54543/9927/7311/7335/112714/10382/1457/10452/6714/84790/3312/4077/5830/3297
R-HSA-964739	N-glycan trimming and elongation in the cis-Golgi	5	-0.827897393	-1.603821631	0.0165505	0.122815	0.10480584	10905/57134/79694/4121
R-HSA-1566977	Fibronectin matrix formation	6	0.800751279	1.500867022	0.0168113	0.1242578	0.10603703	3678/2335/3688/634/4680
R-HSA-1169091	Activation of NF-kappaB in B cells	64	0.480333463	1.497802104	0.0172624	0.1270892	0.1084533	5698/5696/122706/23198/5699/8915/6233/4792/7311/5970/4794/4793/5690/5688/5692/5691/1147/5700/5708/23291/9491/8945/5682/5684/5704/5718/5713/5719/5693/5695/5717/8454/9861/5686/10197/5714/8517/5721/7316/5694/5687/5685
R-HSA-432047	Passive transport by Aquaporins	10	0.727306405	1.549127484	0.0174502	0.1279678	0.10920307	364/360/358/361/359
R-HSA-877300	Interferon gamma signaling	79	0.444277026	1.439386934	0.0176301	0.1287827	0.10989846	2635/23650/960/8638/3383/11074/3394/3115/10107/3119/4938/3662/6672/4261/3106/10437/3665/5724/3460/3660/3134/9830/3105/9021/3459/53840/4502/10379
R-HSA-174154	APC/C:Cdc20 mediated degradation of Securin	67	0.477942086	1.513643093	0.0177761	0.1293439	0.11037735	5698/5696/122706/23198/5699/7324/6233/7311/10393/7321/5690/5688/246184/5692/27338/5691/5700/5708/9491/5682/5684/5704/5718/5713/5719/5693/5695/5717/9861/5686/10197/64682/5714/5721/51433/7316/5694/5687/5685
R-HSA-447041	CHL1 interactions	8	0.753831372	1.522122883	0.0183318	0.1318483	0.11251451	3672/3673/286/27255/3688
R-HSA-69541	Stabilization of p53	55	0.482304609	1.472234705	0.0182159	0.1318483	0.11251451	5698/5696/122706/23198/5699/6233/7311/5690/5688/4193/5692/5691/5700/5708/9491/5682/5684/5704/5718/5713/5719/5693/5695/5717/9861/5686/10197/5714/5721/7316/5694/5687/5685
R-HSA-176409	APC/C:Cdc20 mediated degradation of mitotic proteins	75	0.452779052	1.452159836	0.0182966	0.1318483	0.11251451	5698/5696/8900/122706/23198/5699/7324/6233/7311/10393/7321/5690/5688/246184/5692/27338/5691/5700/4085/5708/9491/5682/5684/5704/5718/5713/5719/5693/5695/5717/9861/5686/10197/64682/5714/5721/51433/7316/890/5694/5687/5685/9184
R-HSA-9634638	Estrogen-dependent nuclear events downstream of ESR-membrane signaling	24	0.598411302	1.577684498	0.0184998	0.1325468	0.11311055	685/374/2069/1839/1956/7039/255324/1950
R-HSA-196807	Nicotinate metabolism	29	0.590953843	1.611221011	0.0187009	0.1329694	0.11347125	4907/1582/160728/23057/9390/5743/27231/349565/10135/128240/55328/143/54625/23475
R-HSA-8939256	RUNX1 regulates transcription of genes involved in WNT signaling	5	-0.822500649	-1.593366937	0.0187011	0.1329694	0.11347125	50943/2099
R-HSA-163125	Post-translational modification: synthesis of GPI-anchored proteins	66	0.469085946	1.479903514	0.0189075	0.1339284	0.11428959	22854/80328/7070/5329/1368/54742/338328/53942/4045/1048/137797/154064/83639/8000/135228/10232/266727/8581/116372
R-HSA-2162123	Synthesis of Prostaglandins (PG) and Thromboxanes (TX)	14	0.691165998	1.573533789	0.0189838	0.1339613	0.11431763	3248/9536/1582/5743
R-HSA-210990	PECAM1 interactions	11	0.710864034	1.572997578	0.0196661	0.1377561	0.11755597	2534/3690/3635
R-HSA-8963743	Digestion and absorption	12	0.693621023	1.545801022	0.0196917	0.1377561	0.11755597	8972/29881/5407/280
R-HSA-9692914	SARS-CoV-1-host interactions	92	0.440747857	1.457333338	0.0197426	0.1377561	0.11755597	114548/3656/64135/11123/2810/4088/6189/6233/2280/7187/4869/6232/6207/6206/83737/6230/9111/4792/6231/6217/7311/861/6193/6223/6218/5970/6224/51065/6234/6222/6235/6210/6188/6229/23586/6204/6201/7534/6228/7532/684/6209/6191/3661/6227/6202/6194/6205/9868/3921/6203/6187/7706/857
R-HSA-194138	Signaling by VEGF	102	0.420772754	1.412719692	0.0200861	0.1396322	0.11915702	4846/2534/3690/9047/2185/1003/8877/4689/1536/9844/7409/10810/7422/6461/253260/3708/5058/9564/71/117145/3320/8828/5829/7867/3710/5578/5590/25759/10000
R-HSA-112411	MAPK1 (ERK2) activation	9	0.755873476	1.574236133	0.0203245	0.1407658	0.12012434	3569/3570
R-HSA-8951671	RUNX3 regulates YAP1-mediated transcription	8	0.745155031	1.504603771	0.0205464	0.1417778	0.12098795	25937/864/1490/8463/7004
R-HSA-2426168	Activation of gene expression by SREBF (SREBP)	42	-0.45731571	-1.518798378	0.0207915	0.1429419	0.12198136	80205/8648/6604/6319/5465/1595/6720/3422/9453/6713/6721/6907/3157/1717/57678/4598/10499/7108/3156/6256/4047/79071/32

R-HSA-162909	Host Interactions of HIV factors	126	0.407000346	1.401913399	0.0208763	0.1429985	0.1220297	5698/5696/2534/3159/3055/1794/122706/9844/3105/23198/5699/130340/291/11097/6233/4869/7311/5690/81929/5688/5692/5691/1174/5700/5708/567/9491/8815/9818/51606/8945/6923/5905/55690/5682/5684/6921/5704/5718/5713/5719/160/5693/5695/5717/9861/6396/5686/10197/5901/1175/5879/1173/5714
R-HSA-5693537	Resolution of D-Loop Structures	35	-0.478783442	-1.531334549	0.0221018	0.1508405	0.12872175	7979/1763/7516/672/9156/7156/116028/51750/23514/10524/5892/4683/5888/146956/641/4361/84464/83990/675/80010/10635/348654/580
R-HSA-417957	P2Y receptors	9	-0.735686463	-1.630438877	0.0222444	0.151262	0.12908142	27334/5031/5029/10161/9934/53829/2846/64805
R-HSA-5260271	Diseases of Immune System	28	0.560005929	1.517264711	0.0227062	0.1532871	0.13080958	2266/2244/6280/7097/6279/2243/4791
R-HSA-5602358	Diseases associated with the TLR signaling cascade	28	0.560005929	1.517264711	0.0227062	0.1532871	0.13080958	2266/2244/6280/7097/6279/2243/4791
R-HSA-9705683	SARS-CoV-2-host interactions	179	0.379597657	1.352484339	0.0228075	0.1534171	0.13092057	114548/7097/3656/3605/64135/9636/3106/3665/3134/3105/64127/2810/6606/11097/81631/6189/6233/3133/7187/6232/6207/6206/3320/79833/6636/6230/6772/8487/10484/6231/6217/3326/3107/7311/7335/22863/6193/10000/7555/6223/6218/3654/6607/6224/51065/6234/5289/6222/6235/81929/6210/6188/6229/8678/23586/92359/2697/84282/6204/6201/7534/1147/6228/7532/6209/10802/25929/7284/6191/3661/567/6227/5781/6202/6194/6205/9868/7082/6637/6633/9818/89122/3921
R-HSA-446353	Cell-extracellular matrix interactions	18	0.627351776	1.535093484	0.0230802	0.1535943	0.13107173	29780/55679/87/9459/3688/71/5829/6251/7408/55742/60/2316
R-HSA-174084	Autodegradation of Cdh1 by Cdh1:APC/C	64	0.472454323	1.473232937	0.0229201	0.1535943	0.13107173	5698/5696/5699/7324/6233/7311/10393/7321/5690/5688/246184/5692/27338/5691/5700/5708/9491/5682/5684/5704/5718/5713/5719/5693/5695/5717/9861/5686/10197/64682/5714/5721/51433/7316/5694/5687/5685
R-HSA-168928	DDX58/IFIH1-mediated induction of interferon-alpha/beta	66	0.463365262	1.461855519	0.0230505	0.1535943	0.13107173	51191/84166/7128/9246/54941/4791/64135/9636/3665/7318/79132/1540/6283/841/6233/7187/3320/83737/351/4792/843/3326/7311/5970/4793/7321/23586/84282/79671/55593/1147
R-HSA-9701898	STAT3 nuclear events downstream of ALK signaling	9	0.749997357	1.561998107	0.0232108	0.1539153	0.13134565	29126/639/3091/6774
R-HSA-181431	Acetylcholine binding and downstream events	12	0.688177439	1.533669472	0.0239025	0.1562853	0.13336818	1139/1135/89832/1143/1145
R-HSA-622327	Postsynaptic nicotinic acetylcholine receptors	12	0.688177439	1.533669472	0.0239025	0.1562853	0.13336818	1139/1135/89832/1143/1145
R-HSA-9675136	Diseases of DNA Double-Strand Break Repair	41	-0.44167276	-1.461206125	0.0238014	0.1562853	0.13336818	7979/1763/7516/5984/672/9156/7156/144715/116028/11073/6117/10524/83695/5892/5985/5883/4683/6119/5888/641/4361/83990/675/80010/10635/580
R-HSA-9701190	Defective homologous recombination repair (HRR) due to BRCA2 loss of function	41	-0.44167276	-1.461206125	0.0238014	0.1562853	0.13336818	7979/1763/7516/5984/672/9156/7156/144715/116028/11073/6117/10524/83695/5892/5985/5883/4683/6119/5888/641/4361/83990/675/80010/10635/580
R-HSA-71291	Metabolism of amino acids and derivatives	341	0.339287673	1.284487757	0.0241688	0.157476	0.13438422	5698/5696/1734/1735/6303/374569/5053/4143/54498/144193/635/50506/2731/1158/29958/51151/1428/122706/15/1373/586/118672/1152/89874/339983/120103/51686/162417/8564/200916/259307/347735/1491/79814/196743/5860/6723/6611/23198/58472/2744/5699/1081/27165/2628/57571/6176/6169/4507/6189/6233/130013/29968/6232/3735/6207/6206/191/10003/875/123688/6170/6230/6155/8604/51520/6158/2184/6231/6165/9045/6217/8140/6142/6157/7311/4953/84706/6144/4552/6193/440/51166/6125/6223/6218/100131187/6224/3176/3081/435/6154/38/1615/4357/51065/6141/6301/6234/6222/51268/51121/384/9673/6235/5690/5688/6147/6210/6152/6188/7296/25873/9521/6181/9349/6229/6173/3376/4706/6135/6136/6167/2806/5692/6204/2746/80150/6124/6201/5160/6161/2875/5691/5700/6228/6175/6146/6156/6520/6209/23521/11224/6159/6122/6132/6128/6191/5708/2805/262/2639/138199/6134/6227/6535/1738/9491/6168/6143/6202/6194/6205/11019/84890/5092
R-HSA-6783589	Interleukin-6 family signaling	21	0.611189262	1.558009347	0.024705	0.1598558	0.13641509	3569/3589/9180/1271/3570/3976/6774/9021
R-HSA-168638	NOD1/2 Signaling Pathway	35	0.517256212	1.480170802	0.0246507	0.1598558	0.13641509	837/3656/7128/330/64170/1540/64127/842/841/6233/83737/7311/7335/3654
R-HSA-110056	MAPK3 (ERK1) activation	10	0.712236988	1.517030353	0.0257528	0.1660614	0.1417107	3569/3570
R-HSA-2187338	Visual phototransduction	78	0.435122667	1.402096022	0.0269489	0.1725839	0.14727676	64220/348/57016/83875/5949/50700/611/121214/6010/6383/9249/2979/338328/2978/101060233/4035/10170
R-HSA-187037	Signaling by NTRK1 (TRKA)	109	0.410637979	1.389522398	0.0268897	0.1725839	0.14727676	1848/23237/1961/8061/6457/117/8941/3397/2354/3726/4066/429/10221/4914/1958/6774/6446/1849/4209/2152/1846/6197/7425/6016/6196/4664/5899
R-HSA-6802948	Signaling by high-kinase activity BRAF mutants	35	0.512417436	1.466324249	0.0277598	0.1771698	0.15119019	2266/2244/3690/2335/2243

R-HSA-195258	RHO GTPase Effectors	313	-0.271543374	-1.193299091	0.0284041	0.1806653	0.15417314	3020/220134/8338/5063/5595/79172/808/10403/55143/64105/8347/3021/25936/4629/8362/995/644150/8341/55166/333932/126961/57082/147841/9735/8370/554313/57405/11113/378708/81930/8339/54908/81027/8355/10499/8334/79019/3014/8367/79861/121504/653604/8368/8348/8364/151648/55766/1062/8335/8968/124056/8366/1063/3017/23002/474382/2491/8343/8344/7846/8354/3012/8360/8351/8365/94239/3015/8346/100526739/8358/8350/8357/8342/11130/8331/8340/8345/113457/8361/8356/388/8359/367/8294/51466/7456/8352
R-HSA-1236978	Cross-presentation of soluble exogenous antigens (endosomes)	47	0.486468724	1.459482138	0.0298947	0.189206	0.16146149	5698/5696/122706/9902/23198/5699/5690/5688/5692/5691/5700/5708/9491/5682/5684/5704/5718/5713/5719/5693/5695/5717/9861/5686/10197/5714/5721/5694/5687/5685
R-HSA-9006931	Signaling by Nuclear Receptors	279	-0.280159206	-1.221363501	0.0299492	0.189206	0.16146149	9611/8341/2771/57678/333932/126961/5164/5165/8370/554313/10062/8339/8355/10499/8334/3014/8367/8503/121504/653604/8368/8348/8364/8850/55766/8335/8608/2785/8968/8366/3017/474382/8343/5914/8344/10123/8354/6256/3012/8360/8351/8365/94239/3015/8346/4318/8358/7031/8350/8357/8342/8331/8340/8345/2625/1903/8361/8356/5295/51764/4602/200845/8359/8294/9687/54659/7033/2099/5959/8352/2494/596/6387/2066
R-HSA-8935964	RUNX1 regulates expression of components of tight junctions	5	-0.798881558	-1.547611497	0.0309475	0.1948546	0.1662818	100506658/7122
R-HSA-8956321	Nucleotide salvage	22	0.569763119	1.471953408	0.0313911	0.1969842	0.16809906	270/978/100/7378/1890/272/4860/7371/8226/271
R-HSA-5693579	Homologous DNA Pairing and Strand Exchange	43	-0.443939119	-1.49434701	0.0320588	0.2005015	0.17110062	7979/1111/1763/7516/5984/672/9156/7156/144715/116028/11073/6117/10524/83695/5892/5985/5883/4683/6119/5888/641/4361/83990/675/80010/10635/580
R-HSA-389887	Beta-oxidation of pristanoyl-CoA	9	-0.719786118	-1.595200304	0.0328742	0.2049161	0.17486792	54677/1384/8309
R-HSA-8851680	Butyrophilin (BTN) family interactions	9	0.736209021	1.533281532	0.0335983	0.207565	0.17712834	11119/7498/153579/10385/10384/5493
R-HSA-419037	NCAM1 interactions	35	-0.461789615	-1.47698172	0.0338343	0.207565	0.17712834	776/784/131873/8913/4684/1298/1287/4902/1281/8911/7903/2674
R-HSA-9709570	Impaired BRCA2 binding to RAD51	35	-0.461077531	-1.474704202	0.0338343	0.207565	0.17712834	7979/1763/5984/672/9156/7156/144715/116028/11073/6117/10524/83695/5985/5883/4683/6119/5888/641/4361/83990/675/80010/580
R-HSA-69563	p53-Dependent G1 DNA Damage Response	64	0.463453525	1.445166157	0.0338542	0.207565	0.17712834	5698/5696/8900/1026/122706/898/57060/23198/5699/6233/7311/5690/5688/4193/5692/5691/5700/5708/9491/5682/5684/5704/5718/5713/5719/5693/5695/5717/9861/5686/10197/5714/5721/7316/890/5694
R-HSA-69580	p53-Dependent G1/S DNA damage checkpoint	64	0.463453525	1.445166157	0.0338542	0.207565	0.17712834	5698/5696/8900/1026/122706/898/57060/23198/5699/6233/7311/5690/5688/4193/5692/5691/5700/5708/9491/5682/5684/5704/5718/5713/5719/5693/5695/5717/9861/5686/10197/5714/5721/7316/890/5694
R-HSA-5693568	Resolution of D-loop Structures through Holliday Junction Intermediates	34	-0.473680951	-1.513826391	0.0344371	0.2090824	0.17842327	7979/1763/7516/672/9156/7156/116028/23514/10524/5892/4683/5888/146956/641/4361/84464/83990/675/80010/10635/348654/580
R-HSA-381426	Regulation of Insulin-like Growth Factor (IGF) transport and uptake by Insulin-like Growth Factor Binding Proteins (IGFBPs)	100	0.421585809	1.410211692	0.0343915	0.2090824	0.17842327	2266/3569/3371/4312/7857/354/348/3817/3827/3486/51280/5265/6383/2335/7018/2243/1356/1435/60676/10232/5768/3956/10272/2200/3895/54587/8404/3489/147111/4052/718/7425/4148/3491/3915
R-HSA-73894	DNA Repair	333	-0.27183331	-1.204480766	0.0344147	0.2090824	0.17842327	8554/672/11284/79184/5430/57461/54617/93973/723790/8337/9156/353497/11201/7156/9656/124739/7158/144715/25/55120/4292/25913/51455/84142/116028/51426/51750/2189/2968/11073/6117/23514/8970/2187/9768/10524/57697/83695/5892/3150/5985/5883/26277/23583/11200/8363/5980/4683/5426/7515/6119/5888/146956/641/63967/7468/8914/64421/4361/22976/8338/2073/84464/7398/2237/165918/2175/9937/8347/5427/8362/51514/83990/8341/675/3978/55215/79661/8370/554313/378708/8339/7374/80010/8334/10721/3014/8367/2176/8290/121504/8368/8348/8364/55766/8335/8366/2177/3017/91442/10635/474382/348654/8343/8344/580/3012/8360/8365/94239/3015/8346/100526739/8342/8331/8340/8345/8361/8359/8294
R-HSA-5693616	Presynaptic phase of homologous DNA pairing and strand exchange	40	-0.44293477	-1.455612161	0.0346817	0.2094185	0.17871009	7979/1111/1763/7516/5984/672/9156/7156/144715/116028/11073/6117/10524/83695/5892/5985/5883/4683/6119/5888/641/4361/83990/675/80010/580
R-HSA-9027307	Biosynthesis of maresin-like SPMs	5	0.81763838	1.449247607	0.0347164	0.2094185	0.17871009	1571/1559/1565
R-HSA-379716	Cytosolic tRNA aminoacylation	24	0.570552409	1.504235779	0.034873	0.2096863	0.17893858	7453/3735/7407/8565/51520/4677/2617/1615/6301/2193/3035/9521/3376/10056/5464/7965/6897/2058/5917/4141
R-HSA-74182	Ketone body metabolism	10	-0.691107594	-1.602953713	0.035575	0.212765	0.18156585	79611/56898/54511

R-HSA-5218859	Regulated Necrosis	54	0.477574618	1.463138732	0.0356125	0.212765	0.18156585	100526767/3606/837/330/197259/356/838/355/1687/3660/11140/6386/841/581/6233/57162/3320/8797/54205/7311/8795/25978
R-HSA-69615	G1/S DNA Damage Checkpoints	66	0.450906956	1.422551227	0.0359116	0.2134297	0.1821331	5698/5696/8900/1026/122706/898/57060/23198/5699/6233/7311/5690/5688/4193/5692/5691/5700/5708/9491/5682/5684/5704/5718/5713/5719/5693/5695/5717/9861/5686/10197/5714/5721/7316/890/5694
R-HSA-611105	Respiratory electron transport	93	0.414407702	1.371113226	0.0359521	0.2134297	0.1821331	9997/91942/10131/93058/10128/29078/2109/4714/54205/7381/4728/6390/51295/4717/4725/7388/2110/2108/80219/9377/4715/4726/4702/7386/10975/4706/7384/4723/1327/84987/1537/51079/4716/4731/137682/126328/51300/4713/1337/54539/285521/4701/4724/6391/374291/55967/4708/25880/1350/7385/1329
R-HSA-1655829	Regulation of cholesterol biosynthesis by SREBP (SREBF)	55	-0.404632618	-1.43137633	0.0361875	0.2141478	0.18274591	80205/8648/6604/6319/10427/5465/1595/6720/3422/9453/6713/6721/6907/3157/8720/1717/57678/4598/3638/10499/7108/3156/6256/4047/79071/32
R-HSA-9668328	Sealing of the nuclear envelope (NE) by ESCRT-III	30	0.545240273	1.50628978	0.036345	0.2144008	0.1829618	100526767/347733/7277/347688/10381/7280
R-HSA-9619483	Activation of AMPK downstream of NMDARs	27	0.549147721	1.484647899	0.0368905	0.2164233	0.18468774	5563/347733/7277/347688/10381/7280/4137/5565/84617
R-HSA-9675135	Diseases of DNA repair	51	-0.411334443	-1.427027569	0.0369193	0.2164233	0.18468774	7979/1763/4436/7516/5984/55247/672/9156/7156/144715/4292/116028/11073/6117/10524/83695/5892/5985/5883/4683/6119/5888/641/4361/83990/675/79661/80010/10635/580
R-HSA-187577	SCF(Skp2)-mediated degradation of p27/p21	60	0.460939921	1.429178939	0.0377095	0.2196784	0.18746548	5698/5696/8900/1026/898/5699/1163/6233/7311/595/5690/5688/5692/5691/5700/5708/9491/5682/5684/5704/5718/5713/5753/5719/5693/5695/5717/8454/9861/5686/10197/5714/5721/7316/890/5694/5687/5685
R-HSA-2219530	Constitutive Signaling by Aberrant PI3K in Cancer	60	0.459490852	1.424685992	0.0377095	0.2196784	0.18746548	685/374/2323/2069/1839/2534/942/4233/2064/5159/1956/9965/2251/3815/7039/5156/9542/7409/255324/1950
R-HSA-156711	Polo-like kinase mediated events	16	-0.597419393	-1.575296048	0.0380854	0.221179	0.18874602	2033/9133/7465/993/9088/5928/891/91750/4605/2305/995/1063
R-HSA-1474228	Degradation of the extracellular matrix	116	0.398502032	1.36024026	0.0399485	0.2312805	0.19736632	4322/4312/1305/4314/3918/1520/26254/3909/960/1306/5650/3817/4319/1292/388743/3914/1282/2335/5644/170690/2201/1215/1634/1504/7092/1290/1514/92291/824/11095/2/2200/7077/91522/4326/1299/831/1285/147968/3915/8754/8751/4323/5045/64386/7076/50859
R-HSA-176974	Unwinding of DNA	12	-0.619636119	-1.530056155	0.04042	0.2332885	0.19907983	84296/4176/4174/4175/4172/84515/4171/51659/9837/8318
R-HSA-372790	Signaling by GPCR	495	0.312878735	1.208591647	0.0407725	0.2345989	0.20019809	5138/1238/3576/9340/4314/30817/5997/1839/3678/1325/4168/23620/3814/3274/2015/4886/2693/1608/2149/3640/3363/107/7471/117/301/885/10267/2150/611/146/9718/185/221472/147/50649/7480/134/9162/2357/7481/51083/3827/9628/2869/11343/3269/10636/1956/1132/2863/6010/5582/23533/8326/5141/2335/10846/4828/1902/136/623/3549/9560/414062/1268/5733/11211/338398/84658/108/80834/151/3356/339403/2911/3579/27199/6866/101060233/5153/5999/6349/117579/4159/27115/2828/719/85397/23596/7409/113/2786/9181/976/5724/8601/79948/5142/9459/165140/3688/285601/7349/58191/7253/1609/554/7473/54429/3708/6197/133/8786/5731/718/5575
R-HSA-176187	Activation of ATR in response to replication stress	37	-0.459105729	-1.507174767	0.0416318	0.2384257	0.20346379	1111/5984/4176/55388/4174/993/4175/144715/990/4998/4172/6117/5985/5883/23594/84515/5001/6119/4171/63967/5000/995/8318
R-HSA-975163	IRAK2 mediated activation of TAK1 complex upon TLR7/8 or 9 stimulation	14	0.648426709	1.47623196	0.0416926	0.2384257	0.20346379	3656/353376/148022/6233/929/100302736/7311
R-HSA-9648895	Response of EIF2AK1 (HRI) to heme deficiency	15	0.629857396	1.459239787	0.0428336	0.2442038	0.20839458	467/23645/1051/79094/1649
R-HSA-380994	ATF4 activates genes in response to endoplasmic reticulum stress	25	0.540500651	1.433867153	0.0430108	0.2444684	0.20862038	3576/467/1051/1649/22926/54512/56915/5393/440
R-HSA-8957275	Post-translational protein phosphorylation	86	0.424634566	1.396642765	0.043364	0.2449869	0.20906286	2266/3569/3371/7857/348/3827/3486/51280/5265/6383/2335/7018/2243/1356/1435/10232/5768/3956/10272/2200/3895/54587/8404/147111/4052/718/7425/4148/3491/3915
R-HSA-2219528	PI3K/AKT Signaling in Cancer	86	0.424128181	1.394977241	0.043364	0.2449869	0.20906286	685/374/2323/2069/1839/2534/942/4233/2064/5159/1026/1956/9965/2251/3815/7039/5156/9542/7409/255324/1950/3164/253260
R-HSA-5624138	Trafficking of myristoylated proteins to the cilium	5	-0.784547813	-1.51984384	0.0435739	0.2454315	0.20944223	403/6102/27031
R-HSA-8931987	RUNX1 regulates estrogen receptor mediated transcription	6	-0.750883432	-1.485320466	0.0439097	0.24658	0.21042236	57678/200845/2099
R-HSA-9018678	Biosynthesis of specialized proresolving mediators (SPMs)	18	0.5853729	1.432373604	0.0443038	0.2480482	0.21167525	3248/241/1543/1571/1559/5743
R-HSA-190828	Gap junction trafficking	40	0.495858487	1.440597295	0.0446429	0.2484588	0.2120256	2707/347733/7277/2700/347688/10381/7280/375519/2702/2709/2706/84617/1212/71/2701/127534
R-HSA-8852276	The role of GTSE1 in G2/M progression after G2 checkpoint	75	0.429081324	1.376156125	0.0445946	0.2484588	0.2120256	5698/5696/347733/7277/347688/10381/1026/7280/122706/23198/5699/84617/6233/3320/3326/7311/112714/10382/5690/5688/5692/84790/5691/5700/5708/9491/22919/5682/5684/5704/5718/5713/5719/5693/5695/5717/9861/5686/10197

R-HSA-444821	Relaxin receptors	5	0.803249776	1.423744094	0.0454545	0.2514301	0.21456124	3640/339403/117579/6019
R-HSA-5674135	MAP2K and MAPK activation	39	0.489893574	1.418618107	0.0453172	0.2514301	0.21456124	2266/2244/3690/2335/2243
R-HSA-5658442	Regulation of RAS by GAPs	66	0.44499797	1.403909168	0.0455801	0.2514301	0.21456124	5698/5696/8437/122706/9462/23198/5699/6233/22821/7311/200734/5690/5688/5692/5691/5700/5708/9491/161742/5682/5684/5704/5718/5713/5719/5693/5695/5717/9861/5686/10197/5714/5721/7316/5694/5687/5685/3845
R-HSA-9613354	Lipophagy	8	0.738550255	1.491267525	0.0460526	0.2532895	0.21614796	5563/123/10226/5565
R-HSA-389359	CD28 dependent Vav1 pathway	11	0.673914062	1.491234804	0.0467742	0.2561668	0.21860336	2534/942/7409/5058
R-HSA-881907	Gastrin-CREB signalling pathway via PKC and MAPK	16	0.617157671	1.458624066	0.0468498	0.2561668	0.21860336	4314/1839/1956/6197/6196
R-HSA-1234158	Regulation of gene expression by Hypoxia-inducible Factor	10	0.70167073	1.494524735	0.0473083	0.2564248	0.21882353	768/2034/3091/7422/25994
R-HSA-8849473	PTK6 Expression	5	0.801562831	1.420754018	0.0472727	0.2564248	0.21882353	2034/3091
R-HSA-157858	Gap junction trafficking and regulation	42	0.484058105	1.419441517	0.0471976	0.2564248	0.21882353	2707/347733/7277/2700/347688/10381/7280/375519/2702/2709/2706/84617/1212/71/2701/127534
R-HSA-4641265	Repression of WNT target genes	12	-0.609830091	-1.505842311	0.047975	0.2572629	0.21953877	7089/6932/3065/51176/7088/83439
R-HSA-193775	Synthesis of bile acids and bile salts via 24-hydroxycholesterol	12	0.650623702	1.449977364	0.0480132	0.2572629	0.21953877	1582/11001/10998/23600/10858/1645/6718/1593
R-HSA-450531	Regulation of mRNA stability by proteins that bind AU-rich elements	87	0.415983845	1.366755593	0.0476821	0.2572629	0.21953877	5698/5696/122706/7538/26986/23198/5699/6233/54512/56915/5393/5578/7311/8125/5690/5688/23016/5692/3312/5691/7534/5700/9261/5708/9491/3315/8570/1981/5682/5684/5704/5718/5713/5719/5693/5695/5717/9861/5686/10197/5714/3303/5721/7316/5694/677/51010/5687/5685
R-HSA-9678108	SARS-CoV-1 Infection	138	0.376980976	1.306206087	0.0479798	0.2572629	0.21953877	100526767/114548/3656/10610/1514/64135/11123/2810/4088/143/81631/54625/6189/6233/2280/7187/4869/6232/6207/6206/83737/6230/9111/4792/6231/6217/84875/7311/861/6193/6223/6218/5970/6224/25978/51065/6234/5289/6222/6235/6210/6188/6229/8678/23586/6204/6201/7534/6228/7532/684/6209/128866/6480/6191/3661/6227/7341/6202/6194/6205/9868
R-HSA-8963889	Assembly of active LPL and LIPC lipase complexes	14	0.643240436	1.464424701	0.0482623	0.2575758	0.21980571	5125/26291/338328/55908/5045/51129
R-HSA-5218920	VEGFR2 mediated vascular permeability	29	0.511851299	1.395549883	0.0484848	0.2575758	0.21980571	4846/1003/7409/253260/5058/117145/3320
R-HSA-5357956	TNFR1-induced NFkappaB signaling pathway	29	0.511554649	1.394741076	0.0484848	0.2575758	0.21980571	7128/330/9099/7185/1540/6233/7132/10616/7311/27005/10399/1147
R-HSA-69481	G2/M Checkpoints	164	-0.300237983	-1.216973002	0.0489409	0.2592622	0.2212449	4174/7157/672/79184/993/9156/4175/7156/9656/9088/7158/144715/990/84142/4998/4172/116028/7533/11073/6117/983/8970/10524/891/83695/5985/5883/11200/8363/23594/4683/84515/5001/6119/4171/641/63967/7468/4361/5000/165918/8347/51512/8362/83990/995/8341/8370/554313/8339/80010/8318/3014/8367/8290/121504/8368/8348/8364/8366/3017/8343/8344/580/8360/8365/8346/8342/8340/8345/8361/8359/8294
R-HSA-983170	Antigen Presentation: Folding, assembly and peptide loading of class I MHC	28	0.511516403	1.385888518	0.0493066	0.2604616	0.22226835	6891/6890/64167/3106/3134/3105/3133/51752/10484/3107/22863/5289/8678/10802/567/10134/9871/6396/9632/22872/3309/6892
