# Supplementary material for: Activation of invasion by oncogenic reprogramming of cholesterol metabolism via increased NPC1 expression and macropinocytosis
Source: Oncogene. 2023 Jul 7;42(33):2495–506. doi: 10.1038/s41388-023-02771-x (PMC10421736; doi:10.1038/s41388-023-02771-x)
Supplement: Supplementary file 4 — Supplementary Table 3 [file 41388_2023_2771_MOESM4_ESM.pdf]

Supplementary Table 3: Antibodies and fluorescent dyes/compounds used in the study.

**Antibody/dye**	Brand	Cat No	Working concentration
Primary antibodies			
Alfa-tubulin	Abcam	Ab18251	1,43 µg/mL
Alfa-tubulin	Abcam	Ab15246	0,59 µg/mL
Beta-actin HRP (Loading Control)	Abcam	Ab20272	0,1 µg/mL
EGFR (1005)	Santa Cruz Biotechnologies	sc-03	0,4 µ/mL
Fascin-1	Santa Cruz Biotechnologies	sc-21743	0,2 µg/mL
Fibronectin	Abcam	Ab2413	0,3 µg/mL
Lamp-2 (H4B4-c)	Developmental Studies Hybridoma Bank	Ab528129	1,22 µg/mL
Lamin-B1 HRP	Abcam	Ab194109	0,5 µg/mL
MMP-13	Proteintech	18165-1-AP	1,5 µg/mL
NPC1	Novus Biologicals	NB400-148	10 µg/mL
Phospho-EGFR (Tyr1068)	Cell Signaling Technology	2234S	0,854 µg/mL
Secondary antibodies			
Alexa Fluor 488 donkey anti-mouse (Alexa Flour 488)	Invitrogen	A11006	2 µg/mL
Alexa Fluor 647 goat anti-rabbit IgG (Alexa Fluor 647)	Invitrogen	A21245	2 µg/mL
Goat Anti-rabbit IgG (H+L) HRP	Vector Laboratories	PI-1000	0,5 µg/mL
Polyclonal Rabbit Anti-mouse Immunoglobulin HRP	Dako	P0260	1,3 µg/mL
Fluorescent dyes and compounds			
Filipin	Sigma-Aldrich	F9768	1 ng/ml
NucRed Live 647	Thermo Fischer Scientific	R37106	1 drop/ml
Dextran 10 kD,Alexa Fluor 594	Invitrogen	D22913	150 µg/ml
BODIPY-Cholesterol	Avanti Polar Lipids	810255P	0,5 µM
Nuclear Violet LCSI	AAT Bioquest	17543	0,3 µM
Tubulin Tracker Deep Red	Thermo Fischer Scientific	T34076	0,2 µM
Dextran 70 kD,lysine fixable, Texas Red	Thermo Fischer Scientific	D1864	0,5 mg/ml
SiR-Tubulin	Spirochrome	SC002	0,2 µM
