# Supplementary material for: Activation of invasion by oncogenic reprogramming of cholesterol metabolism via increased NPC1 expression and macropinocytosis
Source: Oncogene. 2023 Jul 7;42(33):2495–506. doi: 10.1038/s41388-023-02771-x (PMC10421736; doi:10.1038/s41388-023-02771-x)
Supplement: Supplementary file 5 — Supplementary Table 4 [file 41388_2023_2771_MOESM5_ESM.pdf]

Supplementary Table 4: List of siRNA and PCR primers used in the study

**SiRNA Name**	Cat No	Brand
AllStars Negative Control (NTC siRNA)	1027281	Qiagen
MZF1 1	SASI_Hs01_00096726	Sigma-Aldrich
MZF1 2	SC-45714	Santa Cruz Biotechnologies
NPC1 #1	SASI_Hs01_00101265	Sigma-Aldrich
NPC1 #2	SASI_Hs01_00101266	Sigma-Aldrich

**ChIP primers**	Sequence 5´-3´	Brand
NPC1 (-880- -698 of TSS) forward	GCCAGGTGGACATCTCAGG	TAG Copenhagen
NPC1 (-880- -698 of TSS) reverse	TCTTTCCCAACAGTGAAAGCAAA	TAG Copenhagen
Lin28A (-4394- -4155 of TSS) forward	CCTGATAAATGCTGGCTGTC	TAG Copenhagen
Lin28A (-4394- -4155 of TSS) reverse	CAAGGGTGTCCGGTATCAACA	TAG Copenhagen

**qPCR primers**	Sequence 5´-3´	Brand
NPC1 forward	TTGGCTTGGACGCCATGTAT	TAG Copenhagen
NPC1 reverse	CAGGACGCCTCTCCTTTGTC	TAG Copenhagen
PPIB forward	GGGAGATGGCACAGGAGG	TAG Copenhagen
PPIB reverse	TGGGAGCCGTTGGTGTCT	TAG Copenhagen
